# Sex and Genotype Modulate the Dendritic Effects of Developmental Exposure to a Human-Relevant Polychlorinated Biphenyls Mixture in the Juvenile Mouse

**DOI:** 10.3389/fnins.2021.766802

**Published:** 2021-12-03

**Authors:** Kimberly P. Keil Stietz, Sunjay Sethi, Carolyn R. Klocke, Tryssa E. de Ruyter, Machelle D. Wilson, Isaac N. Pessah, Pamela J. Lein

**Affiliations:** ^1^Department of Molecular Biosciences, School of Veterinary Medicine, University of California, Davis, Davis, CA, United States; ^2^Clinical and Translational Science Center, Division of Biostatistics, Department of Public Health Sciences, School of Medicine, University of California, Davis, Davis, CA, United States

**Keywords:** FMR1 premutation, gene–environment interaction, Golgi stain, neurodevelopmental disorders, ryanodine receptor, Sholl analysis

## Abstract

While many neurodevelopmental disorders (NDDs) are thought to result from interactions between environmental and genetic risk factors, the identification of specific gene-environment interactions that influence NDD risk remains a critical data gap. We tested the hypothesis that polychlorinated biphenyls (PCBs) interact with human mutations that alter the fidelity of neuronal Ca^2+^ signaling to confer NDD risk. To test this, we used three transgenic mouse lines that expressed human mutations known to alter Ca^2+^ signals in neurons: (1) gain-of-function mutation in ryanodine receptor-1 (T4826I-*RYR1*); (2) CGG-repeat expansion in the 5′ non-coding portion of the fragile X mental retardation gene 1 (*FMR1*); and (3) a double mutant (DM) that expressed both mutations. Transgenic and wildtype (WT) mice were exposed throughout gestation and lactation to the MARBLES PCB mix at 0.1, 1, or 6 mg/kg in the maternal diet. The MARBLES mix simulates the relative proportions of the twelve most abundant PCB congeners found in serum from pregnant women at increased risk for having a child with an NDD. Using Golgi staining, the effect of developmental PCB exposure on dendritic arborization of pyramidal neurons in the CA1 hippocampus and somatosensory cortex of male and female WT mice was compared to pyramidal neurons from transgenic mice. A multilevel linear mixed-effects model identified a main effect of dose driven by increased dendritic arborization of cortical neurons in the 1 mg/kg PCB dose group. Subsequent analyses with genotypes indicated that the MARBLES PCB mixture had no effect on the dendritic arborization of hippocampal neurons in WT mice of either sex, but significantly increased dendritic arborization of cortical neurons of WT males in the 6 mg/kg PCB dose group. Transgene expression increased sensitivity to the impact of developmental PCB exposure on dendritic arborization in a sex-, and brain region-dependent manner. In conclusion, developmental exposure to PCBs present in the gestational environment of at-risk humans interfered with normal dendritic morphogenesis in the developing mouse brain in a sex-, genotype- and brain region-dependent manner. Overall, these observations provide proof-of-principle evidence that PCBs interact with heritable mutations to modulate a neurodevelopmental outcome of relevance to NDDs.

## Introduction

Despite a worldwide ban on the production of polychlorinated biphenyls (PCBs) since the early 2000’s, PCBs remain a significant risk to the developing human brain. Pregnant women and children continue to be exposed to not only legacy PCBs released from hazardous waste sites and PCB-containing equipment and materials manufactured prior to the PCB production ban, but also contemporary PCBs produced as inadvertent byproducts of contemporary pigment and dye production or via environmental degradation of legacy PCBs (Koh et al., 2015; Granillo et al., 2019). Human (Schantz et al., 2003; Berghuis et al., 2015; Pessah et al., 2019) and animal (Sable and Schantz, 2006; Klocke and Lein, 2020) studies provide compelling evidence of PCB developmental neurotoxicity, while recent epidemiologic studies suggest that developmental PCB exposures confer risk for NDDs, including autism spectrum disorder (ASD) and attention-deficit/hyperactivity disorder (ADHD) (Lyall et al., 2017; Pessah et al., 2019; Xi and Wu, 2021).

The size and shape of the neuronal dendritic arbor is a key structural determinant of neuronal connectivity, and changes in dendritic morphology (increased or decreased dendrite number, branching and/or spine density) contribute to the altered patterns of neuronal connectivity observed in many NDDs (Coskun et al., 2013; Keown et al., 2013; Khan et al., 2015; Alaerts et al., 2016; Cooper et al., 2017). The dynamic structural remodeling of dendrites and synapses that occurs during development is driven in large part by Ca^2+^-dependent signaling that mediates the influence of neural activity and other environmental factors on dendritic morphogenesis and plasticity (Cline, 2001; Konur and Ghosh, 2005; Chen and Nedivi, 2010). Many NDD risk genes encode proteins that regulate intracellular Ca^2+^ signals, are regulated by local fluctuations in Ca^2+^ concentrations and/or are involved in regulating dendritic growth and synaptogenesis (Krey and Dolmetsch, 2007; Pessah et al., 2010; Grove et al., 2019). Developmental exposure to Aroclor 1254, a commercial mixture of legacy PCBs, or to PCB 95 has been demonstrated to increase dendritic arborization in the hippocampus, cortex and cerebellum of experimental animal models (Roegge et al., 2006; Lein et al., 2007; Yang et al., 2009; Wayman et al., 2012b). *In vitro* studies have shown that the ryanodine receptor (RyR)-active PCB congeners PCB 95 and PCB 136 (Wayman et al., 2012b; Yang et al., 2014), and the lower chlorinated congener PCB 11 (Sethi et al., 2018), promote dendritic growth in primary hippocampal and cortical neurons via activation of Ca^2+^-dependent signaling pathways (Wayman et al., 2012a; Sethi et al., 2018) that map onto Ca^2+^-dependent signaling pathways implicated in the etiology of NDDs (Panesar et al., 2020). These observations suggest the possibility that PCBs amplify the risk and/or severity of NDDs by converging on signaling pathways altered by heritable defects in Ca^2+^-dependent signaling pathways that regulate dendritic arborization and/or plasticity.

To test this hypothesis, we compared the effect of developmental exposure to a human-relevant PCB mixture on the dendritic morphology of pyramidal neurons in the hippocampus and somatosensory cortex of wildtype (WT) *vs*. transgenic mice that expressed heritable human mutations that modulate the fidelity of neuronal Ca^2+^ signaling. Specifically, we examined three transgenic lines: (1) mice that carried a human *RYR1* gain-of-function mutation (T4826I-*RYR1*) (Barrientos et al., 2012; Yuen et al., 2012); (2) mice that expressed a CGG repeat expansion in the 5′ non-coding region of the fragile X mental retardation gene 1 (*FMR1*) in the premutation range (55–200 repeats) (Willemsen et al., 2003); and (3) mice that expressed both mutations (double mutant; DM) (Keil et al., 2019b). RyR Ca^2+^ ion channels regulate intracellular Ca^2+^ stores (Pessah et al., 2010) and their activation is required for activity-dependent dendritic growth and synaptogenesis (Wayman et al., 2012b; Lesiak et al., 2014). A genome wide association study identified *RYR1* and *RYR2* as ASD candidate genes by using sex as an additional risk factor (Lu and Cantor, 2012). *FMR1* premutation is causally linked to fragile X-associated tremor/ataxia syndrome (FXTAS) and is the most prevalent monogenic NDD risk factor (Krueger and Bear, 2011; Chonchaiya et al., 2012; Leehey and Hagerman, 2012). Unlike *FMR1* knockout models, these mice exhibit reduced FMR1 protein (FMRP) expression and elevated *Fmr1* mRNA (Berman et al., 2012; Robin et al., 2017). In a study examining GWAS and genetic databases, approximately 10% of FMRP targets in the brain overlap with ASD candidate genes, many of which regulate neuronal connectivity (Fernandez et al., 2013). Studies of primary neurons derived from *FMR1* premutation knockin mice (referred to hereafter as CGG mice) demonstrate resting intracellular Ca^2+^ concentrations threefold higher than neurons derived from WT (Robin et al., 2017), and abnormal patterns of intracellular Ca^2+^ oscillations including increased number of spontaneous Ca^2+^ burst activity (Cao et al., 2012). iPSC-derived neurons from an *FMR1* premutation carrier also exhibited enhanced Ca^2+^ transients (Liu et al., 2012). Altered dendritic arborization and spine density are linked with these changes in Ca^2+^ dynamics in both primary neurons from *FMR1* premutation mice (Chen et al., 2010), and iPSC-derived neurons from humans with *FMR1* premutation (Liu et al., 2012).

In addition to the two transgenic lines expressing either a RYR1 gain-of-function mutation or *FMR1* premutation, we examined a transgenic line (DM) that expressed both mutations (Keil et al., 2019b). Expressed variants in RyR1 and FMR1 expansion repeats in the premutation range are relatively common mutations in the human population. Approximately 15% of the human population is estimated to carry one or more RYR1 genetic variants (Kim et al., 2013), whereas, the estimated prevalence of the FMR1 premutation in the human population is 1:209 in females and 1:430 in males (Tassone et al., 2012). Both mutations are phenotypically silent until triggered by halogenated anesthetics (RYR1 gain-of-function) or advancing age (*FMR1* premutation). Thus, while we are not aware of any clinical reports of human patients expressing mutations at both loci, there is a reasonable likelihood that there are individuals who carry both mutagens. Regardless, these DM mice were not created to mimic a human disease, but rather as an experimental model to investigate whether gene dosage influences the effects of developmental PCB exposures. In other words, is the phenotypic outcome amplified when two mutations that converge on calcium signaling and regulation of dendritic growth are expressed relative to expression of either mutation alone. The *RYR1* mutation was chosen as a direct target of PCBs (Ta and Pessah, 2007); whereas the FMR1 premutation was chosen because of its demonstrated role in translational control of calcium regulating proteins (Robin et al., 2017). Our earlier characterization of dendritic arborization in juvenile male and female mice from these three transgenic lines revealed significantly increased dendritic arborization of pyramidal neurons in the CA1 hippocampus of male T4826I-*RYR1* and, to a lesser extent, male CGG mice relative to male congenic WT mice. Dendritic arborization of pyramidal neurons in the somatosensory cortex was significantly enhanced in male and female CGG and DM mice compared to WT mice with the most pronounced differences seen in DM females (Keil et al., 2019b).

In this study, we exposed WT, T4826I, CGG and DM mice to vehicle or the MARBLES PCB mixture (Sethi et al., 2019) in the maternal diet throughout gestation and lactation. The MARBLES PCB mixture proportionally mimics the top twelve PCB congeners detected in the serum of pregnant women enrolled in the MARBLES cohort (Granillo et al., 2019; Sethi et al., 2019) who are at increased risk of having a child with an NDD (Hertz-Picciotto et al., 2018). We previously demonstrated that the MARBLES PCB mix has RyR activity *in vitro* at low micromolar concentrations, reflecting the small percentage of PCB congeners with potent RyR activity (Sethi et al., 2019). This is consistent with epidemiological evidence that RyR-active PCBs are associated with increased risk of ASD (Granillo et al., 2019). Our findings indicate that expression of heritable mutations that alter the fidelity of neuronal Ca^2^*^+^* signals modulated the impact of PCB exposure on several parameters of dendritic arborization in a sex- and brain region-dependent manner.

## Materials and Methods

### Materials

Organic unsalted peanut butter (Trader Joe’s, Monrovia, CA, United States) and organic peanut oil (Spectrum Organic Products, LLC, Melville, NY, United States) were purchased from Trader Joe’s (Davis, CA, United States). The individual PCB congeners (PCB 11, 28, 52, 84, 95, 101, 118, 135, 138, 149, 153, and 180) used to make the MARBLES PCB mix were synthesized and authenticated as previously described (Li et al., 2018; Sethi et al., 2019). The purity of all PCB congeners was > 99% pure (Sethi et al., 2019).

### Animals

All procedures involving animals were conducted in accordance with the NIH Guide for the Care and Use of Laboratory Animals, conformed to the ARRIVE guidelines (Kilkenny et al., 2010), and were approved by the University of California, Davis Institutional Animal Care and Use Committee. Male and female mice were derived from transgenic mouse colonies maintained at UC Davis (Keil et al., 2019b), which included transgenic strains: (1) homozygous for the human gain-of-function mutation in *RYR1* (T4826I-*RYR1*) referred to as T4826I mice, (2) homozygous (female) or hemizygous (male) for the X-linked CGG repeat expansion in *FMR1* in the permutation range (170-200 repeats; referred to as CGG mice); and (3) DM mice that expressed both mutations (Keil et al., 2019b). C57Bl/6J and SVJ129 WT mice were purchased from Jackson Labs (Sacramento, CA, United States) and crossed to generate a 75% C57Bl/6J / 25% SVJ129 congenic WT line that matched the genetic background of the T4826I, CGG and DM animals as determined by single-nucleotide polymorphism (SNP) analysis (Keil et al., 2019b). Homo/hemizygous matings were used to generate the juvenile mice used for Golgi analyses, and all animals used in this study were genotyped as previously described (Keil et al., 2019b).

All animals were housed in clear plastic shoebox cages containing corn cob bedding and maintained on a 12 h light and dark cycle at 22 ± 2°C with 40–50% humidity. Feed (Diet 5058, LabDiet, Saint Louis, MO, United States) and water were available *ad libitum*. Two weeks prior to mating, nulliparous and previously unmated dams (>6 weeks of age) were singly housed and PCB dosing was initiated. Dams were placed with a genotype-matched male overnight for mating. Males and females were separated the next day and females were checked for the presence of a copulatory plug, which was considered gestational day 0. After mating, dams were housed singly prior to parturition and with their pups after parturition. At postnatal day 2 (P2), pups were culled or cross-fostered within genotype- and dose-matched litters to ensure all litters consisted of 4–8 pups. After weaning at P21, pups were group housed with same-sex littermates. Mice underwent self-grooming and social approach behavioral testing as part of a larger study, and then were euthanized on P27–31 to collect brains for Golgi analyses.

This study is part of an overall study designed to assess the effects of developmental exposure to the MARBLES PCB mixture on multiple developmental outcomes, including NDD-relevant behavioral phenotypes (data under review), the gut microbiome and intestinal physiology (Rude et al., 2019) and cytokine levels in the serum and hippocampus (Matelski et al., 2020). The data described in this study were collected from animals used for behavioral studies prior to being euthanized to harvest brains for morphometric analyses of dendritic arborization. We previously reported that developmental exposure to the MARBLES PCB mixture had no effect on the length of time from mating to parturition and pregnancy rates across groups averaged 88% (Matelski et al., 2020). While dam weight at weaning was not altered by PCB exposure, there was a significant main effect of genotype, with DM dams weighing significantly more than WT dams, T4826I dams weighing significantly more than CGG dams, and CGG dams weighing significantly less than DM dams (Matelski et al., 2020). We also found that there were no effects of developmental PCB exposure or genotype on litter size or sex ratio within the litter (data under review).

### Developmental Polychlorinated Biphenyls Exposures

The MARBLES PCB mixture was prepared to proportionally mimic the serum PCB congener profile of the twelve most prevalent PCB congeners detected in serum of pregnant women enrolled in the MARBLES human epidemiological cohort (Granillo et al., 2019; Sethi et al., 2019). These women are at increased risk for having a child with an NDD (Hertz-Picciotto et al., 2018). The PCB congeners included in the MARBLES PCB mixture and their final total percentage in the mixture was as follows: PCB 28 (48.2%), PCB 11 (24.3%), PCB 118 (4.9%), PCB 101 (4.5%), PCB 52 (4.5%), PCB 153 (3.1%), PCB 180 (2.8%), PCB 149 (2.1%), PCB 138 (1.7%), PCB 84 (1.5%), PCB 135 (1.3%), and PCB 95 (1.2%). The MARBLES PCB mix was solubilized in peanut oil and homogenously mixed into peanut butter to achieve concentrations of 0.025, 0.25, and 1.5 mg PCB/g peanut butter. A vehicle control (0 mg/g) was similarly prepared by mixing the equivalent amount of peanut oil needed to solubilize the highest concentration of MARBLES mix into peanut butter. Two weeks prior to mating, nulliparous dams (>6 weeks of age) were randomized to dose groups and PCB exposures were initiated. Dams were fed the MARBLES PCB mix in peanut butter at doses of either 0, 0.1, 1 or 6 mg/kg_BW_/day daily until pups were weaned at P21. Similar doses of Aroclor 1254 were previously shown to result in PCB body burdens comparable to those observed in human tissues (Yang et al., 2009). At each daily dosing, dams were monitored to ensure complete ingestion of each dose of peanut butter.

### Golgi Staining

Golgi staining, image acquisition, and analysis were performed as described previously (Keil et al., 2017, 2019b; Wilson et al., 2017). Parameters used to assess Golgi staining and criteria for selecting Golgi-stained neurons to trace were described previously (Lein et al., 2007; Keil et al., 2017). Briefly, P27–31 pups were euthanized with CO_2._ Brains were carefully and quickly extracted from the skulls and processed for Golgi staining using the FD Rapid GolgiStain kit (FD NeuroTechnologies Inc. Columbia, MD, United States) according to the manufacturer’s instructions. Brightfield image stacks of pyramidal neurons in the CA1 of the hippocampus and layers IV/V of the somatosensory cortex were captured using an Olympus IX-81 inverted confocal microscope (Olympus, Shinjuku, Japan) at 20X magnification using MetaMorph Advanced image analysis software (version 7.1, Molecular Devices, Sunnyvale, CA, United States). These brain regions were chosen because they contain easily identifiable pyramidal neurons and are implicated in the pathogenesis of neurodevelopmental disorders (Coskun et al., 2013; Khan et al., 2015; Cooper et al., 2017). Neuronal basilar dendritic arbors (*N* = 39–49 hippocampal neurons per group and *N* = 44–48 cortical neurons per group derived from six mice per sex, genotype, and exposure group) were hand-traced by a single individual blinded to experimental group using Neurolucida (version 11, MBF Bioscience, Williston, VT, United States). Basilar dendritic arbors in these regions were chosen for analysis because previous studies of PCB effects on dendritic arborization demonstrated the developmental exposure to Aroclor 1254 or PCB 95 altered basilar dendrites (Lein et al., 2007; Yang et al., 2009; Wayman et al., 2012b). Dendritic arbor complexity was quantified using automated Sholl (Neurolucida Explorer, version 11, MBF Bioscience) with 10-μm Sholl rings centered on the neuronal soma. Neuron tracings are publicly available on the NeuroMorpho.Org database^1^.

### Statistical Analyses

Sholl curves for each neuron were assessed using a multilevel linear mixed-effects model to determine effects of genotype, sex, dose or interactions on dendritic arborization; these analyses were conducted using SAS software (version 9.4, SAS Institute Inc., Cary, NC, United States) as described previously (Keil et al., 2017, 2019b; Wilson et al., 2017). In the multi-level linear mixed-effects modeling, genotype, sex, and dose were treated as fixed effects. A random intercept was included in the model to control for clustering of observations within a neuron and neurons within animals. Log transformation was applied when necessary (as indicated in Tables 1, 2). Tables 1, 2 report tests for fixed effects and differences of least squares means for any fixed effects with *p* ≤ 0.05 as well as for any fixed effects that were approaching significance (*p* < 0.1) and also had significant effects as identified in the differences of least squares means. Supplementary Data Files report the SAS output, including the solution for fixed effects, fixed effects, least squares means and differences of least squares means. Area under the curve (AUC), distance from soma of the peak dendritic intersections (Peak X), and maximum number of dendritic intersections (Peak Y) values were calculated for Sholl profiles using AUC analysis in GraphPad Prism Software (version 6 and 7, San Diego, CA, United States) for each neuron. To allow for comparisons to earlier studies that did not use mixed-effects models (Roegge et al., 2006; Lein et al., 2007; Yang et al., 2009; Wayman et al., 2012b), PCB-induced differences between neurons within sex and genotypes were independently examined using GraphPad Prism Software. These data were first assessed for normality using the Shapiro–Wilks, KS and D’Agostino and Pearson omnibus normality test, and homogeneity of variance using Bartlett’s test. Within each sex and genotype, significant differences between PCB dose groups were determined using one-way ANOVA followed by Dunnett’s or Tukey’s multiple comparison test for approximately normal data. If data were normal but had unequal variance, group differences were determined using a one-way ANOVA with Welch’s correction followed by Dunnett’s T3 multiple comparisons test. For non-normal data, differences were determined using a Kruskal–Wallis test followed by Dunn’s multiple comparison test. We first focused on differences from vehicle control, if there were no differences from vehicle control then differences between PCB groups were examined. *P*-values ≤ 0.05 were considered statistically significant. In two instances, the p value of the Kruskal–Wallis tests were 0.0591 and 0.0565, Dunn’s *post hoc* analysis revealed significant differences (*p* = 0.04 and *p* = 0.03), so these were reported in Figures 2E, 3A respectively.

**TABLE 1:** Summary of mixed model effects in pyramidal CA1 hippocampal neurons.

	Tests of fixed effects	*p*-value	Differences of least squares means	*p*-value
Sholl Profile	Sex Genotype Dose	0.5 0.4 0.5		

Peak X	Sex	0.5	**CGG > T4826I**	0.04
	Genotype Dose	0.08 0.9	**CGG > WT**	0.03

Peak Y	Sex	0.9		
	**Genotype** Dose	0.3 **0.03**	**DM < WT** **T4826I < WT**	0.02 0.008

Total area under Sholl curve	Sex Genotype Dose	0.8 0.4 0.5		

Proximal area under Sholl curve	Sex Genotype Dose	0.9 0.2 0.3		

Distal area under Sholl curve (log)	Sex Genotype Dose	0.6 0.4 0.8		
	Sex*Genotype	**0.03**	**CGG F > DM F** **CGG F > WT F**	0.04 0.01
			**CGG F > CGG M**	0.04
			CGG F > T4826I M	0.01
			**DM M > T4826I M**	0.03
			DM M > WT F	0.05

Number of dendrites	Sex	0.7		
	**Genotype** Dose	**0.004** 0.4	**CGG < WT** **DM < WT**	0.02 0.0009

			**T4826I < WT**	0.003
Terminal dendritic tips	Sex Genotype Dose	0.8 0.1 0.6	**T4826I < WT**	0.05

Sum dendritic length	Sex Genotype Dose	0.7 0.3 0.5		

Tips per dendrite	Sex Genotype Dose	0.8 0.3 0.5		
Mean dendritic length	Sex Genotype Dose	0.4 0.2 0.4		

Nodes	Sex Genotype Dose	0.9 0.2 0.7		

Soma area	Sex	0.09		
	**Genotype**	**<0.0001**	**CGG > DM** **CGG > T4826I**	0.003 0.002
			**CGG 0.1 < WT 0** **DM < WT**	<0.0001 <0.0001
	Dose Genotype*Dose	0.4 **0.03**	**T4826I < WT**	0.01
			CGG 0.1 > DM 1	0.03
			**CGG 1 > DM 1**	0.02
			**CGG 1 < WT 0**	0.01
			**CGG 6 > DM 0**	0.05
			CGG 6 > DM 1	0.006
			CGG 6 > T4826I 1	0.04
			**CGG 6 > T4826I 6**	0.05
			**CGG 6 < WT 0**	0.04
			**CGG 0 > DM 0**	0.02
			CGG 0 > DM 0.1	0.04
			CGG 0 > DM 1	0.002
			CGG 0 > T4826I 1	0.02
			CGG 0 > T4826I 6	0.02
			**DM 0.1 < WT 0.1**	0.02
			DM 0.1 < WT 1	0.01
			**DM 0.1 < WT 0**	0.0002
			**DM 1 < DM 6**	0.01
			DM 1 < WT 0.1	0.001
			**DM 1 < WT 1**	0.0004
			**DM 1 < WT 0**	<0.0001
			**DM 6 < WT 0**	0.03
			DM 0 < WT 0.1	0.01
			**DM 0 < WT 1**	0.006
			**DM 0 < WT 0**	<0.0001
			**T4826I 0.1 < WT 0.1**	0.04
			T4826I 0.1 < WT 1	0.02
			**T4826I 0.1 < WT 0**	0.0004
			T4826I 1 < WT 0.1	0.009
			**T4826I 1 < WT 1**	0.004
			**T4826I 1 < WT 0**	<0.0001
			T4826I 6 < WT 0.1	0.01
			T4826I 6 < WT 1	0.006
			**T4826I 6 < WT 0**	<0.0001
			T4826I 0 < WT 0.1	0.05
			T4826I 0 < WT 1	0.03
			**T4826I 0 < WT 0**	0.0007
			**WT 6 < WT 0.1**	0.03
			**WT 6 < WT 1**	0.02
			**WT 6 < WT 0**	0.0004

*Bold text indicates biologically relevant comparisons.*

**TABLE 2:** Summary of mixed model effects in pyramidal cortical neurons.

	Test of fixed effects	*p*-value	Differences of least squares means	*p*-value
Sholl profile	Sex Genotype	0.6 0.8		
	**Dose**	**0.03**	**1 mg/kg > 6 mg/kg** **1 mg/kg > 0 mg/kg**	0.004 0.02
	Genotype*Dose	**0.01**	**CGG 6 < CGG 1**	0.05
			**CGG 6 < CGG 0**	0.003
			**CGG 6 < WT 6**	0.05
			CGG 6 < DM 1	0.0005
			CGG 6 < T4826I 0.1	0.04
			CGG 6 < T4826I 1	0.02
			**CGG 0 > WT 0**	0.01
			**CGG 0 > DM 0**	0.03
			CGG 0 > DM 0.1	0.01
			CGG 0 > DM 6	0.01
			**CGG 0 > T4826I 0**	0.008
			CGG 0 > T4826I 6	0.03
			DM 0.1 < T4826I 1	0.05
			**DM 1 > DM 0.1**	0.002
			**DM 1 > DM 6**	0.002
			**DM 1 > DM 0**	0.008
			DM 1 > WT 0.1	0.04
			**DM1 > WT 1**	0.02
			**DM 1 > WT 0**	0.002
			DM 1 > T4826I 6	0.008
			**DM 1 > T4826I 0**	0.001
			**T4826I 1 > T4826I 0**	0.03
			**T4826I 1 > WT 0**	0.04
			T4826I 1 > DM 6	0.04

Peak X	Sex Genotype Dose	0.8 0.5 0.6		

Peak Y	Sex Genotype	0.4 0.6		
	**Dose**	**0.02**	**1 mg/kg > 0.1 mg/kg** **1 mg/kg > 6 mg/kg**	0.05 0.002
			**1 mg/kg > 0 mg/kg**	0.02

Total area under Sholl curve	Sex Genotype	0.7 0.8		
	**Dose**	**0.02**	**1 mg/kg > 6 mg/kg** **1 mg/kg > 0 mg/kg**	0.003 0.02
	Genotype*Dose	**0.01**	**CGG 6 < CGG 1**	0.05
			**CGG 6 < CGG 0**	0.004
			**CGG 6 < WT 6**	0.05
			CGG 6 < DM 1	0.0007
			CGG 6 < T4826I 0.1	0.05
			CGG 6 < T4826I 1	0.02
			**CGG 0 > WT 0**	0.01
			**CGG 0 > DM 0**	0.03
			CGG 0 > DM 0.1	0.01
			CGG 0 > DM 6	0.007
			**CGG 0 > T4826I 0**	0.009
			CGG 0 > T4826I 6	0.04
			DM 0.1 < T4826I 1	0.05
			**DM 1 > DM 0.1**	0.003
			**DM 1 > DM 6**	0.001
			**DM 1 > DM 0**	0.007
			DM 1 > WT 0.1	0.04
			**DM 1 > WT 1**	0.02
			**DM 1 > WT 0**	0.002
			DM 1 > T4826I 6	0.009
			**DM 1 > T4826I 0**	0.002
			**T4826I 1 > T4826I 0**	0.04
			**T4826I 1 > WT 0**	0.04
			T4826I 1 > DM 6	0.03

Proximal area under Sholl curve	Sex Genotype	0.4 0.7		
	**Dose**	**0.02**	**1 mg/kg > 0.1 mg/kg** **1 mg/kg > 6 mg/kg**	0.05 0.003
			**1 mg/kg > 0 mg/kg**	0.03
	Genotype*Dose	**0.02**	CGG 1 > DM 6	0.03
			**CGG 6 < CGG 1**	0.03
			**CGG 6 < CGG 0**	0.004
			**CGG 6 < WT 6**	0.03
			CGG 6 < T4826I 0.1	0.05
			CGG 6 < T4826I 1	0.009
			CGG 6 < DM 1	0.002
			**CGG 0 > WT 0**	0.01
			**CGG 0 > DM 0**	0.04
			CGG 0 > DM 0.1	0.02
			CGG 0 > DM 6	0.003
			**CGG 0 > T4826I 0**	0.02
			CGG 0 > T4826I 6	0.05
			**DM 1 > DM 0.1**	0.009
			**DM 1 > DM 6**	0.002
			**DM 1 > DM 0**	0.02
			DM 1 > WT 0.1	0.05
			**DM 1 > WT 1**	0.05
			**DM 1 > WT 0**	0.007
			DM 1 > T4826I 6	0.03
			**DM 1 > T4826I 0**	0.008
			**DM 6 < WT 6**	0.03
			DM 6 < T4826I 0.1	0.05
			**T4826I 1 > T4826I 0**	0.03
			**T4826I 1 > WT 0**	0.03
			T4826I 1 > DM 0.1	0.04
			T4826I 1 > DM 6	0.008

Distal area under Sholl curve (log)	Sex Genotype Dose	0.3 0.7 0.3		
	Genotype*Dose	**0.06**	**CGG 6 < CGG 0** CGG 6 < DM 1	0.03 0.01
			CGG 0 > WT 1	0.03
			**CGG 0 > DM 0**	0.05
			CGG 0 > DM 0.1	0.05
			CGG 0 > DM 6	0.05
			**CGG 0 > T4826I 0**	0.03
			**CGG 0 > WT 0**	0.03
			**DM 1 > DM 0.1**	0.02
			**DM 1 > DM 6**	0.02
			**DM 1 > DM 0**	0.02
			**DM 1 > WT 1**	0.01
			**DM 1 > WT 0**	0.01
			DM 1 > T4826I 6	0.05
			**DM 1 > T4826I 0**	0.01

Number of dendrites	Sex Genotype	0.7 1		
	**Dose**	**0.01**	**0.1 mg/kg < 1 mg/kg** **1 mg/kg > 6 mg/kg**	0.05 0.005
			**1 mg/kg > 0 mg/kg**	0.003

Terminal dendritic tips	Sex Genotype	0.2 0.8		
	**Dose**	**0.0007**	**0.1 mg/kg < 1 mg/kg** **1 mg/kg > 6 mg/kg**	0.003 0.0001
			**1 mg/kg > 0 mg/kg**	0.003

Sum dendritic length	Sex Genotype	0.6 0.8		
	**Dose**	**0.01**	**0.1 mg/kg < 1 mg/kg** **1 mg/kg > 6 mg/kg**	0.05 0.002
			**1 mg/kg > 0 mg/kg**	0.01
	Genotype*Dose	**0.02**	CGG 0.1 < DM 1	0.05
			CGG 1 > DM 6	0.05
			**CGG 6 < CGG 1**	0.04
			**CGG 6 < CGG 0**	0.006
			CGG 6 < DM 1	0.0006
			CGG 6 < T4826I 1	0.02
			**CGG 0 > WT 0**	0.01
			**CGG 0 > DM 0**	0.04
			CGG 0 > DM 0.1	0.02
			CGG 0 > DM 6	0.008
			**CGG 0 > T4826I 0**	0.008
			CGG 0 > T4826I 6	0.04
			DM 0.1 < T4826I 1	0.05
			**DM 1 > DM 0.1**	0.002
			**DM 1 > DM 6**	0.0009
			**DM 1 > DM 0**	0.006
			DM 1 > WT 0.1	0.03
			**DM 1 > WT 1**	0.02
			**DM 1 > WT 0**	0.002
			**DM 1 > T4826I 0**	0.001
			DM 1 > T4826I 6	0.007
			DM 6 < T4826I 1	0.03
			**T4826I 1 > T4826I 0**	0.03
			**T4826I 1 > WT 0**	0.04

Tips per dendrite	Sex Genotype	0.2 0.3		
	Dose	0.1	**0.1 mg/kg < 1 mg/kg** **1 mg/kg > 6 mg/kg**	0.05 0.03

Mean dendritic length	Sex Genotype Dose	0.7 0.4 0.4		

Nodes	Sex Genotype	0.2 0.6		
	**Dose**	**0.002**	**0.1 mg/kg < 1 mg/kg** **1 mg/kg > 6 mg/kg**	0.003 0.0004
			**1 mg/kg > 0 mg/kg**	0.02

Soma area	Sex	0.7		
	**Genotype**	**0.03**	**CGG > DM** **CGG > T4826I**	0.04 0.01
			**T4826I < WT**	0.03
	**Dose**	**0.05**	**0.1 mg/kg > 0 mg/kg**	0.008
			**1 mg/kg > 0 mg/kg**	0.04

*Bold text indicates biologically relevant comparisons.*

## Results

Pyramidal CA1 hippocampal neurons and layer IV/V pyramidal somatosensory cortical neurons were examined in this study because altered patterns of connectivity and dendritic morphology have been reported in these brain regions in individuals with ASD compared to neurotypical controls (Coskun et al., 2013; Keown et al., 2013; Khan et al., 2015; Cooper et al., 2017). Results from the multilevel, mixed-effects statistical model, which includes interactions and allows for the analysis of the Sholl plot as a whole, are summarized in Tables 1, 2 with biologically relevant comparisons highlighted in bold. Within each subsection of the Results below, these results are discussed first. Subsequently, we describe PCB effects that are significantly different from vehicle control within each sex and genotype independently to allow for interpretation of PCB effects alone and to allow for comparisons to published studies that did not use mixed-effects models (Roegge et al., 2006; Lein et al., 2007; Yang et al., 2009; Wayman et al., 2012b).

### Morphometric Effects of Polychlorinated Biphenyls and Genotype on Pyramidal CA1 Hippocampal Neurons

**FIGURE 1:**
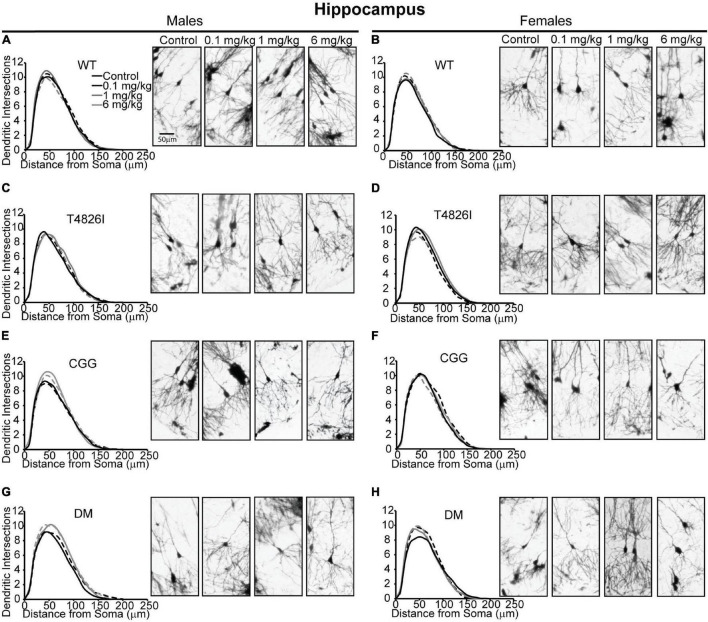
Genotype influences the effect of PCBs on the dendritic morphology of pyramidal neurons in the CA1 hippocampus of juvenile mice. Sholl plots and representative images of the basilar dendritic arbors of Golgi-stained pyramidal CA1 hippocampal neurons derived from P27–31 male and female **(A,B)** WT, **(C,D)** T4826I, **(E,F)** CGG, or **(G,H)** DM mice exposed to the MARBLES PCB mixture in the maternal diet throughout gestation and lactation. *N* = 39–49 neurons from at least six independent mice per sex per genotype per dose. Also see Supplementary Figure 1 for representative tracings of the basilar dendritic arbors of Golgi-stained pyramidal hippocampal neurons.

Sholl plots and representative images of basilar dendritic arbors of Golgi-stained pyramidal CA1 hippocampal neurons from male and female WT, T4826I, CGG and DM mice at P27-P31 are shown in Figure 1 (see also Supplementary Figures 1, 2; Neurolucida reconstructions are publicly available within the neuromorpho.org database). While the Sholl profile analysis revealed no significant effects (Table 1), other parameters extracted from the Sholl profile revealed sex and genotype effects. The distance from the soma of the maximum number of dendritic intersections (Peak X) was significantly greater in CGG mice than T4826I or WT mice (Table 1). There was an overall effect of genotype on the maximum number of dendritic intersections (Peak Y), with DM and T4826I mice exhibiting significantly fewer intersections than WT mice (Table 1). While the total area under the Sholl curve was not changed, there was a significant sex by genotype interaction for the distal AUC with this parameter being significantly greater in CGG female mice compared to WT and DM females or CGG males (Table 1). Distal area under the Sholl curve was also greater in DM males vs. T4826I males (Table 1). Overall, these results suggest pyramidal CA1 hippocampal neurons from CGG mice are more complex than those of WT mice, while pyramidal CA1 hippocampal neurons from T4826I and DM mice are less complex than their WT counterparts, and hippocampal neurons of DM males are more complex than T4826I males.

**FIGURE 2:**
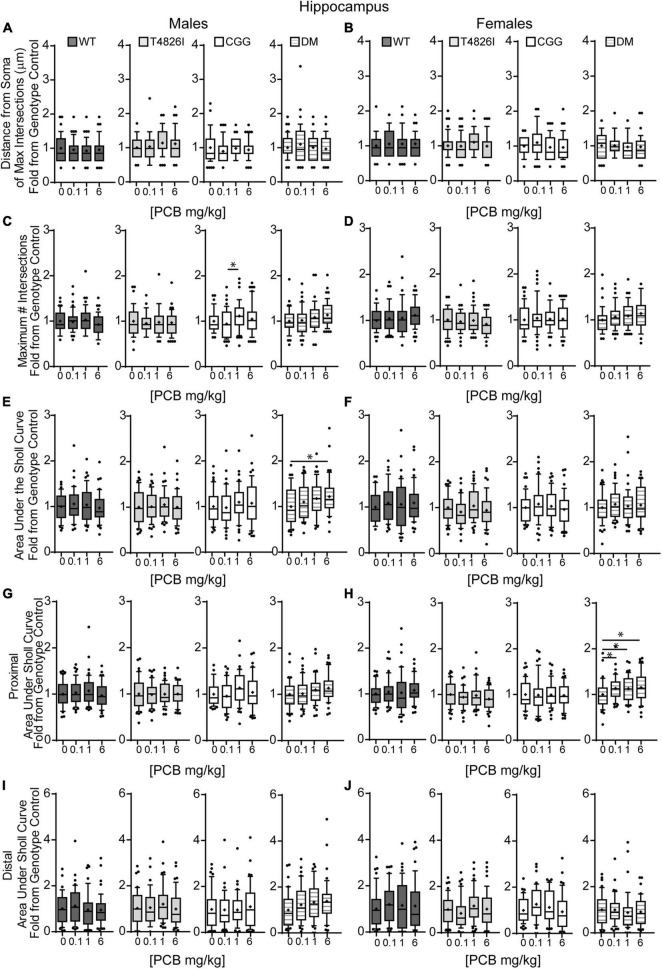
Double mutant mice are more sensitive to effects of PCBs on the dendritic morphology of pyramidal neurons in the CA1 hippocampus of juvenile mice. Morphometric analyses of the basilar dendritic arbors of Golgi-stained pyramidal CA1 hippocampal neurons from P27–31 male and female WT, T4826I, CGG, or DM mice exposed to the MARBLES PCB mixture in the maternal diet throughout gestation and lactation. **(A,B)** The distance from the soma to the maximum number of dendritic intersections (Peak X). **(C,D)** The maximum number of dendritic intersections (Peak Y). **(E,F)** The total area under the curve of the Sholl plot (0–240 μm from the soma). **(G,H)** Proximal area under the curve of the Sholl plot (10–70 μm from the soma), and **(I,J)** distal area under the curve of the Sholl plot (80–140 μm from the soma). Data (*N* = 39–49 neurons from at least six independent mice per sex per genotype per dose) are presented as box plots, where the box indicates the lower (25th) to upper (75th) quartiles, the “+” indicates the mean, whiskers indicate the 10–90th percentile, and dots represent values outside the upper or lower fences. *Significantly different from control at *p* ≤ 0.05 as determined by Kruskal–Wallis test followed by Dunn’s multiple comparisons test. Average values of vehicle controls for WT, T4826I, CGG and DM, respectively, are **(A)** 46.9, 40.8, 47.9, 47.3 μm; **(B)** 42.3, 44.9, 48.5, 46.4 μm; **(C)** 11.9, 10.8, 10.8, 10.4 intersections; **(D)** 10.8, 11.3, 11.2, 10.1 intersections; **(E)** 773.8, 698.2, 727.7, 713.8 μm^2^; **(F)** 721.1, 787.2, 807.8, 715.1 μm^2^; **(G)** 493.7, 454.1, 456.9, 450.5 μm^2^; **(H)** 466.7, 497.2, 492.1, 420.3 μm^2^; **(I)** 192.2, 164.6, 188.6, 178.8 μm^2^; and **(J)** 170.8, 197.4, 216.5, 202.6 μm^2^.

We next asked whether developmental PCB exposure alters dendritic arborization by focusing on PCB dose-response relationships within each sex and genotype independently (Figure 2). We focused on difference from vehicle control; if there were no difference from vehicle control then differences between PCB groups were analyzed. There were no effects of PCB exposure on distance from the soma of the maximum number of dendritic intersections (Peak X) in male or female hippocampal neurons of any genotype (Figures 2A,B). The maximum number of dendritic intersections (Peak Y) was increased in the 1 mg/kg PCB group *vs*. the 0.1 mg/kg PCB group in male CGG hippocampal neurons (Figures 2C,D). Total area under the Sholl curve was increased in the 6 mg/kg PCB dose group *vs*. vehicle control in male DM hippocampal neurons (Figures 2E,F). Differences in the proximal AUC of the Sholl plot were limited to female hippocampal neurons with a significant dose-dependent increase in DM female neurons in all PCB dose groups compared to vehicle controls (Figure 2H). There were no PCB effects in distal area under the Sholl curve for either sex in any genotype (Figures 2I,J). These results suggest that compared to vehicle control, PCBs increase dendritic complexity in DM mice only, an effect which is sex- and dose-dependent.

**FIGURE 3:**
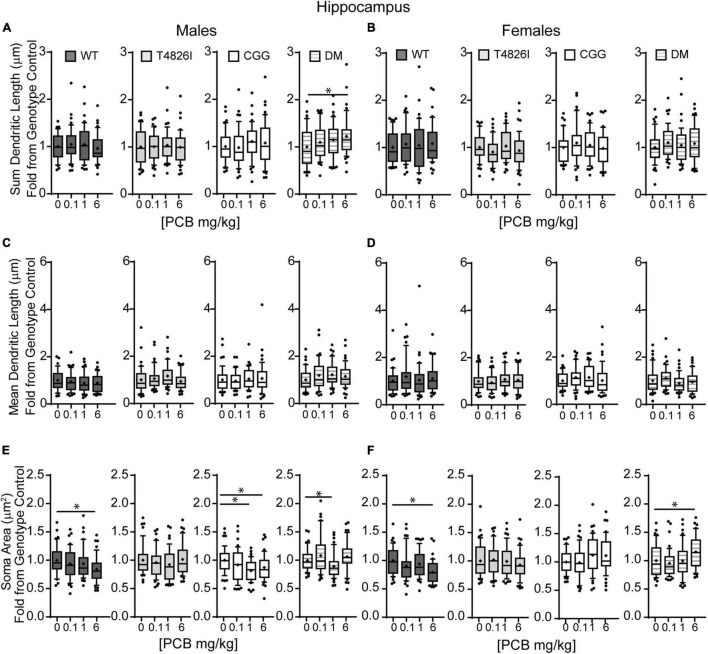
Polychlorinated biphenyls effects on dendritic sum length are observed in DM male but not female mice and both genotype and PCB exposure impact soma area in both sexes. Dendritic morphology was assessed in basilar dendritic arbors of Golgi-stained pyramidal CA1 hippocampal neurons in P27–31 male and female WT, T4826I, CGG, or DM mice following gestational and lactational PCB exposure via the maternal diet. **(A,B)** Sum length of dendrites per neuron (μm). **(C,D)** Mean dendritic length per neuron (μm). **(E,F)** Cell body area per neuron (μm^2^). Data (*N* = 39–49 neurons from at least six independent mice per sex per genotype per dose) are presented as box plots, where the box indicates the lower (25th) to upper (75th) quartiles, the “+” indicates the mean, whiskers indicate the 10–90th percentile, and dots represent values outside the upper or lower fences. Bar and asterisk indicate a significant difference between groups as determined by Kruskal–Wallis test followed by Dunn’s multiple comparisons test, or (E-CGG) one-way ANOVA followed by Dunnett’s multiple comparisons test with *p* ≤ 0.05 considered statistically significant. Average values of vehicle controls for WT, T4826I, CGG, and DM, respectively, are **(A)** 898.8, 804.8, 833.2, 815.2 μm; **(B)** 833.1, 903.0, 909.5, 801.5 μm; **(C)** 256.4, 215.7, 236.5, 228.3 μm; **(D)** 214.4, 244.9, 241.9, 245.9 μm; **(E)** 283.2, 246.3, 284.5, 230.2 μm^2^; and **(F)** 305.9, 240.3, 253.5, 238.8 μm^2^.

We also analyzed more detailed measures of dendritic arborization (Keil et al., 2017) that have previously been shown to be sensitive to PCBs (Lein et al., 2007; Yang et al., 2009; Wayman et al., 2012b). Based on the mixed-effect model analysis, effects were limited to genotype for the total number of basilar dendrites with pyramidal CA1 hippocampal neurons of all transgenic mice having fewer dendrites compared to WT (Table 1). Examining each sex and genotype independently, there were no effects of developmental PCB exposure on the number of primary dendrites, dendritic tips, or the number of dendritic tips per primary dendrite (Supplementary Figures 3A–F). The sum length of all dendrites was increased in the 6 mg/kg male DM neurons vs. sex- and genotype-matched vehicle control (Figures 3A,B). Mean dendritic length was unchanged (Figures 3C,D). These results suggest that genotype alone decreases dendrite number and PCBs only increase sum dendritic length in DM males exposed to the highest PCB dose.

Soma area was the morphometric parameter most affected by both genotype and developmental PCB exposure in pyramidal CA1 hippocampal neurons. Table 1 illustrates the statistically significant effect of genotype and significant genotype by dose interactions, with DM and T4826I mice displaying a smaller soma size than WT and CGG mice. Genotype by dose interactions were seen in WT neurons, with soma size significantly decreased in the 6 mg/kg group *vs*. WT vehicle control and other PCB dose groups (Table 1). T4826I and DM mice had smaller soma size than WT controls regardless of exposure (Table 1). Additionally, in DM mice, soma size was significantly decreased in the 1 mg/kg dose group compared to the 6 mg/kg group (Table 1). Soma size of CGG vehicle controls did not differ from WT vehicle controls, but hippocampal neurons of CGG mice at all PCB concentrations had smaller soma size than WT vehicle controls (Table 1). Overall, developmental PCB exposure or the T4826I and DM genotypes were associated with decreased hippocampal soma area.

Examining PCB effects in each sex and genotype independently, PCB exposure altered soma area in WT, CGG, and DM males, as well as in WT and DM females. In WT males, soma area was significantly reduced in the 6 mg/kg PCB dose group compared to WT male vehicle controls. In CCG males, soma area was significantly reduced in the 1 and 6 mg/kg dose groups relative to CGG vehicle controls (Figure 3E). Soma area was also significantly reduced in 1 mg/kg DM males in contrast to DM vehicle control males (Figure 3E). While PCBs generally decreased soma area in males across genotypes, this effect was genotype-dependent in females. Like males, soma area of hippocampal neurons in WT females was significantly reduced in the 6 mg/kg dose group compared to WT female vehicle controls (Figure 3F). However, in DM females, soma area was significantly increased in the 6 mg/kg dose group vs. DM female vehicle controls (Figure 3F).

### Morphometric Effects of Polychlorinated Biphenyls and Genotypes on Layer IV/V Pyramidal Somatosensory Cortical Neurons

**FIGURE 4:**
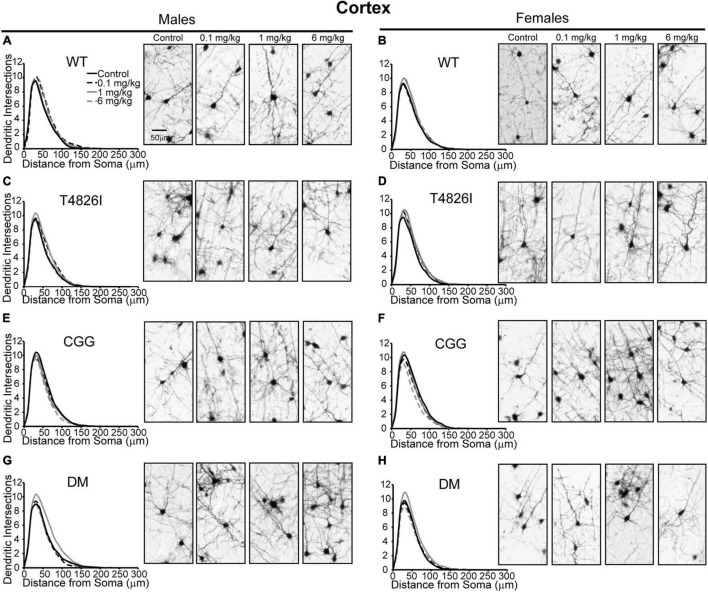
Genotype influences the effect of PCBs on the dendritic morphology of pyramidal neurons in the somatosensory cortex of juvenile mice. Sholl plots of the basilar dendritic arbors of Golgi-stained layer IV/V pyramidal somatosensory cortical neurons in P27-31 male and female **(A,B)** WT, **(C,D)** T4826I, **(E,F)** CGG, or **(G,H)** DM mice. Mice were exposed to the MARBLES PCB mix in the maternal diet throughout gestation and lactation. *N* = 44–48 neurons from at least six independent mice per sex per genotype per dose. See also Supplementary Figure 2 for representative tracings of the basilar arbors of Golgi-stained layer IV/V pyramidal somatosensory cortical neurons.

Polychlorinated biphenyl dose effects were more pronounced in cortical neurons compared to hippocampal neurons. Figure 4 illustrates Sholl plots and representative images of Golgi-stained pyramidal neurons in layer IV/V of the somatosensory cortical neurons from male and female WT, T4826I, CGG, and DM mice. There was a significant effect of dose on cortical Sholl profiles, with the 1 mg/kg PCB group exhibiting greater dendritic complexity than vehicle controls or the 6 mg/kg dose group (Table 2). There was also a significant genotype by dose interaction observed in the Sholl profiles (fully summarized in Table 2), identified as: (1) CGG vehicle control neurons were more complex than T4826I, DM, and WT vehicle control neurons; (2) 6 mg/kg CGG neurons were less complex than CGG vehicle control neurons; (3) 1 mg/kg T4826I neurons had greater complexity compared to T4826I and WT vehicle control neurons; and (4) 1 mg/kg DM neurons showed much greater complexity than DM vehicle controls, 0.1, or 6 mg/kg DM neurons, as well as increased complexity relative to WT vehicle controls, WT 1 mg/kg, or T4826I vehicle control groups. Distance from the soma of maximum dendritic intersections (Peak X) did not differ between dose groups, but there was a significant effect of dose on the maximum number of dendritic intersections (Peak Y), with the 1 mg/kg PCB dose group having increased intersections relative to all other dose groups (Table 2). There was a significant effect of dose on the total area under the Sholl curve, with 1 mg/kg PCB dose groups having increased area vs. vehicle controls or the 6 mg/kg PCB dose group as well as a significant genotype by dose effect driven by the same differences stated above for the Sholl profile analysis model (Table 2). For proximal area under the Sholl curve, there was a significant effect of dose with 1 mg/kg dose groups having greater area than all other dose groups, as well as a significant genotype by dose interaction driven by all differences listed above for the Sholl profile analysis with the addition of the DM 6 mg/kg PCB dose group having decreased proximal area compared to the WT 6 mg/kg PCB dose group (Table 2). In contrast, distal AUC showed fewer differences. CGG vehicle controls had greater distal AUC than WT, T4826I, or DM vehicle control neurons, and exposure to 6 mg/kg PCB decreased distal AUC in CGG neurons relative to CGG vehicle controls. The DM 1 mg/kg group showed greater distal AUC than DM vehicle controls, all other DM PCB dose groups, 1 mg/kg PCB WT mice, and vehicle controls from T4826I and WT mice (Table 2). Together, these results indicate a non-monotonic dose response, with exposure to the MARBLES PCB mix at 1 mg/kg promoting dendritic arborization, especially in T4826I and DM neurons. Vehicle-treated CGG animals have the greatest dendritic complexity compared to the other genotypes, and the highest PCB dose (6 mg/kg) decreased dendritic complexity within CGG neurons.

**FIGURE 5:**
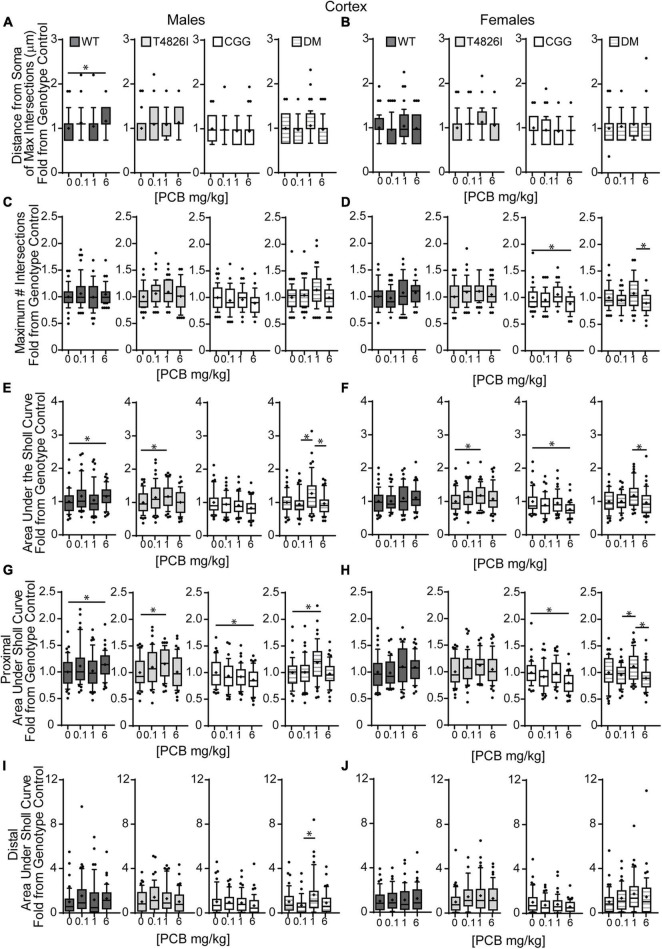
Effects of PCBs on the dendritic morphology of pyramidal neurons in the somatosensory cortex of juvenile mice vary between genotypes and are dose and sex dependent. Morphometric analyses of the basilar dendritic arbors of Golgi-stained pyramidal CA1 hippocampal neurons from P27-31 male and female WT, T4826I, CGG, or DM mice exposed to the MARBLES PCB mixture in the maternal diet throughout gestation and lactation. **(A,B)** The distance from the soma to the maximum number of dendritic intersections (Peak X). **(C,D)** The maximum number of dendritic intersections (Peak Y). **(E,F)** The total area under the curve of the Sholl plot (0–280 μm from the soma). **(G,H)** Proximal area under the curve of the Sholl plot (10–70 μm from the soma). **(I,J)** Distal area under the curve of the Sholl plot (80–140 μm from the soma). Data (*N* = 44–48 neurons from at least six independent mice per sex per genotype per dose) are presented as box plots, where the box indicates the lower (25th) to upper (75th) quartiles, the “+” indicates the mean, whiskers indicate the 10–90th percentile, and dots represent values outside the upper or lower fences. Bar and asterisk indicate a significant difference between groups as determined by Kruskal–Wallis test followed by Dunn’s multiple comparisons test, or (E-T4826I, G-CGG) one-way ANOVA followed by Dunnett’s multiple comparisons test or (H-DM) Tukey’s multiple comparisons test with *p* ≤ 0.05 considered statistically significant. Average values of vehicle controls for WT, T4826I, CGG and DM, respectively, are **(A)** 27.2, 27.1, 30.8, 30.2 μm; **(B)** 31.0, 27.7, 31.9, 27.1 μm; **(C)** 10.1, 9.9, 11.0, 9.6 intersections; **(D)** 10.0, 9.9, 10.9, 10.5 intersections; **(E)** 504.0, 502.9, 603.5, 534.2 μm^2^; **(F)** 522.7, 516.4, 642.1, 522.7 μm^2^; **(G)** 403.8, 396.4, 466.4, 405.5 μm^2^; **(H)** 407.9, 418.8, 477.8, 428.9 μm^2^; **(I)** 52.7, 58.5, 81.4, 74.4 μm^2^; and **(J)** 64.9, 52.8, 101.4, 47.2 μm^2^.

We next examined the effects of PCBs on dendritic growth when independently analyzed within sex and genotype (Figure 5). In WT mice, the distance from the soma of the maximum number of intersections (Peak X) was increased in the 6 mg/kg PCB dose group relative to vehicle controls in males, but not females (Figures 5A,B). PCB effects on the maximum number of dendritic intersections (Peak Y) were limited to female animals, where they were decreased in 6 mg/kg CGG neurons vs. CGG vehicle controls and in 6 mg/kg DM females relative to 1 mg/kg DM females (Figure 5D). PCB effects on the area under the Sholl curve were genotype- and sex-dependent. In WT animals, the total AUC was increased in the male 6 mg/kg dose group vs. WT male vehicle controls (Figure 5E). In T4826I animals, the total area under the Sholl curve was increased in the male 1 mg/kg dose group vs. T4826I male vehicle control (Figure 5E). Total area under the Sholl curve was also increased in DM males in the 1 mg/kg dose group vs. the 0.1 or 6 mg/kg dose group (Figure 5E). In contrast to males, total area under the Sholl curve was unchanged in WT female mice and was decreased in CGG 6 mg/kg females compared to CGG vehicle control females (Figure 5F). Like males, total area under the Sholl curve was greater in T4826I 1 mg/kg females compared to T4826I vehicle control females (Figure 5F) and total area under the Sholl curve was greater in DM 1 mg/kg females vs. the DM 6 mg/kg females (Figure 5F). For both males and females, most of the differences in AUC occurred in the proximal portion. Similar to total area under the Sholl curve, proximal area under the Sholl curve was increased in the 6 mg/kg dose group vs. vehicle control in WT males, and increased in the 1 mg/kg PCB dose group vs. vehicle control in T4826I and DM males (Figure 5G). In CGG males and females, proximal area under the Sholl curve was decreased in the 6 mg/kg PCB dose group vs. vehicle controls, compared to an increase in DM 1 mg/kg females compared to 0.1 or 6 mg/kg DM females (Figure 5H). The only significant difference in the distal area under the Sholl curve was found in DM males, with a significant increase in the 1 mg/kg PCB dose group relative to the 0.1 mg/kg group (Figures 5I,J). In summary, these results indicate that developmental exposure to the MARBLES PCB mixture increased dendritic complexity in WT male cortical neurons at 6 mg/kg, an effect that was influenced by genotype as T4826I and DM male cortical neurons had indices of increased dendritic complexity at the 1 mg/kg dose. In contrast, PCB exposure only affected female cortical neurons from T4826I and CGG genotypes when compared to vehicle controls, with increased complexity in T4826I cortical neurons at the 1 mg/kg dose but decreased complexity in CGG cortical neurons at the 6 mg/kg dose.

In other measures of dendritic arborization, effects were driven by PCB dose for the total number of basilar dendrites, terminal dendritic tips, dendritic length sum and the number of nodes, with the 1 mg/kg PCB dose groups having greater complexity than all other dose groups (Table 2). In addition, there was a significant genotype by dose interaction for total dendritic length, which was largely driven by differences highlighted above for the cortical Sholl profile analysis (Table 2). There was an increase in the number of dendritic tips per dendrite in the 1 mg/kg PCB dose vs. the 0.1 mg/kg or 6 mg/kg PCB dose groups; there were no effects of PCB exposure on mean dendritic length (Table 2). Together, these results indicate a non-monotonic dose response, with the 1 mg/kg PCB dose group having the greatest response and an overall tendency to increased dendritic complexity, especially in T4826I and DM neurons. In contrast, vehicle-treated CGG neurons were more complex than the other genotypes with the exception of the 6 mg/kg dose group, which exhibited decreased CGG neuron complexity.

**FIGURE 6:**
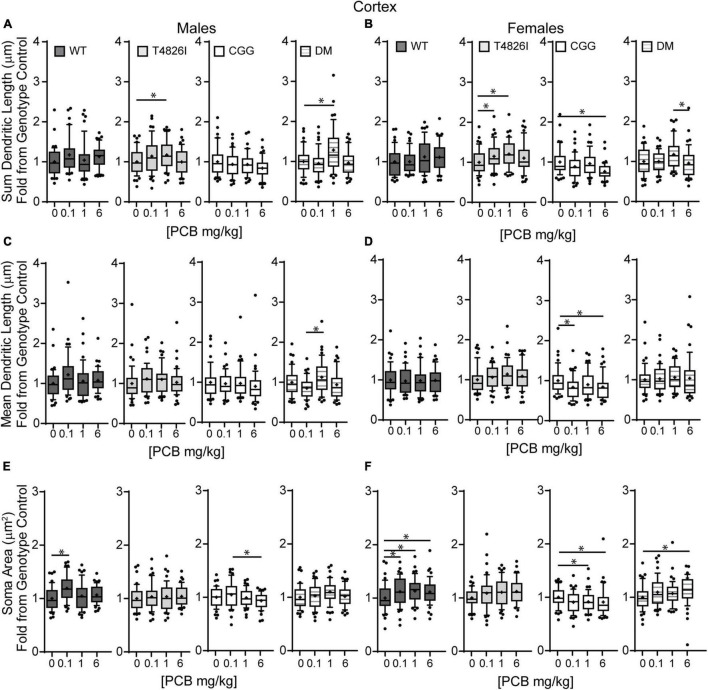
Polychlorinated biphenyls effects on dendritic length and soma area are more prevalent in female vs. male pyramidal somatosensory cortical neurons. Morphometric analyses of the basilar dendritic arbors of Golgi-stained pyramidal CA1 hippocampal neurons from P27–31 male and female WT, T4826I, CGG, or DM mice exposed to the MARBLES PCB mixture in the maternal diet throughout gestation and lactation. **(A,B)** Sum length of dendrites per neuron (μm). **(C,D)** Mean dendritic length per neuron (μm). **(E,F)** Cell body area (μm^2^). Data (*N* = 44–48 neurons from at least six independent mice per sex per genotype per dose) are presented as box plots, where the box indicates the lower (25th) to upper (75th) quartiles, the “+” indicates the mean, whiskers indicate the 10–90th percentile, and dots represent values outside the upper or lower fences. Bar and asterisk indicate a significant difference between groups as determined by Kruskal–Wallis test followed by Dunn’s multiple comparisons test, or (A-T4826I, E-WT, DM) one-way ANOVA followed by Dunnett’s multiple comparisons test or (E-CGG) one-way ANOVA with Welch’s correction followed by Dunnett’s T3 multiple comparison test with *p* ≤ 0.05 considered statistically significant. Average values of vehicle controls for WT, T4826I, CGG and DM, respectively, are **(A)** 582.2, 583.0, 699.4, 617.5 μm; **(B)** 603.1, 591.4, 740.5, 612.1 μm; **(C)** 94.7, 96.9, 113.8, 110.5 μm; **(D)** 108.6, 99.8, 123.7, 100.2 μm; **(E)** 229.2, 228.0, 256.1, 235.1 μm^2^; and **(F)** 233.2, 224.6, 267.3, 222.9 μm^2^.

Examining PCB dose effects in each sex and genotype independently, we observed differences in the number of primary dendrites were limited to CGG females, with the number of primary dendrites decreased in the 6 mg/kg dose group compared to the 1 mg/kg dose group (Supplementary Figure 4A,B). Effects of developmental PCB exposure on the number of dendritic tips were seen in offspring of both sexes, but to a greater extent in females. More specifically, the number of dendritic tips was increased in DM 1 mg/kg males vs. DM vehicle control males (Supplementary Figure 4C). However, in female neurons, the number of dendritic tips was increased in the T4826I 1 mg/kg dose group vs. vehicle control, decreased in the CGG 6 mg/kg dose group vs. vehicle control, and decreased in the DM 6 mg/kg dose group vs. the 1 mg/kg dose group (Supplementary Figure 4D). Dendritic tips in WT female neurons had a Kruskal–Wallis *p*-value of 0.05 however no differences compared to vehicle control were significantly different upon *post hoc* analysis (Supplementary Figure 4D). There were no differences in the number of dendritic tips normalized to primary dendrite number (Supplementary Figures 4E,F). Total dendritic length per neuron was increased in the 1 mg/kg dose group vs. vehicle control in T4826I and DM males and in the 1mg/kg and 0.1 mg/kg dose group in T4826I females (Figures 6A,B). Additionally, the total dendritic length was decreased in the female CGG 6 mg/kg dose group vs. vehicle control, and in the female DM 6 mg/kg dose group vs. 1 mg/kg dose group (Figure 6B). Mean dendritic length per neuron was increased in male DM in the 1 mg/kg PC dose group vs. the 0.1 mg/kg dose group (Figure 6C), but was decreased in CGG females in the 0.1 mg/kg and 6 mg/kg dose groups vs. vehicle control (Figure 6D). In summary, PCBs at 1 mg/kg tended to increase complexity within DM and T4826I males and T4826I females vs. vehicle control, and PCBs at 6 mg/kg decreased dendritic complexity in CGG females.

Similar to hippocampal neurons, soma area of cortical neurons was significantly impacted by genotype and PCB exposure. CGG neurons had greater soma area than T4826I and DM neurons and T4826I neurons had reduced soma area relative to WT neurons (Table 2). There was also a dose effect for soma area, with the 0.1 mg/kg and 1 mg/kg PCB dose groups having greater area than vehicle control neurons (Table 2). Examining each sex and genotype independently, soma area was increased in the 0.1 mg/kg PCB dose group vs. vehicle control in WT male neurons (Figure 6E). There was also a decrease in soma area in the 6 mg/kg PCB dose group vs. the 0.1 mg/kg PCB dose group in male CGG neurons (Figure 6E). A greater number of PCB effects were observed in female neurons. Unlike WT hippocampal neurons, there was a significant increase in soma area in the 0.1, 1, and 6 mg/kg PCB dose groups vs. vehicle control in WT female neurons (Figure 6F). In CGG female neurons, there was a significant decrease in soma area in the 1mg/kg and 6 mg/kg dose group compared to vehicle control. In contrast, in DM female neurons, there was a significant increase in soma area in the 6 mg/kg PCB dose group vs. vehicle control (Figure 6F). In summary, PCBs increased soma area in WT cortical neurons in a sex- and dose-dependent manner, and this effect was affected by genotype since CGG female neurons had decreased soma area while DM female neurons had increased soma area at the 6 mg/kg PCB dose group relative to vehicle controls.

Table 3 summarizes data presented in Figures 2, 3, 5, 6 and Supplementary Figures 3, 4, which are the PCB dose responses (indicated by arrows) within each sex and genotype relative to the vehicle control for each parameter of dendritic arborization that was measured in this study.

**TABLE 3:** Summary of PCB effects on neuron morphology within each sex and genotype.

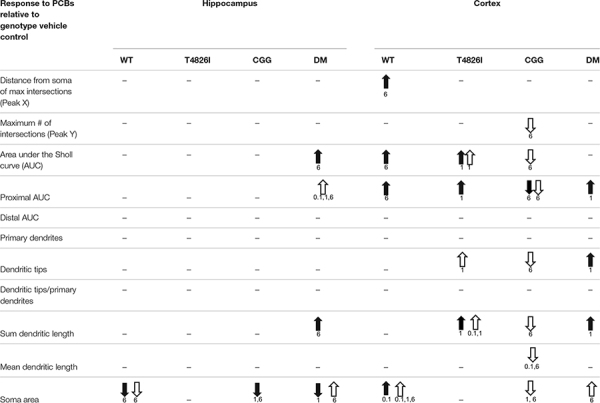

*Black Arrow: Male, White Arrow: Female, Dash indicates no effect.*

## Discussion

We describe novel data demonstrating that developmental exposure to a human-relevant PCB mixture alters dendritic arborization in the juvenile mouse brain; however, the dendritic outcome and dose-response relationship varied depending on sex, genotype, and brain region. These findings support the hypothesis that PCBs interact with heritable human mutations that alter the fidelity of neuronal Ca^2+^ signaling to confer NDD risk. This conclusion is based on two lines of evidence. First, dendritic arborization was significantly increased in cortical neurons of WT males in the 6 mg/kg PCB dose group. By comparison, the dendritic complexity of cortical neurons was significantly increased in T4826I and DM males in the 1 mg/kg PCB dose group, suggesting that expression of the T4826I-*RYR1* mutation, either alone or in combination with CGG mutation, increased sensitivity of male cortical neurons to the dendrite-promoting effects of the MARBLES PCB mixture, evident as a leftward shift of the dose-response relationship. This is consistent with previous reports that *RYR1* gain-of-function mutations confer heightened sensitivity to RyR-active PCBs *in vitro* (Ta and Pessah, 2007). Second, while developmental exposure of WT mice to the MARBLES PCB mixture had no significant effect on the dendritic morphology of male or female hippocampal neurons or female cortical neurons, it significantly altered the dendritic arborization of these neuronal cell types in mice that expressed one or more transgenes. Specifically, dendritic arborization of hippocampal neurons was significantly increased in DM males in the 6 mg/kg dose group and DM females in the 0.1, 1, and 6 mg/kg dose groups. The dendritic arbors of cortical neurons were more complex in T4826I females in the 1 mg/kg dose group, while dendritic arborization was decreased in CGG females in the 6 mg/kg dose group. Overall, these results add to a growing body of literature indicating that the genetic substrate can modulate the response to neurotoxic environmental chemicals.

Several interesting observations emerged from this study, including: (1) cortical neurons were more sensitive than hippocampal neurons to the dendritic effects of the MARBLES PCB mix; and (2) sex strongly influenced dendritic responses to PCB exposure. The observation of the differential sensitivity of cortical and hippocampal neurons is consistent with our earlier studies of dendritic arborization in the hippocampus and cortex of juvenile rats developmentally exposed to the commercial PCB mixture Aroclor 1254 (Lein et al., 2007; Yang et al., 2009). The observation regarding the influence of sex is also consistent with previous studies in which we demonstrated sex-dependent effects of PCB 95 and PCB 11 on the dendritic arborization of primary hippocampal and cortical neurons *in vitro* (Sethi et al., 2017; Keil et al., 2019a). The *in vivo* sex differences we observed in this study varied between genotypes. Specifically, PCB effects on dendritic arborization of cortical neurons were male-specific in WT and DM mice, but female-specific in CGG mice. Moreover, the direction of the dendritic response of cortical neurons to PCBs varied depending on sex, with male WT and DM cortical neurons exhibiting increased dendritic arborization and female CGG cortical neurons exhibiting decreased dendritic arborization. More subtle sex differences were observed in DM hippocampal neurons and T4826I cortical neurons: (1) female and male DM hippocampal neurons responded similarly to PCBs with increased dendritic arborization, but female neurons were more sensitive, responding to the MARBLES PCB mixtures at 0.1, 1, and 6 mg/kg/d while male neurons were affected only by the 6 mg/kg/d dose; and (2) while the direction of the dendritic response in DM hippocampal neurons and T4826I cortical neurons was similar between sexes, the specific parameters of dendritic arborization that were altered by PCBs differed. While it is widely posited that sex differences in dendritic arborization and neuronal connectivity contribute to the sex bias in the prevalence of a number of NDDs (Alaerts et al., 2016; McCarthy, 2016), our findings support an emerging literature suggesting that sex differences in the response to environmental neurotoxicant exposure may contribute to NDD sex bias.

The biological basis for the differential susceptibility of females *vs*. males and hippocampal *vs*. cortical neurons is not known. One possibility is sex and regional differences in PCB toxicokinetics. PCBs tend to be lipophilic and thus would be predicted to be uniformly distributed throughout the brain in both sexes; however, this has yet to be demonstrated. Moreover, it is now appreciated that hydroxylated metabolites of PCBs can have neurotoxic properties that differ from those of the parent congener (Klocke and Lein, 2020), and expression of the cytochrome P450 enzymes that metabolize PCBs differ by sex and brain region (Stamou et al., 2014). Another non-mutually exclusive possibility is that PCB toxicodynamics vary according to sex and/or brain region. While addressing this possibility will require identification of the mechanism(s) that mediate the effects of the MARBLES PCB mixture on dendritic arborization, if RyR activity is involved, there is significant evidence in the literature that expression of RyRs and accessory proteins that regulate its gating properties are developmentally regulated and vary across brain regions (Pessah et al., 2010).

A novel observation of this study was the effect of the MARBLES PCB mixture on soma size, with PCB effects on this morphometric parameter observed in all but the T4826I genotype. Generally, developmental PCB exposure decreased hippocampal soma size but increased cortical soma size. The two exceptions to this generalization were increased soma size of female DM hippocampal neurons in the 6 mg/kg dose group and decreased soma size of female CGG cortical neurons in the 1 mg/kg and 6 mg/kg dose group. Interestingly, PCB effects on soma size in hippocampal neurons were phenocopied in the T4826I and DM genotypes, which had hippocampal neurons with smaller soma sizes relative to WT controls. PCB effects on soma size did not necessarily correlate with PCB effects on dendritic complexity. For example, while developmental exposure to the MARBLES PCB mix significantly decreased soma size of hippocampal neurons in male and female WT mice, male CGG mice, and male DM mice, dendritic arborization in these neuronal cell types was either unaffected (male and female WT mice and male CGG mice) or increased (male DM mice) relative to sex- and genotype- matched control. Moreover, PCBs significantly increased dendritic arborization of male and female T4826I cortical neurons, but had no significant effect on soma size in these neurons. These observations suggest that different mechanisms mediate the morphometric effects of PCBs on soma vs. dendrites, and that the PCB effects on either morphometric parameter do not simply reflect general cellular hypertrophy.

Other environmental exposures have been reported to alter soma size. For example, developmental exposure to morphine was found to decrease or increase soma size of ventral tegmental area dopaminergic neurons depending on the brain region to which the neurons projected (Simmons et al., 2019). Soma size has been linked to cognitive ability, with increased hippocampal soma size in birds hypothesized to enhance spatial memory and survival in changing climate conditions (Freas et al., 2013). In a rat model of autism-like behavior, soma size of hippocampal CA1 pyramidal neurons was reduced in offspring developmentally exposed to valproic acid (Hajisoltani et al., 2019). The effects of reduced soma size on cognitive behavior may extend to humans, as hippocampal soma size is reduced in individuals with schizophrenia (Benes et al., 1991). Human iPSC cells with a knockdown of SHANK3, an autism-related gene, or neurons derived from iPSC from patients with Rett syndrome also exhibited reduced soma size (Marchetto et al., 2010; Huang et al., 2019). Conversely, there is evidence that increased soma size is associated with altered cognitive ability: mice lacking the FMR protein had increased neuronal somata (Selby et al., 2007). These observations suggest that either abnormally enlarged or reduced neuronal soma size may be detrimental to cognitive function, identifying another NDD-relevant outcome influenced by interactions between PCBs and human mutations associated with altered Ca^2+^-dependent signaling and/or neuronal connectivity.

A question raised by this study is whether gene dosage affected dendritic arborization in the absence or presence of developmental PCB exposures. Gene dosage seemed to influence dendritic outcome independent of developmental PCB exposure as evidenced by the observation that male DM hippocampal neurons had significantly more complex dendritic arbors than male T4826I hippocampal neurons (assessed as distal area under the Sholl curve, Table 1). Gene dosage also seemed to influence sensitivity of hippocampal neurons to the dendritic effects of the MARBLES PCB mixture since PCB effects on this neuronal cell type were only observed in male and female DM mice. Assessing the influence of gene dosage on the response of cortical neurons to PCBs is more difficult because PCB effects on cortical neurons were more complex. Nonetheless, male DM cortical neurons were more sensitive to the dendrite-promoting activity of PCBs than male WT and CCG neurons. Conversely, the dendritic arborization of female DM cortical neurons was not altered by developmental PCB exposure compared to vehicle control, while female T4826I cortical neurons responded to PCBs with more complex dendritic arbors and female CGG cortical neurons responded with less complex dendritic arbors. Based on these observations, it is difficult to determine whether the T4826I and CGG genotypes contributed equally to the DM phenotype. In male cortical neurons, developmental PCB exposure increased the proximal area under the Sholl curve in both T4826I and DM mice in the 1 mg/kg dose group, but had no effect or reduced this parameter in male CGG cortical neurons, suggesting this phenotype in DM males was driven largely by the T4826I-*RYR1* mutation. However, in male hippocampal neurons, developmental PCB exposure decreased soma area in CGG and DM neurons of mice in the 1 mg/kg dose group but not in T4826I neurons, suggesting this PCB response is largely influenced by the CGG mutation. Yet in other cases, the DM response to PCBs was not phenocopied by either the T4826I or CGG genotype. For example, in hippocampal neurons, PCB responses were only seen in DM mice and not mice of the other genotypes. Additionally, in female cortical neurons, developmental PCB exposure increased dendritic complexity in T4826I mice in the 1mg/kg dose group, decreased dendritic arborization in CGG mice in the 6 mg/kg dose group, and had no effect on dendritic arborization in DM mice compared to vehicle controls. This latter scenario may reflect an additive effect of both genotypes. Collectively, these observations suggest that while the T4826I-*RYR1* and CGG mutations both alter the fidelity of Ca^2+^ signaling in neurons (Barrientos et al., 2012; Cao et al., 2012; Robin et al., 2017), the interactions between these mutations in the DM mice are complex, potentially reflecting mechanism(s) independent of Ca^2+^ signaling.

Several potential mechanisms by which MARBLES PCBs interact with the T4826I-*RYR1* and *FMR1* CGG repeat expansion mutations to modulate dendritic arborization include (1) PCB induced changes in the expression of RYR1 and FMR1/FMRP and/or (2) convergence on the same signaling systems dysregulated by these genetic factors at critical times during development. With respect to the former, we have previously demonstrated that gestational and lactational exposure to Aroclor 1254 in the maternal diet at 1 or 6 mg/kg/d dose-dependently increased RyR expression in the cerebellum of weanling pups (Yang et al., 2009). Whether the MARBLES mix similarly increases RyR expression and whether any PCB(s) increase expression of FMRP is not known, but should be the focus of future investigations. Several lines of evidence support a model in which PCBs and genetic factors converse on Ca^2+^-dependent signaling pathways. First, the MARBLES PCB mixture has RyR activity as determined by equilibrium binding of [^3^H]ryanodine to RyR1-enriched microsomes (Sethi et al., 2019). Moreover, two of the MARBLES PCB congeners, PCB 95 and PCB 11, promote dendritic growth in primary hippocampal and cortical neurons via activation of Ca^2+^-dependent signaling pathways involving CREB, Wnt, miR132, and/or mTOR (Yang et al., 2009; Wayman et al., 2012a,b; Lesiak et al., 2014; Keil et al., 2018; Sethi et al., 2018). The signaling pathways activated by PCBs to increase dendritic arborization map onto Ca^2+^-dependent signaling pathways altered in NDDs (Stamou et al., 2013; Panesar et al., 2020). Second, both the T4826I-*RYR1* gain of function mutation (Barrientos et al., 2012) and the *FMR1* CGG repeat expansion mutation (Cao et al., 2012; Robin et al., 2017) have been shown to increase resting intracellular Ca^2+^ concentrations and spontaneous Ca^2+^ oscillations in neuronal cells. Increased intracellular Ca^2+^ promotes dendritic growth via a CaMK-CREB-Wnt signaling pathway (Wayman et al., 2006) and dendritic spine formation via a CREB-miR132 pathway (Impey et al., 2010). Intracellular Ca^2+^ also regulates mTOR-dependent translational control of dendritic growth (Kumar et al., 2005; Urbanska et al., 2012). The *FMR1* CGG repeat expansion mutation results in decreased expression of the translational repressor FMRP (Hagerman and Hagerman, 2013), which effectively alters mTOR signaling (Wang et al., 2012). FMRP also functions as a chaperone for miR132, but the effects of decreased FMRP on miR132 signaling are not known. Expression of either the *RYR1* gain of function mutation (Pessah, personal communication) or the *FMR1* CGG repeat expansion (Chen et al., 2010) have been shown to alter dendritic growth in primary neurons. We propose that at least a subset of PCB congeners in the MARBLES PCB mixture converge on these signaling pathways to amplify the effects of these gene mutations on dendritic arborization.

While further studies are required to confirm this model, it does provide a potential explanation for the observation that in contrast to previous studies of rats exposed developmentally to PCB 95 (Wayman et al., 2012b) or Aroclor 1254 (Lein et al., 2007; Yang et al., 2009), the MARBLES mix did not promote dendritic arborization in the CA1 pyramidal neurons in the hippocampus of WT mice. PCB 95 is among the most potent congener with respect to RyR sensitization (Pessah et al., 2010), and Aroclor 1254 contains a significant percentage of RyR-active PCB congeners, including PCB 95 (Howard et al., 2003). In contrast, PCB 95 comprised only 1.2% of the total mass in the MARBLES mixture. Moreover, a comparative analysis of the *in vitro* RyR potency of PCB 95 *vs*. the MARBLES mix showed that the MARBLES mix activates the RyR at micromolar concentrations with a maximal activation of 4-fold while PCB 95 activated the RyR at nanomolar concentrations and the maximal activation was 12-fold (Sethi et al., 2019). This earlier *in vitro* study compared the RyR potency of each of the individual PCB congeners in the MARBLES mix, and the results indicate that the most potent RyR sensitizing congeners comprise ∼9% of the MARBLES mix. Therefore, if RyR sensitization is the predominant mechanism driving PCB-induced dendritic arborization, it is perhaps not surprising that the MARBLES mix did not promote dendritic arborization in hippocampal neurons of WT animals. However, we did observe increased dendritic arborization of cortical neurons in WT animals, suggesting that the dose-dependency of PCB-induced dendritic growth varies between brain regions.

A model in which PCBs and genetic factors interact via convergence on Ca^2+^-dependent signaling pathways also provides a potential explanation for the non-monotonic dose-related effect of the MARBLES PCB mix on dendritic arborization. Multilevel linear mixed-effects modeling identified a main effect of dose on the dendritic complexity of cortical neurons with the 1 mg/kg PCB dose group exhibiting significantly increased dendritic arborization compared to vehicle controls or the 0.1 and 6 mg/kg dose groups. A similar non-monotonic dose-response relationship has been reported in previous *in vivo* and *in vitro* studies of Aroclor 1254, PCB 95 and PCB 136 (Yang et al., 2009, 2014; Wayman et al., 2012b). The mechanism underlying this dose-response relationship is not known, but a possibility is suggested by *in vitro* studies demonstrating that moderate increases in Ca^2+^ promote dendritic growth whereas large increases cause dendritic retraction (Segal et al., 2000; Lohmann and Wong, 2005). Thus, if PCBs and the T4826I-*RYR1* and *FMR1* CGG repeat expansion mutations are modulating dendritic growth via increased levels of intracellular Ca^2+^, then higher PCB doses, increased gene dosage, or the combination of PCBs and gene mutations may increase intracellular Ca^2+^ above concentrations that promote dendritic growth to levels that trigger dendritic retraction, perhaps via activation of calpain (Baudry et al., 2013) or preferential activation of CaMKIV (Redmond et al., 2002). This mechanism may also explain the observation that MARBLES PCBs decreased dendritic complexity of female CGG cortical neurons. Testing this hypothesis is an important area of future study, findings from which will expand our understanding of how environmental and genetic risk factors and potentially provide algorithms for predicting specific gene-environment interactions likely to increase the risk of adverse neurodevelopmental outcomes.

An outstanding question is whether effects of the MARBLES PCB mixture on dendritic arborization are linked to changes in behavior. The animals used in this study were assessed in tasks that measured social communication, repetitive behavior and sociability (data under review). While aberrant behavior was observed in PCB-exposed animals, there was not a one-to-one correlation between PCB effects on dendritic growth and behavior in terms of dose-response relationships or genotype effects. However, this does not negate the relevance of the dendritic findings since we may not have captured the neuroanatomic circuits that mediate the behaviors that were assessed. We believe the dendritic findings are relevant to human NDDs for several reasons. First, animals were exposed to a human-relevant PCB mixture that reflected the PCB congener profile in the gestational environment of at-risk individuals, and the PCB concentrations measured in brain tissue of exposed pups were within the range of PCB levels measured in human brain tissue (Sethi et al., under review)^2^. Second, both increased and decreased dendritic arborization are thought to contribute to the clinical phenotypes associated with many NDDs (Coskun et al., 2013; Keown et al., 2013; Khan et al., 2015; Alaerts et al., 2016; Cooper et al., 2017). In summary, these studies add to the growing body of literature implicating PCBs as NDD risk factors, and identify genetic mutations that may amplify the effects of neurotoxic PCBs on the developing brain.

## Data Availability Statement

The raw data supporting the conclusions of this article will be made available by the authors, without undue reservation.

## Ethics Statement

The animal study was reviewed and approved by University of California, Davis Institutional Animal Care and Use Committee.

## Author Contributions

IP and PL conceptualized the project and obtained funding to support the work. PL supervised all aspects of this study. KK, SS, and PL designed the experiments. KK and SS maintained the mouse colony, dosed the animals, collected tissues for PCB quantitation, and conducted the statistical analysis of the independent PCB dose effects. KK, SS, TR, and CK conducted the Golgi analysis. MW conducted the mixed-effects modeling of the morphometric data. KK, SS, and CK composed the figures. KK drafted the initial manuscript. CK and PL made significant edits to the early versions of the manuscript. All authors listed have made a substantial, direct, and intellectual contribution to the work and approved it for publication.

## Conflict of Interest

The authors declare that the research was conducted in the absence of any commercial or financial relationships that could be construed as a potential conflict of interest.

## Publisher’s Note

All claims expressed in this article are solely those of the authors and do not necessarily represent those of their affiliated organizations, or those of the publisher, the editors and the reviewers. Any product that may be evaluated in this article, or claim that may be made by its manufacturer, is not guaranteed or endorsed by the publisher.

## Footnotes

1
www.neuromorpho.org

2 Sethi, S., Keil Stietz, K. P., Valenzuela, A. E., Klocke, C. R., Silverman, J. L., Puschner, B., et al. (under review). Developmental exposure to a human-relevant PCB mixture causes behavioral phenotypes that vary by sex and genotype in juvenile mice expressing human mutations that modulate neuronal calcium. *Front. Neurosci.*

## References

[B1] Alaerts K. Swinnen S. P. Wenderoth N. (2016). Sex differences in autism: a resting-state fMRI investigation of functional brain connectivity in males and females. *Soc. Cogn. Affect. Neurosci.* 11 1002–1016. 10.1093/scan/nsw027 26989195 PMC4884321

[B2] Barrientos G. C. Feng W. Truong K. Matthaei K. I. Yang T. Allen P. D. (2012). Gene dose influences cellular and calcium channel dysregulation in heterozygous and homozygous T4826I-RYR1 malignant hyperthermia-susceptible muscle. *J. Biol. Chem.* 287 2863–2876. 10.1074/jbc.M111.307926 22139840 PMC3267780

[B3] Baudry M. Chou M. M. Bi X. (2013). Targeting calpain in synaptic plasticity. *Expert Opin. Ther. Targets* 17 579–592. 10.1517/14728222.2013.766169 23379852 PMC4154356

[B4] Benes F. M. Sorensen I. Bird E. D. (1991). Reduced neuronal size in posterior hippocampus of schizophrenic patients. *Schizophr. Bull.* 17 597–608. 10.1093/schbul/17.4.597 1805353

[B5] Berghuis S. A. Bos A. F. Sauer P. J. Roze E. (2015). Developmental neurotoxicity of persistent organic pollutants: an update on childhood outcome. *Arch. Toxicol.* 89 687–709. 10.1007/s00204-015-1463-3 25618547

[B6] Berman R. F. Murray K. D. Arque G. Hunsaker M. R. Wenzel H. J. (2012). Abnormal dendrite and spine morphology in primary visual cortex in the CGG knock-in mouse model of the fragile X premutation. *Epilepsia* 53 150–160. 10.1111/j.1528-1167.2012.03486.x 22612820 PMC4316681

[B7] Cao Z. Hulsizer S. Tassone F. Tang H. T. Hagerman R. J. Rogawski M. A. (2012). Clustered burst firing in FMR1 premutation hippocampal neurons: amelioration with allopregnanolone. *Hum. Mol. Genet.* 21 2923–2935. 10.1093/hmg/dds118 22466801 PMC3373240

[B8] Chen J. L. Nedivi E. (2010). Neuronal structural remodeling: is it all about access? *Curr. Opin. Neurobiol.* 20 557–562. 10.1016/j.conb.2010.06.002 20621466 PMC2946477

[B9] Chen Y. Tassone F. Berman R. F. Hagerman P. J. Hagerman R. J. Willemsen R. (2010). Murine hippocampal neurons expressing Fmr1 gene premutations show early developmental deficits and late degeneration. *Hum. Mol. Genet.* 19 196–208. 10.1093/hmg/ddp479 19846466 PMC2792156

[B10] Chonchaiya W. Au J. Schneider A. Hessl D. Harris S. W. Laird M. (2012). Increased prevalence of seizures in boys who were probands with the FMR1 premutation and co-morbid autism spectrum disorder. *Hum. Genet.* 131 581–589. 10.1007/s00439-011-1106-6 22001913 PMC4105134

[B11] Cline H. T. (2001). Dendritic arbor development and synaptogenesis. *Curr. Opin. Neurobiol.* 11 118–126. 10.1016/s0959-4388(00)00182-3 11179881

[B12] Cooper R. A. Richter F. R. Bays P. M. Plaisted-Grant K. C. Baron-Cohen S. Simons J. S. (2017). Reduced hippocampal functional connectivity during episodic memory retrieval in autism. *Cereb. Cortex* 27 888–902. 10.1093/cercor/bhw417 28057726 PMC5390398

[B13] Coskun M. A. Loveland K. A. Pearson D. A. Papanicolaou A. C. Sheth B. R. (2013). Functional assays of local connectivity in the somatosensory cortex of individuals with autism. *Autism Res.* 6 190–200. 10.1002/aur.1276 23427110 PMC3661715

[B14] Fernandez E. Rajan N. Bagni C. (2013). The FMRP regulon: from targets to disease convergence. *Front. Neurosci.* 7:191. 10.3389/fnins.2013.00191 24167470 PMC3807044

[B15] Freas C. A. Roth T. C. LaDage L. D. Pravosudov V. V. (2013). Hippocampal neuron soma size is associated with population differences in winter climate severity in food-caching chickadees. *Funct. Ecol.* 27 1341–1349. 10.1111/1365-2435.12125

[B16] Granillo L. Sethi S. Keil K. P. Lin Y. Ozonoff S. Iosif A. M. (2019). Polychlorinated biphenyls influence on autism spectrum disorder risk in the MARBLES cohort. *Environ. Res.* 171 177–184. 10.1016/j.envres.2018.12.061 30665119 PMC6382542

[B17] Grove J. Ripke S. Als T. D. Mattheisen M. Walters R. K. Won H. (2019). Identification of common genetic risk variants for autism spectrum disorder. *Nat. Genet.* 51 431–444. 10.1038/s41588-019-0344-8 30804558 PMC6454898

[B18] Hagerman R. Hagerman P. (2013). Advances in clinical and molecular understanding of the FMR1 premutation and fragile X-associated tremor/ataxia syndrome. *Lancet Neurol.* 12 786–798. 10.1016/S1474-4422(13)70125-X 23867198 PMC3922535

[B19] Hajisoltani R. Karimi S. A. Rahdar M. Davoudi S. Borjkhani M. Hosseinmardi N. (2019). Hyperexcitability of hippocampal CA1 pyramidal neurons in male offspring of a rat model of autism spectrum disorder (ASD) induced by prenatal exposure to valproic acid: a possible involvement of Ih channel current. *Brain Res.* 1708 188–199. 10.1016/j.brainres.2018.12.011 30537517

[B20] Hertz-Picciotto I. Schmidt R. J. Walker C. K. Bennett D. H. Oliver M. Shedd-Wise K. M. (2018). A prospective study of environmental exposures and early biomarkers in autism spectrum disorder: design, protocols, and preliminary data from the MARBLES study. *Environ. Health Perspect.* 126:117004. 10.1289/EHP535 30465702 PMC6371714

[B21] Howard A. S. Fitzpatrick R. Pessah I. Kostyniak P. Lein P. J. (2003). Polychlorinated biphenyls induce caspase-dependent cell death in cultured embryonic rat hippocampal but not cortical neurons *via* activation of the ryanodine receptor. *Toxicol. Appl. Pharmacol.* 190 72–86. 10.1016/s0041-008x(03)00156-x 12831785

[B22] Huang G. Chen S. Chen X. Zheng J. Xu Z. Doostparast Torshizi A. (2019). Uncovering the functional link between SHANK3 deletions and deficiency in neurodevelopment Using iPSC-derived human neurons. *Front. Neuroanat.* 13:23. 10.3389/fnana.2019.00023 30918484 PMC6424902

[B23] Impey S. Davare M. Lasiek A. Fortin D. Ando H. Varlamova O. (2010). An activity-induced microRNA controls dendritic spine formation by regulating Rac1-PAK signaling. *Mol. Cell Neurosci.* 43 146–156. 10.1016/j.mcn.2009.10.005 19850129 PMC2818337

[B24] Keil K. P. Miller G. W. Chen H. Sethi S. Schmuck M. R. Dhakal K. (2018). PCB 95 promotes dendritic growth in primary rat hippocampal neurons *via* mTOR-dependent mechanisms. *Arch. Toxicol.* 92 3163–3173. 10.1007/s00204-018-2285-x 30132043 PMC6162988

[B25] Keil K. P. Sethi S. Wilson M. D. Silverman J. L. I Pessah N. Lein P. J. (2019b). Genetic mutations in Ca^2+^ signaling alter dendrite morphology and social approach in juvenile mice. *Genes Brain Behav.* 18:e12526. 10.1111/gbb.12526 30311737 PMC6540090

[B26] Keil K. P. Sethi S. Lein P. J. (2019a). Sex-Dependent Effects of 2,2’,3,5’,6-Pentachlorobiphenyl on Dendritic Arborization of Primary Mouse Neurons. *Toxicol. Sci.* 168 95–109. 10.1093/toxsci/kfy277 30395321 PMC6390665

[B27] Keil K. P. Sethi S. Wilson M. D. Chen H. Lein P. J. (2017). *In vivo* and *in vitro* sex differences in the dendritic morphology of developing murine hippocampal and cortical neurons. *Sci. Rep.* 7:8486. 10.1038/s41598-017-08459-z 28814778 PMC5559594

[B28] Keown C. L. Shih P. Nair A. Peterson N. Mulvey M. E. Muller R. A. (2013). Local functional overconnectivity in posterior brain regions is associated with symptom severity in autism spectrum disorders. *Cell Rep.* 5 567–572. 10.1016/j.celrep.2013.10.003 24210815 PMC5708538

[B29] Khan S. Michmizos K. Tommerdahl M. Ganesan S. Kitzbichler M. G. Zetino M. (2015). Somatosensory cortex functional connectivity abnormalities in autism show opposite trends, depending on direction and spatial scale. *Brain* 138 1394–1409. 10.1093/brain/awv043 25765326 PMC5013931

[B30] Kilkenny C. Browne W. J. I Cuthill C. Emerson M. Altman D. G. (2010). Improving bioscience research reporting: the ARRIVE guidelines for reporting animal research. *PLoS Biol.* 8:e1000412. 10.1371/journal.pbio.1000412 20613859 PMC2893951

[B31] Kim J. H. Jarvik G. P. Browning B. L. Rajagopalan R. Gordon A. S. Rieder M. J. (2013). Exome sequencing reveals novel rare variants in the ryanodine receptor and calcium channel genes in malignant hyperthermia families. *Anesthesiology* 119 1054–1065. 10.1097/ALN.0b013e3182a8a998 24013571 PMC4115638

[B32] Klocke C. Lein P. J. (2020). Evidence Implicating Non-Dioxin-Like Congeners as the Key Mediators of Polychlorinated Biphenyl (PCB) Developmental Neurotoxicity. *Int. J. Mol. Sci.* 21:1013. 10.3390/ijms21031013 32033061 PMC7037228

[B33] Koh W. X. Hornbuckle K. C. Thorne P. S. (2015). Human Serum from Urban and Rural Adolescents and Their Mothers Shows Exposure to Polychlorinated Biphenyls Not Found in Commercial Mixtures. *Environ. Sci. Technol.* 49 8105–8112. 10.1021/acs.est.5b01854 26053216 PMC4774248

[B34] Konur S. Ghosh A. (2005). Calcium signaling and the control of dendritic development. *Neuron* 46 401–405. 10.1016/j.neuron.2005.04.022 15882639

[B35] Krey J. F. Dolmetsch R. E. (2007). Molecular mechanisms of autism: a possible role for Ca2+ signaling. *Curr. Opin. Neurobiol.* 17 112–119. 10.1016/j.conb.2007.01.010 17275285

[B36] Krueger D. D. Bear M. F. (2011). Toward fulfilling the promise of molecular medicine in fragile X syndrome. *Annu. Rev. Med.* 62 411–429. 10.1146/annurev-med-061109-134644 21090964 PMC3100156

[B37] Kumar V. Zhang M. X. Swank M. W. Kunz J. Wu G. Y. (2005). Regulation of dendritic morphogenesis by Ras-PI3K-Akt-mTOR and Ras-MAPK signaling pathways. *J. Neurosci.* 25 11288–11299. 10.1523/JNEUROSCI.2284-05.2005 16339024 PMC6725910

[B38] Leehey M. A. Hagerman P. J. (2012). Fragile X-associated tremor/ataxia syndrome. *Handb. Clin. Neurol.* 103 373–386. 10.1016/B978-0-444-51892-7.00023-1 21827901

[B39] Lein P. J. Yang D. Bachstetter A. D. Tilson H. A. Harry G. J. Mervis R. F. (2007). Ontogenetic alterations in molecular and structural correlates of dendritic growth after developmental exposure to polychlorinated biphenyls. *Environ. Health Perspect.* 115 556–563. 10.1289/ehp.9773 17450224 PMC1852648

[B40] Lesiak A. Zhu M. Chen H. Appleyard S. M. Impey S. Lein P. J. (2014). The environmental neurotoxicant PCB 95 promotes synaptogenesis *via* ryanodine receptor-dependent miR132 upregulation. *J. Neurosci.* 34 717–725. 10.1523/JNEUROSCI.2884-13.2014 24431430 PMC3891953

[B41] Li X. Holland E. B. Feng W. Zheng J. Dong Y. I Pessah N. (2018). Authentication of synthetic environmental contaminants and their (bio)transformation products in toxicology: polychlorinated biphenyls as an example. *Environ. Sci. Pollut. Res. Int.* 25 16508–16521. 10.1007/s11356-017-1162-0 29322390 PMC6015536

[B42] Liu J. Koscielska K. A. Cao Z. Hulsizer S. Grace N. Mitchell G. (2012). Signaling defects in iPSC-derived fragile X premutation neurons. *Hum. Mol. Genet.* 21 3795–3805. 10.1093/hmg/dds207 22641815 PMC3412379

[B43] Lohmann C. Wong R. O. (2005). Regulation of dendritic growth and plasticity by local and global calcium dynamics. *Cell Calcium* 37 403–409. 10.1016/j.ceca.2005.01.008 15820387

[B44] Lu A. T. Cantor R. M. (2012). Allowing for sex differences increases power in a GWAS of multiplex Autism families. *Mol. Psychiatry* 17 215–222. 10.1038/mp.2010.127 21151189

[B45] Lyall K. Croen L. A. Sjodin A. Yoshida C. K. Zerbo O. Kharrazi M. (2017). Polychlorinated biphenyl and organochlorine pesticide concentrations in maternal mid-pregnancy serum samples: association with autism spectrum disorder and intellectual disability. *Environ. Health Perspect.* 125 474–480. 10.1289/EHP277 27548254 PMC5332182

[B46] Marchetto M. C. Carromeu C. Acab A. Yu D. Yeo G. W. Mu Y. (2010). A model for neural development and treatment of Rett syndrome using human induced pluripotent stem cells. *Cell* 143 527–539. 10.1016/j.cell.2010.10.016 21074045 PMC3003590

[B47] Matelski L. Keil Stietz K. P. Sethi S. Taylor S. L. Van de Water J. Lein P. J. (2020). The influence of sex, genotype, and dose on serum and hippocampal cytokine levels in juvenile mice developmentally exposed to a human-relevant mixture of polychlorinated biphenyls. *Curr. Res. Toxicol.* 1 85–103. 10.1016/j.crtox.2020.09.001 34296199 PMC8294704

[B48] McCarthy M. M. (2016). Sex differences in the developing brain as a source of inherent risk. *Dialogues Clin. Neurosci.* 18 361–372. 10.31887/dcns.2016.18.4/mmccarthy 28179808 PMC5286722

[B49] Panesar H. K. Kennedy C. L. Keil Stietz K. P. Lein P. J. (2020). Polychlorinated Biphenyls (PCBs): risk factors for autism spectrum disorder? *Toxics* 8:70. 10.3390/toxics8030070 32957475 PMC7560399

[B50] Pessah I. N. Cherednichenko G. Lein P. J. (2010). Minding the calcium store: ryanodine receptor activation as a convergent mechanism of PCB toxicity. *Pharmacol. Ther.* 125 260–285. 10.1016/j.pharmthera.2009.10.009 19931307 PMC2823855

[B51] Pessah I. N. Lein P. J. Seegal R. F. Sagiv S. K. (2019). Neurotoxicity of polychlorinated biphenyls and related organohalogens. *Acta Neuropathol.* 138 363–387. 10.1007/s00401-019-01978-1 30976975 PMC6708608

[B52] Redmond L. Kashani A. H. Ghosh A. (2002). Calcium regulation of dendritic growth *via* CaM kinase IV and CREB-mediated transcription. *Neuron* 34 999–1010. 10.1016/s0896-6273(02)00737-7 12086646

[B53] Robin G. Lopez J. R. Espinal G. M. Hulsizer S. Hagerman P. J. Pessah I. N. (2017). Calcium dysregulation and Cdk5-ATM pathway involved in a mouse model of fragile X-associated tremor/ataxia syndrome. *Hum. Mol. Genet.* 26 2649–2666. 10.1093/hmg/ddx148 28444183 PMC5886271

[B54] Roegge C. S. Morris J. R. Villareal S. Wang V. C. Powers B. E. Klintsova A. Y. (2006). Purkinje cell and cerebellar effects following developmental exposure to PCBs and/or MeHg. *Neurotoxicol. Teratol.* 28 74–85. 10.1016/j.ntt.2005.10.001 16309888

[B55] Rude K. M. Pusceddu M. M. Keogh C. E. Sladek J. A. Rabasa G. Miller E. N. (2019). Developmental exposure to polychlorinated biphenyls (PCBs) in the maternal diet causes host-microbe defects in weanling offspring mice. *Environ. Pollut.* 253 708–721. 10.1016/j.envpol.2019.07.066 31336350 PMC6719698

[B56] Sable H. J. K. Schantz S. L. (2006). “Executive function following developmental exposure to Polychlorinated Biphenyls (PCBs): what animal models have told us,” in *Animal Models of Cognitive Impairment*, eds Levin E. D. Buccafusco J. J. (Boca Raton: CRC Press), 22. 21204372

[B57] Schantz S. L. Widholm J. J. Rice D. C. (2003). Effects of PCB exposure on neuropsychological function in children. *Environ. Health Perspect.* 111 357–576. 10.1289/ehp.5461 12611666 PMC1241394

[B58] Segal I. Korkotian I. Murphy D. D. (2000). Dendritic spine formation and pruning: common cellular mechanisms? *Trends Neurosci.* 23 53–57. 10.1016/s0166-2236(99)01499-x 10652540

[B59] Selby L. Zhang C. Sun Q. Q. (2007). Major defects in neocortical GABAergic inhibitory circuits in mice lacking the fragile X mental retardation protein. *Neurosci. Lett.* 412 227–232. 10.1016/j.neulet.2006.11.062 17197085 PMC1839948

[B60] Sethi S. Keil K. P. Lein P. J. (2017). Species and Sex Differences in the Morphogenic Response of Primary Rodent Neurons to 3,3’-Dichlorobiphenyl (PCB 11). *Toxics* 6:4. 10.3390/toxics6010004 29295518 PMC5874777

[B61] Sethi S. Keil K. P. Lein P. J. (2018). 3,3’-Dichlorobiphenyl (PCB 11) promotes dendritic arborization in primary rat cortical neurons *via* a CREB-dependent mechanism. *Arch. Toxicol.* 92 3337–3345. 10.1007/s00204-018-2307-8 30225637 PMC6196112

[B62] Sethi S. Morgan R. K. Feng W. Lin Y. Li X. Luna C. (2019). Comparative analyses of the 12 most abundant PCB congeners detected in human maternal serum for activity at the thyroid hormone receptor and ryanodine receptor. *Environ. Sci. Technol.* 53 3948–3958. 10.1021/acs.est.9b00535 30821444 PMC6457253

[B64] Simmons S. C. Wheeler K. Mazei-Robison M. S. (2019). Determination of circuit-specific morphological adaptations in ventral tegmental area dopamine neurons by chronic morphine. *Mol. Brain* 12:10. 10.1186/s13041-019-0435-6 30736837 PMC6368752

[B65] Stamou M. Streifel K. M. Goines P. E. Lein P. J. (2013). Neuronal connectivity as a convergent target of gene x environment interactions that confer risk for Autism Spectrum Disorders. *Neurotoxicol. Teratol.* 36 3–16. 10.1016/j.ntt.2012.12.001 23269408 PMC3610799

[B66] Stamou M. Wu X. Kania-Korwel I. Lehmler H. J. Lein P. J. (2014). Cytochrome p450 mRNA expression in the rodent brain: species-, sex-, and region-dependent differences. *Drug Metab. Dispos.* 42 239–244. 10.1124/dmd.113.054239 24255117 PMC3912540

[B67] Ta T. A. Pessah I. N. (2007). Ryanodine receptor type 1 (RyR1) possessing malignant hyperthermia mutation R615C exhibits heightened sensitivity to dysregulation by non-coplanar 2,2’,3,5’,6-pentachlorobiphenyl (PCB 95). *Neurotoxicology* 28 770–779. 10.1016/j.neuro.2006.08.007 17023049 PMC2274001

[B68] Tassone F. Iong K. P. Tong T. H. Lo J. Gane L. W. Berry-Kravis E. (2012). FMR1 CGG allele size and prevalence ascertained through newborn screening in the United States. *Genome Med.* 4:100. 10.1186/gm401 23259642 PMC4064316

[B69] Urbanska M. Gozdz A. Swiech L. J. Jaworski J. (2012). Mammalian target of rapamycin complex 1 (mTORC1) and 2 (mTORC2) control the dendritic arbor morphology of hippocampal neurons. *J. Biol. Chem.* 287 30240–30256. 10.1074/jbc.M112.374405 22810227 PMC3436277

[B70] Wang T. Bray S. M. Warren S. T. (2012). New perspectives on the biology of fragile X syndrome. *Curr. Opin. Genet. Dev.* 22 256–263. 10.1016/j.gde.2012.02.002 22382129 PMC3653273

[B71] Wayman G. A. Bose D. D. Yang D. Lesiak A. Bruun D. Impey S. (2012a). PCB-95 modulates the calcium-dependent signaling pathway responsible for activity-dependent dendritic growth. *Environ. Health Perspect.* 120 1003–1009. 10.1289/ehp.1104833 22534176 PMC3404671

[B72] Wayman G. A. Yang D. Bose D. D. Lesiak A. Ledoux V. Bruun D. (2012b). PCB-95 promotes dendritic growth *via* ryanodine receptor-dependent mechanisms. *Environ. Health Perspect.* 120 997–1002. 10.1289/ehp.1104832 22534141 PMC3404670

[B73] Wayman G. A. Impey S. Marks D. Saneyoshi T. Grant W. F. Derkach V. (2006). Activity-dependent dendritic arborization mediated by CaM-kinase I activation and enhanced CREB-dependent transcription of Wnt-2. *Neuron* 50 897–909. 10.1016/j.neuron.2006.05.008 16772171

[B74] Willemsen R. Hoogeveen-Westerveld M. Reis S. Holstege J. Severijnen L. A. I Nieuwenhuizen M. (2003). The FMR1 CGG repeat mouse displays ubiquitin-positive intranuclear neuronal inclusions; implications for the cerebellar tremor/ataxia syndrome. *Hum. Mol. Genet.* 12 949–959. 10.1093/hmg/ddg114 12700164

[B75] Wilson M. D. Sethi S. Lein P. J. Keil K. P. (2017). Valid statistical approaches for analyzing sholl data: mixed effects versus simple linear models. *J. Neurosci. Methods* 279 33–43. 10.1016/j.jneumeth.2017.01.003 28104486 PMC5346342

[B76] Xi T. Wu J. (2021). A review on the mechanism between different factors and the occurrence of autism and ADHD. *Psychol. Res. Behav. Manag.* 14 393–403. 10.2147/PRBM.S304450 33859505 PMC8044340

[B77] Yang D. Kania-Korwel I. Ghogha A. Chen H. Stamou M. Bose D. D. I (2014). PCB 136 atropselectively alters morphometric and functional parameters of neuronal connectivity in cultured rat hippocampal neurons *via* ryanodine receptor-dependent mechanisms. *Toxicol. Sci.* 138 379–392. 10.1093/toxsci/kft334 24385416 PMC4007107

[B78] Yang D. Kim K. H. Phimister A. Bachstetter A. D. Ward T. R. Stackman R. W. (2009). Developmental exposure to polychlorinated biphenyls interferes with experience-dependent dendritic plasticity and ryanodine receptor expression in weanling rats. *Environ. Health Perspect.* 117 426–435. 10.1289/ehp.11771 19337518 PMC2661913

[B79] Yuen B. Boncompagni S. Feng W. Yang T. Lopez J. R. Matthaei K. I. (2012). Mice expressing T4826I-RYR1 are viable but exhibit sex- and genotype-dependent susceptibility to malignant hyperthermia and muscle damage. *FASEB J.* 26 1311–1322. 10.1096/fj.11-197582 22131268 PMC3289506

