# Supplementary material for: Sex and Genotype Modulate the Dendritic Effects of Developmental Exposure to a Human-Relevant Polychlorinated Biphenyls Mixture in the Juvenile Mouse
Source: Front Neurosci. 2021 Dec 3;15:766802. doi: 10.3389/fnins.2021.766802 (PMC8678536; doi:10.3389/fnins.2021.766802)
Supplement: Supplementary file 1 [file Data_Sheet_1.PDF]

Cortex Sholl Profile Analysis

The Mixed Procedure

Solution for Fixed Effects											
**Effect**	**Sex**	**Genotype**	**Dose**	**Estimate**	**Standard Error**	**DF**	**t Value**	**Pr > |t|**	**Alpha**	**Lower**	**Upper**
**Intercept**				1.7541	0.1086	175	16.16	<.0001	0.05	1.5398	1.9683
**Sex**	F			0.02807	0.05272	42E3	0.53	0.5944	0.05	-0.07526	0.1314
**Sex**	M			0	.	.	.	.	.	.	.
**Genotype**		CG		0.3795	0.1488	42E3	2.55	0.0107	0.05	0.08797	0.6711
**Genotype**		DM		0.06451	0.1488	42E3	0.43	0.6645	0.05	-0.2270	0.3561
**Genotype**		RY		-0.01348	0.1489	42E3	-0.09	0.9279	0.05	-0.3053	0.2784
**Genotype**		WT		0	.	.	.	.	.	.	.
**Dose**			0.1	0.1477	0.1489	42E3	0.99	0.3212	0.05	-0.1441	0.4396
**Dose**			1	0.1035	0.1495	42E3	0.69	0.4885	0.05	-0.1894	0.3965
**Dose**			6	0.2394	0.1491	42E3	1.61	0.1083	0.05	-0.05278	0.5315
**Dose**			0	0	.	.	.	.	.	.	.
**Genotype*Dose**		CG	0.1	-0.3273	0.2104	42E3	-1.56	0.1197	0.05	-0.7396	0.08502
**Genotype*Dose**		CG	1	-0.2455	0.2109	42E3	-1.16	0.2443	0.05	-0.6588	0.1678
**Genotype*Dose**		CG	6	-0.6770	0.2107	42E3	-3.21	0.0013	0.05	-1.0900	-0.2641
**Genotype*Dose**		CG	0	0	.	.	.	.	.	.	.
**Genotype*Dose**		DM	0.1	-0.2038	0.2105	42E3	-0.97	0.3330	0.05	-0.6163	0.2088
**Genotype*Dose**		DM	1	0.2928	0.2109	42E3	1.39	0.1649	0.05	-0.1205	0.7061
**Genotype*Dose**		DM	6	-0.3065	0.2109	42E3	-1.45	0.1462	0.05	-0.7199	0.1069
**Genotype*Dose**		DM	0	0	.	.	.	.	.	.	.
**Genotype*Dose**		RY	0.1	0.1080	0.2107	42E3	0.51	0.6084	0.05	-0.3050	0.5209
**Genotype*Dose**		RY	1	0.2132	0.2109	42E3	1.01	0.3121	0.05	-0.2002	0.6265
**Genotype*Dose**		RY	6	-0.1652	0.2112	42E3	-0.78	0.4341	0.05	-0.5791	0.2487
**Genotype*Dose**		RY	0	0	.	.	.	.	.	.	.
**Genotype*Dose**		WT	0.1	0	.	.	.	.	.	.	.
**Genotype*Dose**		WT	1	0	.	.	.	.	.	.	.
**Genotype*Dose**		WT	6	0	.	.	.	.	.	.	.
**Genotype*Dose**		WT	0	0	.	.	.	.	.	.	.

Cortex Sholl Profile Analysis

The Mixed Procedure

Type 3 Tests of Fixed Effects				
Effect	Num DF	Den DF	F Value	Pr > F
Sex	1	42E3	0.28	0.5944
Genotype	3	42E3	0.31	0.8152
Dose	3	42E3	3.10	0.0257
Genotype*Dose	9	42E3	2.39	0.0106

Least Squares Means					
Effect	Sex	Genotype	Dose	Estimate	Standard Error
Sex	F			1.9305	0.03727
Sex	M			1.9024	0.03728
Genotype		CG		1.9578	0.05262
Genotype		DM		1.9009	0.05270
Genotype		RY		1.9163	0.05278
Genotype		WT		1.8908	0.05277
Dose			0.1	1.9177	0.05265
Dose			1	2.0444	0.05272
Dose			6	1.8279	0.05291
Dose			0	1.8757	0.05259
Genotype*Dose		CG	0.1	1.9680	0.1051
Genotype*Dose		CG	1	2.0057	0.1053
Genotype*Dose		CG	6	1.7100	0.1055
Genotype*Dose		CG	0	2.1476	0.1051
Genotype*Dose		DM	0.1	1.7766	0.1053
Genotype*Dose		DM	1	2.2290	0.1053
Genotype*Dose		DM	6	1.7655	0.1060
Genotype*Dose		DM	0	1.8326	0.1051
Genotype*Dose		RY	0.1	2.0103	0.1055
Genotype*Dose		RY	1	2.0714	0.1051
Genotype*Dose		RY	6	1.8288	0.1063
Genotype*Dose		RY	0	1.7546	0.1053
Genotype*Dose		WT	0.1	1.9158	0.1053
Genotype*Dose		WT	1	1.8716	0.1061

Cortex Sholl Profile Analysis

The Mixed Procedure

Least Squares Means					
Effect	Sex	Genotype	Dose	Estimate	Standard Error
Genotype*Dose		WT	6	2.0075	0.1055
Genotype*Dose		WT	0	1.7681	0.1053

Differences of Least Squares Means											
Effect	Sex	Genotype	Dose	Sex	Genotype	Dose	Estimate	Standard Error	DF	t Value	Pr > |t|
Sex	F			M			0.02807	0.05272	42E3	0.53	0.5944
Genotype		CG			DM		0.05691	0.07447	42E3	0.76	0.4448
Genotype		CG			RY		0.04156	0.07452	42E3	0.56	0.5770
Genotype		CG			WT		0.06707	0.07452	42E3	0.90	0.3681
Genotype		DM			RY		-0.01535	0.07459	42E3	-0.21	0.8370
Genotype		DM			WT		0.01016	0.07458	42E3	0.14	0.8917
Genotype		RY			WT		0.02550	0.07463	42E3	0.34	0.7326
Dose			0.1			1	-0.1267	0.07451	42E3	-1.70	0.0889
Dose			0.1			6	0.08974	0.07464	42E3	1.20	0.2292
Dose			0.1			0	0.04193	0.07441	42E3	0.56	0.5731
Dose			1			6	0.2165	0.07469	42E3	2.90	0.0038
Dose			1			0	0.1687	0.07447	42E3	2.26	0.0235
Dose			6			0	-0.04781	0.07460	42E3	-0.64	0.5216
Genotype*Dose		CG	0.1		CG	1	-0.03762	0.1488	42E3	-0.25	0.8003
Genotype*Dose		CG	0.1		CG	6	0.2580	0.1489	42E3	1.73	0.0831
Genotype*Dose		CG	0.1		CG	0	-0.1796	0.1486	42E3	-1.21	0.2268
Genotype*Dose		CG	0.1		DM	0.1	0.1915	0.1488	42E3	1.29	0.1981
Genotype*Dose		CG	0.1		DM	1	-0.2610	0.1488	42E3	-1.75	0.0794
Genotype*Dose		CG	0.1		DM	6	0.2026	0.1492	42E3	1.36	0.1747
Genotype*Dose		CG	0.1		DM	0	0.1354	0.1486	42E3	0.91	0.3622
Genotype*Dose		CG	0.1		RY	0.1	-0.04225	0.1489	42E3	-0.28	0.7766
Genotype*Dose		CG	0.1		RY	1	-0.1033	0.1486	42E3	-0.70	0.4869
Genotype*Dose		CG	0.1		RY	6	0.1392	0.1495	42E3	0.93	0.3515
Genotype*Dose		CG	0.1		RY	0	0.2134	0.1488	42E3	1.43	0.1514
Genotype*Dose		CG	0.1		WT	0.1	0.05222	0.1488	42E3	0.35	0.7255
Genotype*Dose		CG	0.1		WT	1	0.09639	0.1493	42E3	0.65	0.5186

Cortex Sholl Profile Analysis

The Mixed Procedure

Differences of Least Squares Means											
**Effect**	**Sex**	**Genotype**	**Dose**	**Sex**	**Genotype**	**Dose**	**Estimate**	**Standard Error**	**DF**	**t Value**	**Pr > |t|**
**Genotype*Dose**		CG	0.1		WT	6	-0.03944	0.1489	42E3	-0.26	0.7911
**Genotype*Dose**		CG	0.1		WT	0	0.1999	0.1488	42E3	1.34	0.1789
**Genotype*Dose**		CG	1		CG	6	0.2957	0.1491	42E3	1.98	0.0473
**Genotype*Dose**		CG	1		CG	0	-0.1420	0.1488	42E3	-0.95	0.3399
**Genotype*Dose**		CG	1		DM	0.1	0.2291	0.1489	42E3	1.54	0.1239
**Genotype*Dose**		CG	1		DM	1	-0.2233	0.1489	42E3	-1.50	0.1336
**Genotype*Dose**		CG	1		DM	6	0.2402	0.1494	42E3	1.61	0.1079
**Genotype*Dose**		CG	1		DM	0	0.1730	0.1488	42E3	1.16	0.2447
**Genotype*Dose**		CG	1		RY	0.1	-0.00463	0.1491	42E3	-0.03	0.9752
**Genotype*Dose**		CG	1		RY	1	-0.06569	0.1488	42E3	-0.44	0.6588
**Genotype*Dose**		CG	1		RY	6	0.1769	0.1496	42E3	1.18	0.2372
**Genotype*Dose**		CG	1		RY	0	0.2510	0.1489	42E3	1.69	0.0918
**Genotype*Dose**		CG	1		WT	0.1	0.08984	0.1489	42E3	0.60	0.5463
**Genotype*Dose**		CG	1		WT	1	0.1340	0.1495	42E3	0.90	0.3699
**Genotype*Dose**		CG	1		WT	6	-0.00182	0.1491	42E3	-0.01	0.9903
**Genotype*Dose**		CG	1		WT	0	0.2376	0.1489	42E3	1.60	0.1106
**Genotype*Dose**		CG	6		CG	0	-0.4376	0.1489	42E3	-2.94	0.0033
**Genotype*Dose**		CG	6		DM	0.1	-0.06658	0.1491	42E3	-0.45	0.6551
**Genotype*Dose**		CG	6		DM	1	-0.5190	0.1491	42E3	-3.48	0.0005
**Genotype*Dose**		CG	6		DM	6	-0.05549	0.1495	42E3	-0.37	0.7106
**Genotype*Dose**		CG	6		DM	0	-0.1226	0.1489	42E3	-0.82	0.4102
**Genotype*Dose**		CG	6		RY	0.1	-0.3003	0.1492	42E3	-2.01	0.0442
**Genotype*Dose**		CG	6		RY	1	-0.3614	0.1489	42E3	-2.43	0.0153
**Genotype*Dose**		CG	6		RY	6	-0.1188	0.1498	42E3	-0.79	0.4276
**Genotype*Dose**		CG	6		RY	0	-0.04464	0.1491	42E3	-0.30	0.7646
**Genotype*Dose**		CG	6		WT	0.1	-0.2058	0.1491	42E3	-1.38	0.1673
**Genotype*Dose**		CG	6		WT	1	-0.1617	0.1496	42E3	-1.08	0.2799
**Genotype*Dose**		CG	6		WT	6	-0.2975	0.1492	42E3	-1.99	0.0462
**Genotype*Dose**		CG	6		WT	0	-0.05812	0.1491	42E3	-0.39	0.6966
**Genotype*Dose**		CG	0		DM	0.1	0.3711	0.1488	42E3	2.49	0.0126
**Genotype*Dose**		CG	0		DM	1	-0.08137	0.1488	42E3	-0.55	0.5844
**Genotype*Dose**		CG	0		DM	6	0.3822	0.1492	42E3	2.56	0.0105

Cortex Sholl Profile Analysis

The Mixed Procedure

Differences of Least Squares Means											
**Effect**	**Sex**	**Genotype**	**Dose**	**Sex**	**Genotype**	**Dose**	**Estimate**	**Standard Error**	**DF**	**t Value**	**Pr > |t|**
**Genotype*Dose**		CG	0		DM	0	0.3150	0.1486	42E3	2.12	0.0340
**Genotype*Dose**		CG	0		RY	0.1	0.1373	0.1489	42E3	0.92	0.3563
**Genotype*Dose**		CG	0		RY	1	0.07628	0.1486	42E3	0.51	0.6078
**Genotype*Dose**		CG	0		RY	6	0.3188	0.1495	42E3	2.13	0.0329
**Genotype*Dose**		CG	0		RY	0	0.3930	0.1488	42E3	2.64	0.0082
**Genotype*Dose**		CG	0		WT	0.1	0.2318	0.1488	42E3	1.56	0.1191
**Genotype*Dose**		CG	0		WT	1	0.2760	0.1493	42E3	1.85	0.0646
**Genotype*Dose**		CG	0		WT	6	0.1402	0.1489	42E3	0.94	0.3466
**Genotype*Dose**		CG	0		WT	0	0.3795	0.1488	42E3	2.55	0.0107
**Genotype*Dose**		DM	0.1		DM	1	-0.4524	0.1489	42E3	-3.04	0.0024
**Genotype*Dose**		DM	0.1		DM	6	0.01109	0.1494	42E3	0.07	0.9408
**Genotype*Dose**		DM	0.1		DM	0	-0.05605	0.1488	42E3	-0.38	0.7063
**Genotype*Dose**		DM	0.1		RY	0.1	-0.2337	0.1491	42E3	-1.57	0.1169
**Genotype*Dose**		DM	0.1		RY	1	-0.2948	0.1488	42E3	-1.98	0.0476
**Genotype*Dose**		DM	0.1		RY	6	-0.05222	0.1496	42E3	-0.35	0.7271
**Genotype*Dose**		DM	0.1		RY	0	0.02194	0.1489	42E3	0.15	0.8828
**Genotype*Dose**		DM	0.1		WT	0.1	-0.1392	0.1489	42E3	-0.94	0.3497
**Genotype*Dose**		DM	0.1		WT	1	-0.09508	0.1495	42E3	-0.64	0.5247
**Genotype*Dose**		DM	0.1		WT	6	-0.2309	0.1491	42E3	-1.55	0.1214
**Genotype*Dose**		DM	0.1		WT	0	0.008462	0.1489	42E3	0.06	0.9547
**Genotype*Dose**		DM	1		DM	6	0.4635	0.1494	42E3	3.10	0.0019
**Genotype*Dose**		DM	1		DM	0	0.3964	0.1488	42E3	2.66	0.0077
**Genotype*Dose**		DM	1		RY	0.1	0.2187	0.1491	42E3	1.47	0.1423
**Genotype*Dose**		DM	1		RY	1	0.1576	0.1488	42E3	1.06	0.2893
**Genotype*Dose**		DM	1		RY	6	0.4002	0.1496	42E3	2.67	0.0075
**Genotype*Dose**		DM	1		RY	0	0.4744	0.1489	42E3	3.19	0.0014
**Genotype*Dose**		DM	1		WT	0.1	0.3132	0.1489	42E3	2.10	0.0354
**Genotype*Dose**		DM	1		WT	1	0.3574	0.1495	42E3	2.39	0.0168
**Genotype*Dose**		DM	1		WT	6	0.2215	0.1491	42E3	1.49	0.1372
**Genotype*Dose**		DM	1		WT	0	0.4609	0.1489	42E3	3.10	0.0020
**Genotype*Dose**		DM	6		DM	0	-0.06714	0.1492	42E3	-0.45	0.6528
**Genotype*Dose**		DM	6		RY	0.1	-0.2448	0.1495	42E3	-1.64	0.1016

Cortex Sholl Profile Analysis

The Mixed Procedure

Differences of Least Squares Means											
**Effect**	**Sex**	**Genotype**	**Dose**	**Sex**	**Genotype**	**Dose**	**Estimate**	**Standard Error**	**DF**	**t Value**	**Pr > |t|**
**Genotype*Dose**		DM	6		RY	1	-0.3059	0.1493	42E3	-2.05	0.0404
**Genotype*Dose**		DM	6		RY	6	-0.06331	0.1501	42E3	-0.42	0.6732
**Genotype*Dose**		DM	6		RY	0	0.01085	0.1494	42E3	0.07	0.9421
**Genotype*Dose**		DM	6		WT	0.1	-0.1503	0.1494	42E3	-1.01	0.3143
**Genotype*Dose**		DM	6		WT	1	-0.1062	0.1499	42E3	-0.71	0.4789
**Genotype*Dose**		DM	6		WT	6	-0.2420	0.1495	42E3	-1.62	0.1056
**Genotype*Dose**		DM	6		WT	0	-0.00263	0.1494	42E3	-0.02	0.9860
**Genotype*Dose**		DM	0		RY	0.1	-0.1777	0.1489	42E3	-1.19	0.2328
**Genotype*Dose**		DM	0		RY	1	-0.2387	0.1486	42E3	-1.61	0.1082
**Genotype*Dose**		DM	0		RY	6	0.003827	0.1495	42E3	0.03	0.9796
**Genotype*Dose**		DM	0		RY	0	0.07799	0.1488	42E3	0.52	0.6001
**Genotype*Dose**		DM	0		WT	0.1	-0.08320	0.1488	42E3	-0.56	0.5760
**Genotype*Dose**		DM	0		WT	1	-0.03903	0.1493	42E3	-0.26	0.7938
**Genotype*Dose**		DM	0		WT	6	-0.1749	0.1489	42E3	-1.17	0.2403
**Genotype*Dose**		DM	0		WT	0	0.06451	0.1488	42E3	0.43	0.6645
**Genotype*Dose**		RY	0.1		RY	1	-0.06107	0.1489	42E3	-0.41	0.6818
**Genotype*Dose**		RY	0.1		RY	6	0.1815	0.1498	42E3	1.21	0.2256
**Genotype*Dose**		RY	0.1		RY	0	0.2557	0.1491	42E3	1.72	0.0863
**Genotype*Dose**		RY	0.1		WT	0.1	0.09447	0.1491	42E3	0.63	0.5262
**Genotype*Dose**		RY	0.1		WT	1	0.1386	0.1496	42E3	0.93	0.3541
**Genotype*Dose**		RY	0.1		WT	6	0.002810	0.1492	42E3	0.02	0.9850
**Genotype*Dose**		RY	0.1		WT	0	0.2422	0.1491	42E3	1.62	0.1042
**Genotype*Dose**		RY	1		RY	6	0.2426	0.1495	42E3	1.62	0.1047
**Genotype*Dose**		RY	1		RY	0	0.3167	0.1488	42E3	2.13	0.0333
**Genotype*Dose**		RY	1		WT	0.1	0.1555	0.1488	42E3	1.05	0.2958
**Genotype*Dose**		RY	1		WT	1	0.1997	0.1493	42E3	1.34	0.1811
**Genotype*Dose**		RY	1		WT	6	0.06388	0.1489	42E3	0.43	0.6680
**Genotype*Dose**		RY	1		WT	0	0.3032	0.1488	42E3	2.04	0.0415
**Genotype*Dose**		RY	6		RY	0	0.07416	0.1496	42E3	0.50	0.6201
**Genotype*Dose**		RY	6		WT	0.1	-0.08702	0.1496	42E3	-0.58	0.5608
**Genotype*Dose**		RY	6		WT	1	-0.04286	0.1502	42E3	-0.29	0.7754
**Genotype*Dose**		RY	6		WT	6	-0.1787	0.1498	42E3	-1.19	0.2328

Cortex Sholl Profile Analysis

The Mixed Procedure

Differences of Least Squares Means											
**Effect**	**Sex**	**Genotype**	**Dose**	**Sex**	**Genotype**	**Dose**	**Estimate**	**Standard Error**	**DF**	**t Value**	**Pr > |t|**
**Genotype*Dose**		RY	6		WT	0	0.06068	0.1496	42E3	0.41	0.6850
**Genotype*Dose**		RY	0		WT	0.1	-0.1612	0.1489	42E3	-1.08	0.2790
**Genotype*Dose**		RY	0		WT	1	-0.1170	0.1495	42E3	-0.78	0.4337
**Genotype*Dose**		RY	0		WT	6	-0.2528	0.1491	42E3	-1.70	0.0898
**Genotype*Dose**		RY	0		WT	0	-0.01348	0.1489	42E3	-0.09	0.9279
**Genotype*Dose**		WT	0.1		WT	1	0.04417	0.1495	42E3	0.30	0.7676
**Genotype*Dose**		WT	0.1		WT	6	-0.09166	0.1491	42E3	-0.61	0.5386
**Genotype*Dose**		WT	0.1		WT	0	0.1477	0.1489	42E3	0.99	0.3212
**Genotype*Dose**		WT	1		WT	6	-0.1358	0.1496	42E3	-0.91	0.3640
**Genotype*Dose**		WT	1		WT	0	0.1035	0.1495	42E3	0.69	0.4885
**Genotype*Dose**		WT	6		WT	0	0.2394	0.1491	42E3	1.61	0.1083

Mixed model for Peak X

The Mixed Procedure

Solution for Fixed Effects											
**Effect**	**Sex**	**Genotype**	**Dose**	**Estimate**	**Standard Error**	**DF**	**t Value**	**Pr > |t|**	**Alpha**	**Lower**	**Upper**
**Intercept**				29.5272	0.7502	183	39.36	<.0001	0.05	28.0471	31.0073
**Sex**	F			0.1358	0.5313	184	0.26	0.7985	0.05	-0.9124	1.1841
**Sex**	M			0	.	.	.	.	.	.	.
**Genotype**		CG		-0.08644	0.7508	183	-0.12	0.9085	0.05	-1.5677	1.3948
**Genotype**		DM		-0.9275	0.7520	185	-1.23	0.2190	0.05	-2.4111	0.5562
**Genotype**		RY		-0.8436	0.7524	185	-1.12	0.2637	0.05	-2.3281	0.6408
**Genotype**		WT		0	.	.	.	.	.	.	.
**Dose**			0.1	0.6976	0.7492	182	0.93	0.3530	0.05	-0.7805	2.1758
**Dose**			1	1.0320	0.7499	183	1.38	0.1705	0.05	-0.4476	2.5116
**Dose**			6	0.7874	0.7524	185	1.05	0.2967	0.05	-0.6969	2.2717
**Dose**			0	0	.	.	.	.	.	.	.

Type 3 Tests of Fixed Effects				
**Effect**	**Num DF**	**Den DF**	**F Value**	**Pr > F**
**Sex**	1	184	0.07	0.7985
**Genotype**	3	184	0.85	0.4703
**Dose**	3	184	0.70	0.5548

Least Squares Means					
**Effect**	**Sex**	**Genotype**	**Dose**	**Estimate**	**Standard Error**
**Sex**	F			29.8279	0.3757
**Sex**	M			29.6921	0.3757
**Genotype**		CG		30.1379	0.5297
**Genotype**		DM		29.2969	0.5315
**Genotype**		RY		29.3807	0.5321
**Genotype**		WT		30.2244	0.5320
**Dose**			0.1	29.8284	0.5302
**Dose**			1	30.1627	0.5312

Mixed model for Peak X

The Mixed Procedure

Least Squares Means					
**Effect**	**Sex**	**Genotype**	**Dose**	**Estimate**	**Standard Error**
**Dose**			6	29.9181	0.5347
**Dose**			0	29.1307	0.5293

Differences of Least Squares Means											
**Effect**	**Sex**	**Genotype**	**Dose**	**Sex**	**Genotype**	**Dose**	**Estimate**	**Standard Error**	**DF**	**t Value**	**Pr > |t|**
**Sex**	F			M			0.1358	0.5313	184	0.26	0.7985
**Genotype**		CG			DM		0.8410	0.7504	183	1.12	0.2638
**Genotype**		CG			RY		0.7572	0.7508	183	1.01	0.3145
**Genotype**		CG			WT		-0.08644	0.7508	183	-0.12	0.9085
**Genotype**		DM			RY		-0.08385	0.7520	185	-0.11	0.9113
**Genotype**		DM			WT		-0.9275	0.7520	185	-1.23	0.2190
**Genotype**		RY			WT		-0.8436	0.7524	185	-1.12	0.2637
**Dose**			0.1			1	-0.3343	0.7505	183	-0.45	0.6565
**Dose**			0.1			6	-0.08977	0.7529	185	-0.12	0.9052
**Dose**			0.1			0	0.6976	0.7492	182	0.93	0.3530
**Dose**			1			6	0.2446	0.7537	186	0.32	0.7459
**Dose**			1			0	1.0320	0.7499	183	1.38	0.1705
**Dose**			6			0	0.7874	0.7524	185	1.05	0.2967

Mixed model for Peak Y

The Mixed Procedure

Solution for Fixed Effects											
**Effect**	**Sex**	**Genotype**	**Dose**	**Estimate**	**Standard Error**	**DF**	**t Value**	**Pr > |t|**	**Alpha**	**Lower**	**Upper**
**Intercept**				10.0810	0.3140	179	32.11	<.0001	0.05	9.4614	10.7006
**Sex**	F			0.1612	0.3782	178	0.43	0.6704	0.05	-0.5851	0.9076
**Sex**	M			0	.	.	.	.	.	.	.
**Genotype**		CG		0.2541	0.2679	179	0.95	0.3441	0.05	-0.2745	0.7827
**Genotype**		DM		-0.06333	0.2681	179	-0.24	0.8135	0.05	-0.5924	0.4658
**Genotype**		RY		0.1256	0.2682	180	0.47	0.6401	0.05	-0.4036	0.6549
**Genotype**		WT		0	.	.	.	.	.	.	.
**Dose**			0.1	0.2534	0.3784	178	0.67	0.5040	0.05	-0.4933	1.0000
**Dose**			1	0.4156	0.3784	178	1.10	0.2736	0.05	-0.3311	1.1623
**Dose**			6	-0.1790	0.3795	180	-0.47	0.6378	0.05	-0.9278	0.5699
**Dose**			0	0	.	.	.	.	.	.	.
**Sex*Dose**	F		0.1	-0.3458	0.5351	178	-0.65	0.5190	0.05	-1.4017	0.7102
**Sex*Dose**	F		1	0.3818	0.5354	178	0.71	0.4767	0.05	-0.6748	1.4384
**Sex*Dose**	F		6	-0.1059	0.5364	180	-0.20	0.8437	0.05	-1.1643	0.9525
**Sex*Dose**	F		0	0	.	.	.	.	.	.	.
**Sex*Dose**	M		0.1	0	.	.	.	.	.	.	.
**Sex*Dose**	M		1	0	.	.	.	.	.	.	.
**Sex*Dose**	M		6	0	.	.	.	.	.	.	.
**Sex*Dose**	M		0	0	.	.	.	.	.	.	.

Type 3 Tests of Fixed Effects				
**Effect**	**Num DF**	**Den DF**	**F Value**	**Pr > F**
**Sex**	1	179	0.58	0.4492
**Genotype**	3	179	0.55	0.6478
**Dose**	3	179	3.49	0.0170
**Sex*Dose**	3	179	0.64	0.5906

Mixed model for Peak Y

The Mixed Procedure

Least Squares Means					
**Effect**	**Sex**	**Genotype**	**Dose**	**Estimate**	**Standard Error**
**Sex**	F			10.4263	0.1340
**Sex**	M			10.2826	0.1340
**Genotype**		CG		10.5295	0.1892
**Genotype**		DM		10.2120	0.1895
**Genotype**		RY		10.4010	0.1897
**Genotype**		WT		10.2754	0.1897
**Dose**			0.1	10.3212	0.1893
**Dose**			1	10.8472	0.1895
**Dose**			6	10.0088	0.1902
**Dose**			0	10.2407	0.1891

Differences of Least Squares Means											
**Effect**	**Sex**	**Genotype**	**Dose**	**Sex**	**Genotype**	**Dose**	**Estimate**	**Standard Error**	**DF**	**t Value**	**Pr > |t|**
**Sex**	F			M			0.1437	0.1895	179	0.76	0.4492
**Genotype**		CG			DM		0.3174	0.2678	179	1.19	0.2375
**Genotype**		CG			RY		0.1285	0.2679	179	0.48	0.6322
**Genotype**		CG			WT		0.2541	0.2679	179	0.95	0.3441
**Genotype**		DM			RY		-0.1890	0.2681	179	-0.70	0.4819
**Genotype**		DM			WT		-0.06333	0.2681	179	-0.24	0.8135
**Genotype**		RY			WT		0.1256	0.2682	180	0.47	0.6401
**Dose**			0.1			1	-0.5260	0.2678	179	-1.96	0.0511
**Dose**			0.1			6	0.3124	0.2683	180	1.16	0.2458
**Dose**			0.1			0	0.08047	0.2675	178	0.30	0.7639
**Dose**			1			6	0.8384	0.2685	180	3.12	0.0021
**Dose**			1			0	0.6065	0.2677	178	2.27	0.0247
**Dose**			6			0	-0.2319	0.2682	180	-0.86	0.3883

Mixed model for AUC total

The Mixed Procedure

Solution for Fixed Effects											
**Effect**	**Sex**	**Genotype**	**Dose**	**Estimate**	**Standard Error**	**DF**	**t Value**	**Pr > |t|**	**Alpha**	**Lower**	**Upper**
**Intercept**				509.32	31.6586	173	16.09	<.0001	0.05	446.84	571.81
**Sex**	F			6.7592	15.3727	173	0.44	0.6607	0.05	-23.5827	37.1010
**Sex**	M			0	.	.	.	.	.	.	.
**Genotype**		CG		110.11	43.3836	172	2.54	0.0120	0.05	24.4756	195.74
**Genotype**		DM		15.7337	43.3836	172	0.36	0.7173	0.05	-69.8994	101.37
**Genotype**		RY		-3.9220	43.4226	173	-0.09	0.9281	0.05	-89.6300	81.7861
**Genotype**		WT		0	.	.	.	.	.	.	.
**Dose**			0.1	42.8305	43.4226	173	0.99	0.3253	0.05	-42.8775	128.54
**Dose**			1	29.6071	43.5688	175	0.68	0.4977	0.05	-56.3826	115.60
**Dose**			6	69.5275	43.4619	173	1.60	0.1115	0.05	-16.2559	155.31
**Dose**			0	0	.	.	.	.	.	.	.
**Genotype*Dose**		CG	0.1	-94.9139	61.3537	172	-1.55	0.1237	0.05	-216.02	26.1897
**Genotype*Dose**		CG	1	-70.7806	61.4848	173	-1.15	0.2512	0.05	-192.14	50.5752
**Genotype*Dose**		CG	6	-196.43	61.4368	173	-3.20	0.0017	0.05	-317.69	-75.1626
**Genotype*Dose**		CG	0	0	.	.	.	.	.	.	.
**Genotype*Dose**		DM	0.1	-56.1459	61.3813	172	-0.91	0.3616	0.05	-177.30	65.0107
**Genotype*Dose**		DM	1	88.2543	61.4848	173	1.44	0.1530	0.05	-33.1017	209.61
**Genotype*Dose**		DM	6	-94.2500	61.5271	174	-1.53	0.1274	0.05	-215.69	27.1865
**Genotype*Dose**		DM	0	0	.	.	.	.	.	.	.
**Genotype*Dose**		RY	0.1	31.3776	61.4366	173	0.51	0.6102	0.05	-89.8852	152.64
**Genotype*Dose**		RY	1	62.2473	61.4892	173	1.01	0.3128	0.05	-59.1171	183.61
**Genotype*Dose**		RY	6	-48.0926	61.5686	174	-0.78	0.4358	0.05	-169.61	73.4242
**Genotype*Dose**		RY	0	0	.	.	.	.	.	.	.
**Genotype*Dose**		WT	0.1	0	.	.	.	.	.	.	.
**Genotype*Dose**		WT	1	0	.	.	.	.	.	.	.
**Genotype*Dose**		WT	6	0	.	.	.	.	.	.	.
**Genotype*Dose**		WT	0	0	.	.	.	.	.	.	.

Mixed model for AUC total

The Mixed Procedure

Type 3 Tests of Fixed Effects				
**Effect**	**Num DF**	**Den DF**	**F Value**	**Pr > F**
**Sex**	1	173	0.19	0.6607
**Genotype**	3	173	0.36	0.7832
**Dose**	3	173	3.23	0.0239
**Genotype*Dose**	9	173	2.40	0.0136

Least Squares Means					
**Effect**	**Sex**	**Genotype**	**Dose**	**Estimate**	**Standard Error**
**Sex**	F			558.38	10.8703
**Sex**	M			551.63	10.8702
**Genotype**		CG		567.77	15.3454
**Genotype**		DM		548.39	15.3748
**Genotype**		RY		555.66	15.3863
**Genotype**		WT		548.20	15.3850
**Dose**			0.1	556.09	15.3523
**Dose**			1	592.72	15.3723
**Dose**			6	528.02	15.4285
**Dose**			0	543.18	15.3384
**Genotype*Dose**		CG	0.1	570.73	30.6492
**Genotype*Dose**		CG	1	581.64	30.7044
**Genotype*Dose**		CG	6	495.91	30.7599
**Genotype*Dose**		CG	0	622.81	30.6492
**Genotype*Dose**		DM	0.1	515.12	30.7044
**Genotype*Dose**		DM	1	646.30	30.7044
**Genotype*Dose**		DM	6	503.71	30.9399
**Genotype*Dose**		DM	0	528.44	30.6492
**Genotype*Dose**		RY	0.1	582.99	30.7599
**Genotype*Dose**		RY	1	600.64	30.6579
**Genotype*Dose**		RY	6	530.22	30.9675
**Genotype*Dose**		RY	0	508.78	30.7044
**Genotype*Dose**		WT	0.1	555.53	30.7044
**Genotype*Dose**		WT	1	542.31	30.9108

Mixed model for AUC total

The Mixed Procedure

Least Squares Means					
**Effect**	**Sex**	**Genotype**	**Dose**	**Estimate**	**Standard Error**
**Genotype*Dose**		WT	6	582.23	30.7599
**Genotype*Dose**		WT	0	512.70	30.7044

Differences of Least Squares Means											
**Effect**	**Sex**	**Genotype**	**Dose**	**Sex**	**Genotype**	**Dose**	**Estimate**	**Standard Error**	**DF**	**t Value**	**Pr > |t|**
**Sex**	F			M			6.7592	15.3727	173	0.44	0.6607
**Genotype**		CG			DM		19.3804	21.7225	173	0.89	0.3735
**Genotype**		CG			RY		12.1176	21.7306	173	0.56	0.5778
**Genotype**		CG			WT		19.5787	21.7297	173	0.90	0.3688
**Genotype**		DM			RY		-7.2628	21.7514	174	-0.33	0.7389
**Genotype**		DM			WT		0.1983	21.7505	174	0.01	0.9927
**Genotype**		RY			WT		7.4611	21.7586	174	0.34	0.7321
**Dose**			0.1			1	-36.6274	21.7255	173	-1.69	0.0936
**Dose**			0.1			6	28.0746	21.7653	174	1.29	0.1988
**Dose**			0.1			0	12.9100	21.7016	172	0.59	0.5527
**Dose**			1			6	64.7019	21.7795	174	2.97	0.0034
**Dose**			1			0	49.5374	21.7157	173	2.28	0.0238
**Dose**			6			0	-15.1646	21.7556	174	-0.70	0.4867
**Genotype*Dose**		CG	0.1		CG	1	-10.9099	43.3836	172	-0.25	0.8017
**Genotype*Dose**		CG	0.1		CG	6	74.8148	43.4229	173	1.72	0.0867
**Genotype*Dose**		CG	0.1		CG	0	-52.0833	43.3445	171	-1.20	0.2312
**Genotype*Dose**		CG	0.1		DM	0.1	55.6070	43.3836	172	1.28	0.2017
**Genotype*Dose**		CG	0.1		DM	1	-75.5697	43.3836	172	-1.74	0.0833
**Genotype*Dose**		CG	0.1		DM	6	67.0142	43.5506	174	1.54	0.1257
**Genotype*Dose**		CG	0.1		DM	0	42.2917	43.3445	171	0.98	0.3306
**Genotype*Dose**		CG	0.1		RY	0.1	-12.2608	43.4229	173	-0.28	0.7780
**Genotype*Dose**		CG	0.1		RY	1	-29.9071	43.3507	171	-0.69	0.4912
**Genotype*Dose**		CG	0.1		RY	6	40.5124	43.5702	175	0.93	0.3537
**Genotype*Dose**		CG	0.1		RY	0	61.9473	43.3836	172	1.43	0.1551
**Genotype*Dose**		CG	0.1		WT	0.1	15.1948	43.3836	172	0.35	0.7266
**Genotype*Dose**		CG	0.1		WT	1	28.4183	43.5299	174	0.65	0.5147

Mixed model for AUC total

The Mixed Procedure

Differences of Least Squares Means											
**Effect**	**Sex**	**Genotype**	**Dose**	**Sex**	**Genotype**	**Dose**	**Estimate**	**Standard Error**	**DF**	**t Value**	**Pr > |t|**
**Genotype*Dose**		CG	0.1		WT	6	-11.5021	43.4229	173	-0.26	0.7914
**Genotype*Dose**		CG	0.1		WT	0	58.0253	43.3836	172	1.34	0.1828
**Genotype*Dose**		CG	1		CG	6	85.7247	43.4619	173	1.97	0.0502
**Genotype*Dose**		CG	1		CG	0	-41.1735	43.3836	172	-0.95	0.3439
**Genotype*Dose**		CG	1		DM	0.1	66.5169	43.4226	173	1.53	0.1274
**Genotype*Dose**		CG	1		DM	1	-64.6599	43.4226	173	-1.49	0.1383
**Genotype*Dose**		CG	1		DM	6	77.9241	43.5894	175	1.79	0.0756
**Genotype*Dose**		CG	1		DM	0	53.2015	43.3836	172	1.23	0.2218
**Genotype*Dose**		CG	1		RY	0.1	-1.3510	43.4619	173	-0.03	0.9752
**Genotype*Dose**		CG	1		RY	1	-18.9972	43.3897	172	-0.44	0.6621
**Genotype*Dose**		CG	1		RY	6	51.4223	43.6091	175	1.18	0.2399
**Genotype*Dose**		CG	1		RY	0	72.8572	43.4226	173	1.68	0.0952
**Genotype*Dose**		CG	1		WT	0.1	26.1047	43.4226	173	0.60	0.5485
**Genotype*Dose**		CG	1		WT	1	39.3281	43.5687	175	0.90	0.3679
**Genotype*Dose**		CG	1		WT	6	-0.5923	43.4619	173	-0.01	0.9891
**Genotype*Dose**		CG	1		WT	0	68.9352	43.4226	173	1.59	0.1142
**Genotype*Dose**		CG	6		CG	0	-126.90	43.4229	173	-2.92	0.0039
**Genotype*Dose**		CG	6		DM	0.1	-19.2078	43.4619	173	-0.44	0.6591
**Genotype*Dose**		CG	6		DM	1	-150.38	43.4619	173	-3.46	0.0007
**Genotype*Dose**		CG	6		DM	6	-7.8006	43.6285	176	-0.18	0.8583
**Genotype*Dose**		CG	6		DM	0	-32.5232	43.4229	173	-0.75	0.4549
**Genotype*Dose**		CG	6		RY	0.1	-87.0757	43.5011	174	-2.00	0.0469
**Genotype*Dose**		CG	6		RY	1	-104.72	43.4290	173	-2.41	0.0169
**Genotype*Dose**		CG	6		RY	6	-34.3024	43.6481	176	-0.79	0.4330
**Genotype*Dose**		CG	6		RY	0	-12.8675	43.4619	173	-0.30	0.7675
**Genotype*Dose**		CG	6		WT	0.1	-59.6200	43.4619	173	-1.37	0.1719
**Genotype*Dose**		CG	6		WT	1	-46.3966	43.6079	175	-1.06	0.2888
**Genotype*Dose**		CG	6		WT	6	-86.3170	43.5011	174	-1.98	0.0488
**Genotype*Dose**		CG	6		WT	0	-16.7895	43.4619	173	-0.39	0.6997
**Genotype*Dose**		CG	0		DM	0.1	107.69	43.3836	172	2.48	0.0140
**Genotype*Dose**		CG	0		DM	1	-23.4864	43.3836	172	-0.54	0.5890
**Genotype*Dose**		CG	0		DM	6	119.10	43.5506	174	2.73	0.0069

Mixed model for AUC total

The Mixed Procedure

Differences of Least Squares Means											
**Effect**	**Sex**	**Genotype**	**Dose**	**Sex**	**Genotype**	**Dose**	**Estimate**	**Standard Error**	**DF**	**t Value**	**Pr > |t|**
**Genotype*Dose**		CG	0		DM	0	94.3750	43.3445	171	2.18	0.0308
**Genotype*Dose**		CG	0		RY	0.1	39.8225	43.4229	173	0.92	0.3604
**Genotype*Dose**		CG	0		RY	1	22.1763	43.3507	171	0.51	0.6096
**Genotype*Dose**		CG	0		RY	6	92.5957	43.5702	175	2.13	0.0350
**Genotype*Dose**		CG	0		RY	0	114.03	43.3836	172	2.63	0.0094
**Genotype*Dose**		CG	0		WT	0.1	67.2782	43.3836	172	1.55	0.1228
**Genotype*Dose**		CG	0		WT	1	80.5016	43.5299	174	1.85	0.0661
**Genotype*Dose**		CG	0		WT	6	40.5812	43.4229	173	0.93	0.3513
**Genotype*Dose**		CG	0		WT	0	110.11	43.3836	172	2.54	0.0120
**Genotype*Dose**		DM	0.1		DM	1	-131.18	43.4226	173	-3.02	0.0029
**Genotype*Dose**		DM	0.1		DM	6	11.4072	43.5895	175	0.26	0.7939
**Genotype*Dose**		DM	0.1		DM	0	-13.3153	43.3836	172	-0.31	0.7593
**Genotype*Dose**		DM	0.1		RY	0.1	-67.8678	43.4619	173	-1.56	0.1202
**Genotype*Dose**		DM	0.1		RY	1	-85.5141	43.3897	172	-1.97	0.0503
**Genotype*Dose**		DM	0.1		RY	6	-15.0946	43.6090	175	-0.35	0.7297
**Genotype*Dose**		DM	0.1		RY	0	6.3403	43.4226	173	0.15	0.8841
**Genotype*Dose**		DM	0.1		WT	0.1	-40.4122	43.4226	173	-0.93	0.3533
**Genotype*Dose**		DM	0.1		WT	1	-27.1887	43.5688	175	-0.62	0.5334
**Genotype*Dose**		DM	0.1		WT	6	-67.1091	43.4619	173	-1.54	0.1244
**Genotype*Dose**		DM	0.1		WT	0	2.4183	43.4226	173	0.06	0.9557
**Genotype*Dose**		DM	1		DM	6	142.58	43.5895	175	3.27	0.0013
**Genotype*Dose**		DM	1		DM	0	117.86	43.3836	172	2.72	0.0073
**Genotype*Dose**		DM	1		RY	0.1	63.3089	43.4619	173	1.46	0.1470
**Genotype*Dose**		DM	1		RY	1	45.6627	43.3897	172	1.05	0.2941
**Genotype*Dose**		DM	1		RY	6	116.08	43.6090	175	2.66	0.0085
**Genotype*Dose**		DM	1		RY	0	137.52	43.4226	173	3.17	0.0018
**Genotype*Dose**		DM	1		WT	0.1	90.7645	43.4226	173	2.09	0.0381
**Genotype*Dose**		DM	1		WT	1	103.99	43.5688	175	2.39	0.0181
**Genotype*Dose**		DM	1		WT	6	64.0676	43.4619	173	1.47	0.1423
**Genotype*Dose**		DM	1		WT	0	133.60	43.4226	173	3.08	0.0024
**Genotype*Dose**		DM	6		DM	0	-24.7226	43.5506	174	-0.57	0.5710
**Genotype*Dose**		DM	6		RY	0.1	-79.2751	43.6285	176	-1.82	0.0709

Mixed model for AUC total

The Mixed Procedure

Differences of Least Squares Means											
**Effect**	**Sex**	**Genotype**	**Dose**	**Sex**	**Genotype**	**Dose**	**Estimate**	**Standard Error**	**DF**	**t Value**	**Pr > |t|**
**Genotype*Dose**		DM	6		RY	1	-96.9213	43.5567	174	-2.23	0.0274
**Genotype*Dose**		DM	6		RY	6	-26.5018	43.7752	178	-0.61	0.5457
**Genotype*Dose**		DM	6		RY	0	-5.0669	43.5894	175	-0.12	0.9076
**Genotype*Dose**		DM	6		WT	0.1	-51.8194	43.5894	175	-1.19	0.2361
**Genotype*Dose**		DM	6		WT	1	-38.5960	43.7350	177	-0.88	0.3787
**Genotype*Dose**		DM	6		WT	6	-78.5164	43.6285	176	-1.80	0.0736
**Genotype*Dose**		DM	6		WT	0	-8.9889	43.5895	175	-0.21	0.8369
**Genotype*Dose**		DM	0		RY	0.1	-54.5525	43.4229	173	-1.26	0.2107
**Genotype*Dose**		DM	0		RY	1	-72.1987	43.3507	171	-1.67	0.0976
**Genotype*Dose**		DM	0		RY	6	-1.7793	43.5702	175	-0.04	0.9675
**Genotype*Dose**		DM	0		RY	0	19.6557	43.3836	172	0.45	0.6511
**Genotype*Dose**		DM	0		WT	0.1	-27.0968	43.3836	172	-0.62	0.5331
**Genotype*Dose**		DM	0		WT	1	-13.8734	43.5299	174	-0.32	0.7503
**Genotype*Dose**		DM	0		WT	6	-53.7938	43.4229	173	-1.24	0.2171
**Genotype*Dose**		DM	0		WT	0	15.7337	43.3836	172	0.36	0.7173
**Genotype*Dose**		RY	0.1		RY	1	-17.6463	43.4290	173	-0.41	0.6850
**Genotype*Dose**		RY	0.1		RY	6	52.7732	43.6481	176	1.21	0.2283
**Genotype*Dose**		RY	0.1		RY	0	74.2081	43.4619	173	1.71	0.0895
**Genotype*Dose**		RY	0.1		WT	0.1	27.4556	43.4619	173	0.63	0.5284
**Genotype*Dose**		RY	0.1		WT	1	40.6791	43.6079	175	0.93	0.3522
**Genotype*Dose**		RY	0.1		WT	6	0.7587	43.5011	174	0.02	0.9861
**Genotype*Dose**		RY	0.1		WT	0	70.2862	43.4619	173	1.62	0.1077
**Genotype*Dose**		RY	1		RY	6	70.4195	43.5763	175	1.62	0.1079
**Genotype*Dose**		RY	1		RY	0	91.8544	43.3897	172	2.12	0.0357
**Genotype*Dose**		RY	1		WT	0.1	45.1019	43.3897	172	1.04	0.3001
**Genotype*Dose**		RY	1		WT	1	58.3253	43.5360	174	1.34	0.1821
**Genotype*Dose**		RY	1		WT	6	18.4050	43.4290	173	0.42	0.6722
**Genotype*Dose**		RY	1		WT	0	87.9324	43.3897	172	2.03	0.0443
**Genotype*Dose**		RY	6		RY	0	21.4349	43.6091	175	0.49	0.6237
**Genotype*Dose**		RY	6		WT	0.1	-25.3176	43.6091	175	-0.58	0.5623
**Genotype*Dose**		RY	6		WT	1	-12.0942	43.7548	177	-0.28	0.7826
**Genotype*Dose**		RY	6		WT	6	-52.0145	43.6481	176	-1.19	0.2350

Mixed model for AUC total

The Mixed Procedure

Differences of Least Squares Means											
**Effect**	**Sex**	**Genotype**	**Dose**	**Sex**	**Genotype**	**Dose**	**Estimate**	**Standard Error**	**DF**	**t Value**	**Pr > |t|**
**Genotype*Dose**		RY	6		WT	0	17.5129	43.6090	175	0.40	0.6885
**Genotype*Dose**		RY	0		WT	0.1	-46.7525	43.4226	173	-1.08	0.2831
**Genotype*Dose**		RY	0		WT	1	-33.5291	43.5687	175	-0.77	0.4426
**Genotype*Dose**		RY	0		WT	6	-73.4494	43.4619	173	-1.69	0.0928
**Genotype*Dose**		RY	0		WT	0	-3.9220	43.4226	173	-0.09	0.9281
**Genotype*Dose**		WT	0.1		WT	1	13.2234	43.5687	175	0.30	0.7619
**Genotype*Dose**		WT	0.1		WT	6	-26.6969	43.4619	173	-0.61	0.5398
**Genotype*Dose**		WT	0.1		WT	0	42.8305	43.4226	173	0.99	0.3253
**Genotype*Dose**		WT	1		WT	6	-39.9204	43.6079	175	-0.92	0.3612
**Genotype*Dose**		WT	1		WT	0	29.6071	43.5688	175	0.68	0.4977
**Genotype*Dose**		WT	6		WT	0	69.5275	43.4619	173	1.60	0.1115

Mixed model for AUC proximal

The Mixed Procedure

Solution for Fixed Effects										
Effect	Sex	Genotype	Dose	Estimate	Standard Error	DF	t Value	Pr > |t|	Alpha	Lower	Upper
**Intercept**				401.55	19.6077	173	20.48	<.0001	0.05	362.84	440.25
**Sex**	F			7.7445	9.5202	173	0.81	0.4171	0.05	-11.0458	26.5348
**Sex**	M			0	.	.	.	.	.	.	.
**Genotype**		CG		66.6649	26.8726	172	2.48	0.0141	0.05	13.6229	119.71
**Genotype**		DM		11.7691	26.8726	172	0.44	0.6620	0.05	-41.2730	64.8112
**Genotype**		RY		1.3770	26.8944	173	0.05	0.9592	0.05	-51.7070	54.4610
**Genotype**		WT		0	.	.	.	.	.	.	.
**Dose**			0.1	19.8136	26.8944	173	0.74	0.4623	0.05	-33.2704	72.8976
**Dose**			1	20.8590	26.9771	175	0.77	0.4404	0.05	-32.3843	74.1023
**Dose**			6	47.1932	26.9164	173	1.75	0.0813	0.05	-5.9330	100.32
**Dose**			0	0	.	.	.	.	.	.	.
**Genotype*Dose**		CG	0.1	-53.7719	38.0036	172	-1.41	0.1589	0.05	-128.78	21.2409
**Genotype*Dose**		CG	1	-40.7720	38.0775	173	-1.07	0.2858	0.05	-115.93	34.3832
**Genotype*Dose**		CG	6	-126.04	38.0501	173	-3.31	0.0011	0.05	-201.14	-50.9404
**Genotype*Dose**		CG	0	0	.	.	.	.	.	.	.
**Genotype*Dose**		DM	0.1	-28.8586	38.0190	172	-0.76	0.4489	0.05	-103.90	46.1840
**Genotype*Dose**		DM	1	41.2080	38.0776	173	1.08	0.2807	0.05	-33.9473	116.36
**Genotype*Dose**		DM	6	-72.2678	38.1007	174	-1.90	0.0595	0.05	-147.47	2.9316
**Genotype*Dose**		DM	0	0	.	.	.	.	.	.	.
**Genotype*Dose**		RY	0.1	18.8473	38.0500	173	0.50	0.6210	0.05	-56.2546	93.9493
**Genotype*Dose**		RY	1	37.1755	38.0801	173	0.98	0.3303	0.05	-37.9847	112.34
**Genotype*Dose**		RY	6	-35.1399	38.1244	174	-0.92	0.3580	0.05	-110.39	40.1053
**Genotype*Dose**		RY	0	0	.	.	.	.	.	.	.
**Genotype*Dose**		WT	0.1	0	.	.	.	.	.	.	.
**Genotype*Dose**		WT	1	0	.	.	.	.	.	.	.
**Genotype*Dose**		WT	6	0	.	.	.	.	.	.	.
**Genotype*Dose**		WT	0	0	.	.	.	.	.	.	.

Mixed model for AUC proximal

The Mixed Procedure

Type 3 Tests of Fixed Effects				
Effect	Num DF	Den DF	F Value	Pr > F
Sex	1	173	0.66	0.4171
Genotype	3	173	0.48	0.6951
Dose	3	173	3.39	0.0193
Genotype*Dose	9	173	2.21	0.0234

Least Squares Means					
Effect	Sex	Genotype	Dose	Estimate	Standard Error
Sex	F			434.98	6.7318
Sex	M			427.24	6.7318
Genotype		CG		438.90	9.5048
Genotype		DM		424.17	9.5213
Genotype		RY		433.98	9.5279
Genotype		WT		427.38	9.5271
Dose			0.1	429.24	9.5086
Dose			1	455.63	9.5200
Dose			6	414.20	9.5515
Dose			0	425.37	9.5009
Genotype*Dose		CG	0.1	438.13	18.9863
Genotype*Dose		CG	1	452.17	19.0172
Genotype*Dose		CG	6	393.23	19.0483
Genotype*Dose		CG	0	472.08	18.9863
Genotype*Dose		DM	0.1	408.14	19.0172
Genotype*Dose		DM	1	479.25	19.0172
Genotype*Dose		DM	6	392.11	19.1492
Genotype*Dose		DM	0	417.19	18.9863
Genotype*Dose		RY	0.1	445.46	19.0483
Genotype*Dose		RY	1	464.83	18.9914
Genotype*Dose		RY	6	418.85	19.1657
Genotype*Dose		RY	0	406.80	19.0172
Genotype*Dose		WT	0.1	425.23	19.0172
Genotype*Dose		WT	1	426.28	19.1340

Mixed model for AUC proximal

The Mixed Procedure

Least Squares Means					
Effect	Sex	Genotype	Dose	Estimate	Standard Error
Genotype*Dose		WT	6	452.61	19.0483
Genotype*Dose		WT	0	405.42	19.0172

Differences of Least Squares Means											
Effect	Sex	Genotype	Dose	Sex	Genotype	Dose	Estimate	Standard Error	DF	t Value	Pr > |t|
Sex	F			M			7.7445	9.5202	173	0.81	0.4171
Genotype		CG			DM		14.7288	13.4535	173	1.09	0.2751
Genotype		CG			RY		4.9206	13.4581	173	0.37	0.7151
Genotype		CG			WT		11.5183	13.4576	173	0.86	0.3932
Genotype		DM			RY		-9.8082	13.4698	174	-0.73	0.4675
Genotype		DM			WT		-3.2105	13.4693	174	-0.24	0.8119
Genotype		RY			WT		6.5978	13.4739	174	0.49	0.6250
Dose			0.1			1	-26.3940	13.4553	173	-1.96	0.0514
Dose			0.1			6	15.0372	13.4776	174	1.12	0.2661
Dose			0.1			0	3.8678	13.4418	172	0.29	0.7739
Dose			1			6	41.4312	13.4856	175	3.07	0.0025
Dose			1			0	30.2619	13.4498	173	2.25	0.0257
Dose			6			0	-11.1694	13.4721	174	-0.83	0.4082
Genotype*Dose		CG	0.1		CG	1	-14.0453	26.8726	172	-0.52	0.6019
Genotype*Dose		CG	0.1		CG	6	44.8911	26.8945	173	1.67	0.0969
Genotype*Dose		CG	0.1		CG	0	-33.9583	26.8507	172	-1.26	0.2077
Genotype*Dose		CG	0.1		DM	0.1	29.9825	26.8726	172	1.12	0.2661
Genotype*Dose		CG	0.1		DM	1	-41.1295	26.8726	172	-1.53	0.1277
Genotype*Dose		CG	0.1		DM	6	46.0121	26.9661	174	1.71	0.0897
Genotype*Dose		CG	0.1		DM	0	20.9375	26.8507	172	0.78	0.4366
Genotype*Dose		CG	0.1		RY	0.1	-7.3313	26.8945	173	-0.27	0.7855
Genotype*Dose		CG	0.1		RY	1	-26.7049	26.8543	172	-0.99	0.3214
Genotype*Dose		CG	0.1		RY	6	19.2763	26.9778	175	0.71	0.4759
Genotype*Dose		CG	0.1		RY	0	31.3296	26.8726	172	1.17	0.2453
Genotype*Dose		CG	0.1		WT	0.1	12.8930	26.8726	172	0.48	0.6320
Genotype*Dose		CG	0.1		WT	1	11.8476	26.9553	174	0.44	0.6608

Mixed model for AUC proximal

The Mixed Procedure

Differences of Least Squares Means											
**Effect**	**Sex**	**Genotype**	**Dose**	**Sex**	**Genotype**	**Dose**	**Estimate**	**Standard Error**	**DF**	**t Value**	**Pr > |t|**
**Genotype*Dose**		CG	0.1		WT	6	-14.4866	26.8945	173	-0.54	0.5908
**Genotype*Dose**		CG	0.1		WT	0	32.7066	26.8726	172	1.22	0.2252
**Genotype*Dose**		CG	1		CG	6	58.9364	26.9164	173	2.19	0.0299
**Genotype*Dose**		CG	1		CG	0	-19.9130	26.8726	172	-0.74	0.4597
**Genotype*Dose**		CG	1		DM	0.1	44.0278	26.8944	173	1.64	0.1034
**Genotype*Dose**		CG	1		DM	1	-27.0842	26.8944	173	-1.01	0.3153
**Genotype*Dose**		CG	1		DM	6	60.0574	26.9879	175	2.23	0.0273
**Genotype*Dose**		CG	1		DM	0	34.9828	26.8726	172	1.30	0.1947
**Genotype*Dose**		CG	1		RY	0.1	6.7140	26.9164	173	0.25	0.8033
**Genotype*Dose**		CG	1		RY	1	-12.6596	26.8761	172	-0.47	0.6382
**Genotype*Dose**		CG	1		RY	6	33.3216	26.9996	175	1.23	0.2188
**Genotype*Dose**		CG	1		RY	0	45.3749	26.8944	173	1.69	0.0934
**Genotype*Dose**		CG	1		WT	0.1	26.9383	26.8944	173	1.00	0.3179
**Genotype*Dose**		CG	1		WT	1	25.8929	26.9771	175	0.96	0.3385
**Genotype*Dose**		CG	1		WT	6	-0.4413	26.9164	173	-0.02	0.9869
**Genotype*Dose**		CG	1		WT	0	46.7519	26.8944	173	1.74	0.0839
**Genotype*Dose**		CG	6		CG	0	-78.8494	26.8945	173	-2.93	0.0038
**Genotype*Dose**		CG	6		DM	0.1	-14.9086	26.9164	173	-0.55	0.5804
**Genotype*Dose**		CG	6		DM	1	-86.0206	26.9164	173	-3.20	0.0017
**Genotype*Dose**		CG	6		DM	6	1.1210	27.0098	176	0.04	0.9669
**Genotype*Dose**		CG	6		DM	0	-23.9536	26.8945	173	-0.89	0.3744
**Genotype*Dose**		CG	6		RY	0.1	-52.2224	26.9383	174	-1.94	0.0542
**Genotype*Dose**		CG	6		RY	1	-71.5959	26.8981	173	-2.66	0.0085
**Genotype*Dose**		CG	6		RY	6	-25.6148	27.0215	176	-0.95	0.3445
**Genotype*Dose**		CG	6		RY	0	-13.5615	26.9164	173	-0.50	0.6150
**Genotype*Dose**		CG	6		WT	0.1	-31.9981	26.9164	173	-1.19	0.2361
**Genotype*Dose**		CG	6		WT	1	-33.0435	26.9990	175	-1.22	0.2226
**Genotype*Dose**		CG	6		WT	6	-59.3776	26.9383	174	-2.20	0.0288
**Genotype*Dose**		CG	6		WT	0	-12.1845	26.9164	173	-0.45	0.6513
**Genotype*Dose**		CG	0		DM	0.1	63.9408	26.8726	172	2.38	0.0184
**Genotype*Dose**		CG	0		DM	1	-7.1712	26.8726	172	-0.27	0.7899
**Genotype*Dose**		CG	0		DM	6	79.9704	26.9661	174	2.97	0.0034

Mixed model for AUC proximal

The Mixed Procedure

Differences of Least Squares Means											
**Effect**	**Sex**	**Genotype**	**Dose**	**Sex**	**Genotype**	**Dose**	**Estimate**	**Standard Error**	**DF**	**t Value**	**Pr > |t|**
**Genotype*Dose**		CG	0		DM	0	54.8958	26.8507	172	2.04	0.0424
**Genotype*Dose**		CG	0		RY	0.1	26.6270	26.8945	173	0.99	0.3235
**Genotype*Dose**		CG	0		RY	1	7.2535	26.8543	172	0.27	0.7874
**Genotype*Dose**		CG	0		RY	6	53.2346	26.9778	175	1.97	0.0500
**Genotype*Dose**		CG	0		RY	0	65.2879	26.8726	172	2.43	0.0161
**Genotype*Dose**		CG	0		WT	0.1	46.8513	26.8726	172	1.74	0.0830
**Genotype*Dose**		CG	0		WT	1	45.8059	26.9553	174	1.70	0.0910
**Genotype*Dose**		CG	0		WT	6	19.4718	26.8945	173	0.72	0.4700
**Genotype*Dose**		CG	0		WT	0	66.6649	26.8726	172	2.48	0.0141
**Genotype*Dose**		DM	0.1		DM	1	-71.1120	26.8944	173	-2.64	0.0089
**Genotype*Dose**		DM	0.1		DM	6	16.0296	26.9879	175	0.59	0.5533
**Genotype*Dose**		DM	0.1		DM	0	-9.0450	26.8726	172	-0.34	0.7368
**Genotype*Dose**		DM	0.1		RY	0.1	-37.3138	26.9164	173	-1.39	0.1674
**Genotype*Dose**		DM	0.1		RY	1	-56.6873	26.8761	172	-2.11	0.0364
**Genotype*Dose**		DM	0.1		RY	6	-10.7062	26.9995	175	-0.40	0.6922
**Genotype*Dose**		DM	0.1		RY	0	1.3471	26.8944	173	0.05	0.9601
**Genotype*Dose**		DM	0.1		WT	0.1	-17.0895	26.8944	173	-0.64	0.5260
**Genotype*Dose**		DM	0.1		WT	1	-18.1349	26.9771	175	-0.67	0.5023
**Genotype*Dose**		DM	0.1		WT	6	-44.4690	26.9164	173	-1.65	0.1003
**Genotype*Dose**		DM	0.1		WT	0	2.7241	26.8944	173	0.10	0.9194
**Genotype*Dose**		DM	1		DM	6	87.1416	26.9879	175	3.23	0.0015
**Genotype*Dose**		DM	1		DM	0	62.0670	26.8726	172	2.31	0.0221
**Genotype*Dose**		DM	1		RY	0.1	33.7981	26.9164	173	1.26	0.2109
**Genotype*Dose**		DM	1		RY	1	14.4246	26.8761	172	0.54	0.5922
**Genotype*Dose**		DM	1		RY	6	60.4058	26.9995	175	2.24	0.0265
**Genotype*Dose**		DM	1		RY	0	72.4591	26.8944	173	2.69	0.0078
**Genotype*Dose**		DM	1		WT	0.1	54.0225	26.8944	173	2.01	0.0461
**Genotype*Dose**		DM	1		WT	1	52.9771	26.9771	175	1.96	0.0511
**Genotype*Dose**		DM	1		WT	6	26.6429	26.9164	173	0.99	0.3236
**Genotype*Dose**		DM	1		WT	0	73.8361	26.8944	173	2.75	0.0067
**Genotype*Dose**		DM	6		DM	0	-25.0746	26.9661	174	-0.93	0.3537
**Genotype*Dose**		DM	6		RY	0.1	-53.3435	27.0098	176	-1.97	0.0498

Mixed model for AUC proximal

The Mixed Procedure

Differences of Least Squares Means											
**Effect**	**Sex**	**Genotype**	**Dose**	**Sex**	**Genotype**	**Dose**	**Estimate**	**Standard Error**	**DF**	**t Value**	**Pr > |t|**
**Genotype*Dose**		DM	6		RY	1	-72.7170	26.9697	175	-2.70	0.0077
**Genotype*Dose**		DM	6		RY	6	-26.7358	27.0928	177	-0.99	0.3251
**Genotype*Dose**		DM	6		RY	0	-14.6825	26.9879	175	-0.54	0.5871
**Genotype*Dose**		DM	6		WT	0.1	-33.1191	26.9879	175	-1.23	0.2214
**Genotype*Dose**		DM	6		WT	1	-34.1645	27.0703	177	-1.26	0.2086
**Genotype*Dose**		DM	6		WT	6	-60.4987	27.0098	176	-2.24	0.0264
**Genotype*Dose**		DM	6		WT	0	-13.3055	26.9879	175	-0.49	0.6226
**Genotype*Dose**		DM	0		RY	0.1	-28.2688	26.8945	173	-1.05	0.2947
**Genotype*Dose**		DM	0		RY	1	-47.6424	26.8543	172	-1.77	0.0778
**Genotype*Dose**		DM	0		RY	6	-1.6612	26.9778	175	-0.06	0.9510
**Genotype*Dose**		DM	0		RY	0	10.3921	26.8726	172	0.39	0.6994
**Genotype*Dose**		DM	0		WT	0.1	-8.0445	26.8726	172	-0.30	0.7650
**Genotype*Dose**		DM	0		WT	1	-9.0899	26.9553	174	-0.34	0.7364
**Genotype*Dose**		DM	0		WT	6	-35.4241	26.8945	173	-1.32	0.1895
**Genotype*Dose**		DM	0		WT	0	11.7691	26.8726	172	0.44	0.6620
**Genotype*Dose**		RY	0.1		RY	1	-19.3735	26.8981	173	-0.72	0.4723
**Genotype*Dose**		RY	0.1		RY	6	26.6076	27.0215	176	0.98	0.3261
**Genotype*Dose**		RY	0.1		RY	0	38.6609	26.9164	173	1.44	0.1527
**Genotype*Dose**		RY	0.1		WT	0.1	20.2243	26.9164	173	0.75	0.4534
**Genotype*Dose**		RY	0.1		WT	1	19.1789	26.9990	175	0.71	0.4784
**Genotype*Dose**		RY	0.1		WT	6	-7.1552	26.9383	174	-0.27	0.7909
**Genotype*Dose**		RY	0.1		WT	0	40.0379	26.9164	173	1.49	0.1387
**Genotype*Dose**		RY	1		RY	6	45.9812	26.9814	175	1.70	0.0901
**Genotype*Dose**		RY	1		RY	0	58.0345	26.8761	172	2.16	0.0322
**Genotype*Dose**		RY	1		WT	0.1	39.5979	26.8761	172	1.47	0.1425
**Genotype*Dose**		RY	1		WT	1	38.5525	26.9589	174	1.43	0.1545
**Genotype*Dose**		RY	1		WT	6	12.2183	26.8981	173	0.45	0.6502
**Genotype*Dose**		RY	1		WT	0	59.4115	26.8761	172	2.21	0.0284
**Genotype*Dose**		RY	6		RY	0	12.0533	26.9996	175	0.45	0.6558
**Genotype*Dose**		RY	6		WT	0.1	-6.3833	26.9996	175	-0.24	0.8134
**Genotype*Dose**		RY	6		WT	1	-7.4287	27.0820	177	-0.27	0.7842
**Genotype*Dose**		RY	6		WT	6	-33.7628	27.0215	176	-1.25	0.2132

Mixed model for AUC proximal

The Mixed Procedure

Differences of Least Squares Means											
**Effect**	**Sex**	**Genotype**	**Dose**	**Sex**	**Genotype**	**Dose**	**Estimate**	**Standard Error**	**DF**	**t Value**	**Pr > |t|**
**Genotype*Dose**		RY	6		WT	0	13.4303	26.9995	175	0.50	0.6195
**Genotype*Dose**		RY	0		WT	0.1	-18.4366	26.8944	173	-0.69	0.4939
**Genotype*Dose**		RY	0		WT	1	-19.4820	26.9771	175	-0.72	0.4712
**Genotype*Dose**		RY	0		WT	6	-45.8161	26.9164	173	-1.70	0.0905
**Genotype*Dose**		RY	0		WT	0	1.3770	26.8944	173	0.05	0.9592
**Genotype*Dose**		WT	0.1		WT	1	-1.0454	26.9771	175	-0.04	0.9691
**Genotype*Dose**		WT	0.1		WT	6	-27.3796	26.9164	173	-1.02	0.3105
**Genotype*Dose**		WT	0.1		WT	0	19.8136	26.8944	173	0.74	0.4623
**Genotype*Dose**		WT	1		WT	6	-26.3342	26.9990	175	-0.98	0.3307
**Genotype*Dose**		WT	1		WT	0	20.8590	26.9771	175	0.77	0.4404
**Genotype*Dose**		WT	6		WT	0	47.1932	26.9164	173	1.75	0.0813

Mixed model for AUC distal

The Mixed Procedure

Solution for Fixed Effects											
**Effect**	**Sex**	**Genotype**	**Dose**	**Estimate**	**Standard Error**	**DF**	**t Value**	**Pr > |t|**	**Alpha**	**Lower**	**Upper**
**Intercept**				1.6447	0.06079	171	27.05	<.0001	0.05	1.5247	1.7647
**Sex**	F			0.02811	0.02953	171	0.95	0.3425	0.05	-0.03018	0.08640
**Sex**	M			0	.	.	.	.	.	.	.
**Genotype**		CG		0.1772	0.08327	169	2.13	0.0348	0.05	0.01282	0.3416
**Genotype**		DM		0.01628	0.08327	169	0.20	0.8452	0.05	-0.1481	0.1807
**Genotype**		RY		-0.00320	0.08337	170	-0.04	0.9694	0.05	-0.1678	0.1614
**Genotype**		WT		0	.	.	.	.	.	.	.
**Dose**			0.1	0.1052	0.08337	170	1.26	0.2089	0.05	-0.05940	0.2698
**Dose**			1	-0.00309	0.08376	173	-0.04	0.9706	0.05	-0.1684	0.1622
**Dose**			6	0.1088	0.08348	171	1.30	0.1942	0.05	-0.05597	0.2736
**Dose**			0	0	.	.	.	.	.	.	.
**Genotype*Dose**		CG	0.1	-0.1809	0.1178	169	-1.54	0.1264	0.05	-0.4133	0.05159
**Genotype*Dose**		CG	1	-0.05611	0.1181	171	-0.48	0.6353	0.05	-0.2892	0.1770
**Genotype*Dose**		CG	6	-0.2947	0.1180	171	-2.50	0.0135	0.05	-0.5276	-0.06177
**Genotype*Dose**		CG	0	0	.	.	.	.	.	.	.
**Genotype*Dose**		DM	0.1	-0.1123	0.1178	170	-0.95	0.3421	0.05	-0.3449	0.1203
**Genotype*Dose**		DM	1	0.1924	0.1181	171	1.63	0.1052	0.05	-0.04076	0.4255
**Genotype*Dose**		DM	6	-0.1129	0.1182	172	-0.96	0.3409	0.05	-0.3463	0.1205
**Genotype*Dose**		DM	0	0	.	.	.	.	.	.	.
**Genotype*Dose**		RY	0.1	0.04504	0.1180	171	0.38	0.7031	0.05	-0.1879	0.2779
**Genotype*Dose**		RY	1	0.1175	0.1181	171	1.00	0.3211	0.05	-0.1156	0.3507
**Genotype*Dose**		RY	6	-0.06624	0.1183	172	-0.56	0.5764	0.05	-0.2998	0.1673
**Genotype*Dose**		RY	0	0	.	.	.	.	.	.	.
**Genotype*Dose**		WT	0.1	0	.	.	.	.	.	.	.
**Genotype*Dose**		WT	1	0	.	.	.	.	.	.	.
**Genotype*Dose**		WT	6	0	.	.	.	.	.	.	.
**Genotype*Dose**		WT	0	0	.	.	.	.	.	.	.

Mixed model for AUC distal

The Mixed Procedure

Type 3 Tests of Fixed Effects				
**Effect**	**Num DF**	**Den DF**	**F Value**	**Pr > F**
**Sex**	1	171	0.91	0.3425
**Genotype**	3	171	0.43	0.7321
**Dose**	3	171	1.30	0.2777
**Genotype*Dose**	9	171	1.85	0.0623

Least Squares Means					
**Effect**	**Sex**	**Genotype**	**Dose**	**Estimate**	**Standard Error**
**Sex**	F			1.7438	0.02088
**Sex**	M			1.7157	0.02088
**Genotype**		CG		1.7558	0.02946
**Genotype**		DM		1.7196	0.02954
**Genotype**		RY		1.7324	0.02957
**Genotype**		WT		1.7115	0.02956
**Genotype*Dose**		CG	0.1	1.7603	0.05880
**Genotype*Dose**		CG	1	1.7767	0.05895
**Genotype*Dose**		CG	6	1.6501	0.05911
**Genotype*Dose**		CG	0	1.8359	0.05880
**Genotype*Dose**		DM	0.1	1.6679	0.05895
**Genotype*Dose**		DM	1	1.8643	0.05895
**Genotype*Dose**		DM	6	1.6709	0.05960
**Genotype*Dose**		DM	0	1.6750	0.05880
**Genotype*Dose**		RY	0.1	1.8058	0.05911
**Genotype*Dose**		RY	1	1.7700	0.05882
**Genotype*Dose**		RY	6	1.6981	0.05966
**Genotype*Dose**		RY	0	1.6556	0.05895
**Genotype*Dose**		WT	0.1	1.7639	0.05895
**Genotype*Dose**		WT	1	1.6557	0.05950
**Genotype*Dose**		WT	6	1.7676	0.05911
**Genotype*Dose**		WT	0	1.6587	0.05895

Mixed model for AUC distal

The Mixed Procedure

Differences of Least Squares Means											
**Effect**	**Sex**	**Genotype**	**Dose**	**Sex**	**Genotype**	**Dose**	**Estimate**	**Standard Error**	**DF**	**t Value**	**Pr > |t|**
**Sex**	F			M			0.02811	0.02953	171	0.95	0.3425
**Genotype**		CG			DM		0.03620	0.04172	171	0.87	0.3867
**Genotype**		CG			RY		0.02340	0.04174	171	0.56	0.5758
**Genotype**		CG			WT		0.04428	0.04174	171	1.06	0.2902
**Genotype**		DM			RY		-0.01281	0.04180	172	-0.31	0.7597
**Genotype**		DM			WT		0.008078	0.04179	172	0.19	0.8470
**Genotype**		RY			WT		0.02088	0.04181	172	0.50	0.6181
**Genotype*Dose**		CG	0.1		CG	1	-0.01648	0.08327	169	-0.20	0.8433
**Genotype*Dose**		CG	0.1		CG	6	0.1102	0.08337	170	1.32	0.1882
**Genotype*Dose**		CG	0.1		CG	0	-0.07569	0.08316	169	-0.91	0.3640
**Genotype*Dose**		CG	0.1		DM	0.1	0.09231	0.08327	169	1.11	0.2692
**Genotype*Dose**		CG	0.1		DM	1	-0.1041	0.08327	169	-1.25	0.2132
**Genotype*Dose**		CG	0.1		DM	6	0.08933	0.08373	173	1.07	0.2875
**Genotype*Dose**		CG	0.1		DM	0	0.08522	0.08316	169	1.02	0.3069
**Genotype*Dose**		CG	0.1		RY	0.1	-0.04551	0.08337	170	-0.55	0.5859
**Genotype*Dose**		CG	0.1		RY	1	-0.00973	0.08317	169	-0.12	0.9070
**Genotype*Dose**		CG	0.1		RY	6	0.06213	0.08377	173	0.74	0.4593
**Genotype*Dose**		CG	0.1		RY	0	0.1047	0.08327	169	1.26	0.2103
**Genotype*Dose**		CG	0.1		WT	0.1	-0.00367	0.08327	169	-0.04	0.9649
**Genotype*Dose**		CG	0.1		WT	1	0.1046	0.08365	172	1.25	0.2129
**Genotype*Dose**		CG	0.1		WT	6	-0.00731	0.08337	170	-0.09	0.9303
**Genotype*Dose**		CG	0.1		WT	0	0.1015	0.08327	169	1.22	0.2245
**Genotype*Dose**		CG	1		CG	6	0.1267	0.08348	171	1.52	0.1311
**Genotype*Dose**		CG	1		CG	0	-0.05920	0.08327	169	-0.71	0.4781
**Genotype*Dose**		CG	1		DM	0.1	0.1088	0.08337	170	1.30	0.1937
**Genotype*Dose**		CG	1		DM	1	-0.08757	0.08337	170	-1.05	0.2951
**Genotype*Dose**		CG	1		DM	6	0.1058	0.08383	174	1.26	0.2086
**Genotype*Dose**		CG	1		DM	0	0.1017	0.08327	169	1.22	0.2236
**Genotype*Dose**		CG	1		RY	0.1	-0.02903	0.08348	171	-0.35	0.7285
**Genotype*Dose**		CG	1		RY	1	0.006750	0.08328	169	0.08	0.9355
**Genotype*Dose**		CG	1		RY	6	0.07862	0.08387	174	0.94	0.3499
**Genotype*Dose**		CG	1		RY	0	0.1212	0.08337	170	1.45	0.1479

Mixed model for AUC distal

The Mixed Procedure

Differences of Least Squares Means											
**Effect**	**Sex**	**Genotype**	**Dose**	**Sex**	**Genotype**	**Dose**	**Estimate**	**Standard Error**	**DF**	**t Value**	**Pr > |t|**
**Genotype*Dose**		CG	1		WT	0.1	0.01281	0.08337	170	0.15	0.8780
**Genotype*Dose**		CG	1		WT	1	0.1211	0.08376	173	1.45	0.1501
**Genotype*Dose**		CG	1		WT	6	0.009176	0.08348	171	0.11	0.9126
**Genotype*Dose**		CG	1		WT	0	0.1180	0.08337	170	1.42	0.1588
**Genotype*Dose**		CG	6		CG	0	-0.1859	0.08337	170	-2.23	0.0271
**Genotype*Dose**		CG	6		DM	0.1	-0.01786	0.08348	171	-0.21	0.8309
**Genotype*Dose**		CG	6		DM	1	-0.2142	0.08348	171	-2.57	0.0111
**Genotype*Dose**		CG	6		DM	6	-0.02084	0.08394	175	-0.25	0.8043
**Genotype*Dose**		CG	6		DM	0	-0.02494	0.08337	170	-0.30	0.7652
**Genotype*Dose**		CG	6		RY	0.1	-0.1557	0.08359	172	-1.86	0.0643
**Genotype*Dose**		CG	6		RY	1	-0.1199	0.08339	170	-1.44	0.1523
**Genotype*Dose**		CG	6		RY	6	-0.04803	0.08398	175	-0.57	0.5681
**Genotype*Dose**		CG	6		RY	0	-0.00546	0.08348	171	-0.07	0.9479
**Genotype*Dose**		CG	6		WT	0.1	-0.1138	0.08348	171	-1.36	0.1745
**Genotype*Dose**		CG	6		WT	1	-0.00557	0.08387	174	-0.07	0.9471
**Genotype*Dose**		CG	6		WT	6	-0.1175	0.08359	172	-1.41	0.1617
**Genotype*Dose**		CG	6		WT	0	-0.00866	0.08348	171	-0.10	0.9175
**Genotype*Dose**		CG	0		DM	0.1	0.1680	0.08327	169	2.02	0.0452
**Genotype*Dose**		CG	0		DM	1	-0.02837	0.08327	169	-0.34	0.7338
**Genotype*Dose**		CG	0		DM	6	0.1650	0.08373	173	1.97	0.0503
**Genotype*Dose**		CG	0		DM	0	0.1609	0.08316	169	1.94	0.0547
**Genotype*Dose**		CG	0		RY	0.1	0.03017	0.08337	170	0.36	0.7179
**Genotype*Dose**		CG	0		RY	1	0.06595	0.08317	169	0.79	0.4289
**Genotype*Dose**		CG	0		RY	6	0.1378	0.08377	173	1.65	0.1017
**Genotype*Dose**		CG	0		RY	0	0.1804	0.08327	169	2.17	0.0317
**Genotype*Dose**		CG	0		WT	0.1	0.07202	0.08327	169	0.86	0.3883
**Genotype*Dose**		CG	0		WT	1	0.1803	0.08365	172	2.16	0.0325
**Genotype*Dose**		CG	0		WT	6	0.06838	0.08337	170	0.82	0.4133
**Genotype*Dose**		CG	0		WT	0	0.1772	0.08327	169	2.13	0.0348
**Genotype*Dose**		DM	0.1		DM	1	-0.1964	0.08337	170	-2.36	0.0197
**Genotype*Dose**		DM	0.1		DM	6	-0.00298	0.08383	174	-0.04	0.9717
**Genotype*Dose**		DM	0.1		DM	0	-0.00709	0.08327	169	-0.09	0.9323

Mixed model for AUC distal

The Mixed Procedure

Differences of Least Squares Means											
Effect	Sex	Genotype	Dose	Sex	Genotype	Dose	Estimate	Standard Error	DF	t Value	Pr > |t|
Genotype*Dose		DM	0.1		RY	0.1	-0.1378	0.08348	171	-1.65	0.1006
Genotype*Dose		DM	0.1		RY	1	-0.1020	0.08328	169	-1.23	0.2221
Genotype*Dose		DM	0.1		RY	6	-0.03018	0.08387	174	-0.36	0.7194
Genotype*Dose		DM	0.1		RY	0	0.01239	0.08337	170	0.15	0.8820
Genotype*Dose		DM	0.1		WT	0.1	-0.09598	0.08337	170	-1.15	0.2513
Genotype*Dose		DM	0.1		WT	1	0.01229	0.08376	173	0.15	0.8835
Genotype*Dose		DM	0.1		WT	6	-0.09962	0.08348	171	-1.19	0.2344
Genotype*Dose		DM	0.1		WT	0	0.009195	0.08337	170	0.11	0.9123
Genotype*Dose		DM	1		DM	6	0.1934	0.08383	174	2.31	0.0222
Genotype*Dose		DM	1		DM	0	0.1893	0.08327	169	2.27	0.0243
Genotype*Dose		DM	1		RY	0.1	0.05854	0.08348	171	0.70	0.4841
Genotype*Dose		DM	1		RY	1	0.09432	0.08328	169	1.13	0.2590
Genotype*Dose		DM	1		RY	6	0.1662	0.08387	174	1.98	0.0491
Genotype*Dose		DM	1		RY	0	0.2088	0.08337	170	2.50	0.0132
Genotype*Dose		DM	1		WT	0.1	0.1004	0.08337	170	1.20	0.2303
Genotype*Dose		DM	1		WT	1	0.2087	0.08376	173	2.49	0.0137
Genotype*Dose		DM	1		WT	6	0.09674	0.08348	171	1.16	0.2481
Genotype*Dose		DM	1		WT	0	0.2056	0.08337	170	2.47	0.0147
Genotype*Dose		DM	6		DM	0	-0.00411	0.08373	173	-0.05	0.9609
Genotype*Dose		DM	6		RY	0.1	-0.1348	0.08394	175	-1.61	0.1100
Genotype*Dose		DM	6		RY	1	-0.09907	0.08374	173	-1.18	0.2384
Genotype*Dose		DM	6		RY	6	-0.02720	0.08433	177	-0.32	0.7474
Genotype*Dose		DM	6		RY	0	0.01537	0.08383	174	0.18	0.8547
Genotype*Dose		DM	6		WT	0.1	-0.09300	0.08383	174	-1.11	0.2688
Genotype*Dose		DM	6		WT	1	0.01527	0.08422	176	0.18	0.8564
Genotype*Dose		DM	6		WT	6	-0.09664	0.08394	175	-1.15	0.2512
Genotype*Dose		DM	6		WT	0	0.01217	0.08383	174	0.15	0.8847
Genotype*Dose		DM	0		RY	0.1	-0.1307	0.08337	170	-1.57	0.1187
Genotype*Dose		DM	0		RY	1	-0.09496	0.08317	169	-1.14	0.2552
Genotype*Dose		DM	0		RY	6	-0.02309	0.08377	173	-0.28	0.7831
Genotype*Dose		DM	0		RY	0	0.01948	0.08327	169	0.23	0.8153
Genotype*Dose		DM	0		WT	0.1	-0.08889	0.08327	169	-1.07	0.2872

Mixed model for AUC distal

The Mixed Procedure

Differences of Least Squares Means											
Effect	Sex	Genotype	Dose	Sex	Genotype	Dose	Estimate	Standard Error	DF	t Value	Pr > |t|
**Genotype*Dose**		DM	0		WT	1	0.01937	0.08365	172	0.23	0.8171
**Genotype*Dose**		DM	0		WT	6	-0.09253	0.08337	170	-1.11	0.2686
**Genotype*Dose**		DM	0		WT	0	0.01628	0.08327	169	0.20	0.8452
**Genotype*Dose**		RY	0.1		RY	1	0.03578	0.08339	170	0.43	0.6684
**Genotype*Dose**		RY	0.1		RY	6	0.1076	0.08398	175	1.28	0.2016
**Genotype*Dose**		RY	0.1		RY	0	0.1502	0.08348	171	1.80	0.0737
**Genotype*Dose**		RY	0.1		WT	0.1	0.04184	0.08348	171	0.50	0.6169
**Genotype*Dose**		RY	0.1		WT	1	0.1501	0.08387	174	1.79	0.0752
**Genotype*Dose**		RY	0.1		WT	6	0.03820	0.08359	172	0.46	0.6482
**Genotype*Dose**		RY	0.1		WT	0	0.1470	0.08348	171	1.76	0.0800
**Genotype*Dose**		RY	1		RY	6	0.07187	0.08378	173	0.86	0.3922
**Genotype*Dose**		RY	1		RY	0	0.1144	0.08328	169	1.37	0.1712
**Genotype*Dose**		RY	1		WT	0.1	0.006065	0.08328	169	0.07	0.9420
**Genotype*Dose**		RY	1		WT	1	0.1143	0.08367	172	1.37	0.1736
**Genotype*Dose**		RY	1		WT	6	0.002426	0.08339	170	0.03	0.9768
**Genotype*Dose**		RY	1		WT	0	0.1112	0.08328	169	1.34	0.1834
**Genotype*Dose**		RY	6		RY	0	0.04257	0.08387	174	0.51	0.6124
**Genotype*Dose**		RY	6		WT	0.1	-0.06580	0.08387	174	-0.78	0.4338
**Genotype*Dose**		RY	6		WT	1	0.04247	0.08426	176	0.50	0.6149
**Genotype*Dose**		RY	6		WT	6	-0.06944	0.08398	175	-0.83	0.4094
**Genotype*Dose**		RY	6		WT	0	0.03937	0.08387	174	0.47	0.6394
**Genotype*Dose**		RY	0		WT	0.1	-0.1084	0.08337	170	-1.30	0.1954
**Genotype*Dose**		RY	0		WT	1	-0.00010	0.08376	173	-0.00	0.9990
**Genotype*Dose**		RY	0		WT	6	-0.1120	0.08348	171	-1.34	0.1815
**Genotype*Dose**		RY	0		WT	0	-0.00320	0.08337	170	-0.04	0.9694
**Genotype*Dose**		WT	0.1		WT	1	0.1083	0.08376	173	1.29	0.1979
**Genotype*Dose**		WT	0.1		WT	6	-0.00364	0.08348	171	-0.04	0.9653
**Genotype*Dose**		WT	0.1		WT	0	0.1052	0.08337	170	1.26	0.2089
**Genotype*Dose**		WT	1		WT	6	-0.1119	0.08387	174	-1.33	0.1838
**Genotype*Dose**		WT	1		WT	0	-0.00309	0.08376	173	-0.04	0.9706
**Genotype*Dose**		WT	6		WT	0	0.1088	0.08348	171	1.30	0.1942

Mixed Effects Model for Total Dendrites

The Mixed Procedure

Solution for Fixed Effects											
**Effect**	**Sex**	**Genotype**	**Dose**	**Estimate**	**Standard Error**	**DF**	**t Value**	**Pr > |t|**	**Alpha**	**Lower**	**Upper**
**Intercept**				6.0913	0.1252	181	48.65	<.0001	0.05	5.8443	6.3384
**Sex**	F			0.03278	0.08868	182	0.37	0.7121	0.05	-0.1422	0.2077
**Sex**	M			0	.	.	.	.	.	.	.
**Genotype**		CG		-0.03414	0.1253	182	-0.27	0.7856	0.05	-0.2814	0.2131
**Genotype**		DM		0.002304	0.1255	183	0.02	0.9854	0.05	-0.2453	0.2499
**Genotype**		RY		0.03738	0.1256	183	0.30	0.7663	0.05	-0.2104	0.2852
**Genotype**		WT		0	.	.	.	.	.	.	.
**Dose**			0.1	0.1224	0.1250	181	0.98	0.3289	0.05	-0.1243	0.3691
**Dose**			1	0.3725	0.1251	181	2.98	0.0033	0.05	0.1256	0.6195
**Dose**			6	0.01821	0.1256	183	0.15	0.8848	0.05	-0.2295	0.2660
**Dose**			0	0	.	.	.	.	.	.	.

Type 3 Tests of Fixed Effects				
**Effect**	**Num DF**	**Den DF**	**F Value**	**Pr > F**
**Sex**	1	182	0.14	0.7121
**Genotype**	3	182	0.11	0.9549
**Dose**	3	182	3.74	0.0122

Least Squares Means					
**Effect**	**Sex**	**Genotype**	**Dose**	**Estimate**	**Standard Error**
**Sex**	F			6.2538	0.06270
**Sex**	M			6.2210	0.06271
**Genotype**		CG		6.2019	0.08840
**Genotype**		DM		6.2383	0.08871
**Genotype**		RY		6.2734	0.08881
**Genotype**		WT		6.2360	0.08880
**Dose**			0.1	6.2315	0.08848
**Dose**			1	6.4816	0.08866

Mixed Effects Model for Total Dendrites

The Mixed Procedure

Least Squares Means					
**Effect**	**Sex**	**Genotype**	**Dose**	**Estimate**	**Standard Error**
**Dose**			6	6.1273	0.08925
**Dose**			0	6.1091	0.08833

Differences of Least Squares Means											
**Effect**	**Sex**	**Genotype**	**Dose**	**Sex**	**Genotype**	**Dose**	**Estimate**	**Standard Error**	**DF**	**t Value**	**Pr > |t|**
**Sex**	F			M			0.03278	0.08868	182	0.37	0.7121
**Genotype**		CG			DM		-0.03645	0.1252	182	-0.29	0.7714
**Genotype**		CG			RY		-0.07152	0.1253	182	-0.57	0.5688
**Genotype**		CG			WT		-0.03414	0.1253	182	-0.27	0.7856
**Genotype**		DM			RY		-0.03508	0.1255	183	-0.28	0.7802
**Genotype**		DM			WT		0.002304	0.1255	183	0.02	0.9854
**Genotype**		RY			WT		0.03738	0.1256	183	0.30	0.7663
**Dose**			0.1			1	-0.2501	0.1253	182	-2.00	0.0473
**Dose**			0.1			6	0.1042	0.1257	184	0.83	0.4082
**Dose**			0.1			0	0.1224	0.1250	181	0.98	0.3289
**Dose**			1			6	0.3543	0.1258	184	2.82	0.0054
**Dose**			1			0	0.3725	0.1251	181	2.98	0.0033
**Dose**			6			0	0.01821	0.1256	183	0.15	0.8848

Mixed Effects Model for terminal tips

The Mixed Procedure

Solution for Fixed Effects											
**Effect**	**Sex**	**Genotype**	**Dose**	**Estimate**	**Standard Error**	**DF**	**t Value**	**Pr > |t|**	**Alpha**	**Lower**	**Upper**
**Intercept**				11.5335	0.3202	183	36.02	<.0001	0.05	10.9018	12.1653
**Sex**	F			0.2739	0.2266	183	1.21	0.2284	0.05	-0.1733	0.7210
**Sex**	M			0	.	.	.	.	.	.	.
**Genotype**		CG		0.2369	0.3204	183	0.74	0.4605	0.05	-0.3952	0.8690
**Genotype**		DM		-0.06983	0.3207	184	-0.22	0.8279	0.05	-0.7025	0.5628
**Genotype**		RY		0.04204	0.3208	184	0.13	0.8959	0.05	-0.5908	0.6749
**Genotype**		WT		0	.	.	.	.	.	.	.
**Dose**			0.1	-0.00265	0.3199	182	-0.01	0.9934	0.05	-0.6339	0.6286
**Dose**			1	0.9588	0.3201	182	3.00	0.0031	0.05	0.3272	1.5905
**Dose**			6	-0.3060	0.3207	184	-0.95	0.3412	0.05	-0.9388	0.3268
**Dose**			0	0	.	.	.	.	.	.	.

Type 3 Tests of Fixed Effects				
**Effect**	**Num DF**	**Den DF**	**F Value**	**Pr > F**
**Sex**	1	183	1.46	0.2284
**Genotype**	3	183	0.34	0.7985
**Dose**	3	183	5.88	0.0007

Least Squares Means					
**Effect**	**Sex**	**Genotype**	**Dose**	**Estimate**	**Standard Error**
**Sex**	F			12.0223	0.1603
**Sex**	M			11.7484	0.1603
**Genotype**		CG		12.0699	0.2262
**Genotype**		DM		11.7632	0.2267
**Genotype**		RY		11.8751	0.2268
**Genotype**		WT		11.8330	0.2268
**Dose**			0.1	11.7201	0.2263
**Dose**			1	12.6816	0.2266

Mixed Effects Model for terminal tips

The Mixed Procedure

Least Squares Means					
**Effect**	**Sex**	**Genotype**	**Dose**	**Estimate**	**Standard Error**
**Dose**			6	11.4167	0.2275
**Dose**			0	11.7228	0.2261

Differences of Least Squares Means											
**Effect**	**Sex**	**Genotype**	**Dose**	**Sex**	**Genotype**	**Dose**	**Estimate**	**Standard Error**	**DF**	**t Value**	**Pr > |t|**
**Sex**	F			M			0.2739	0.2266	183	1.21	0.2284
**Genotype**		CG			DM		0.3067	0.3202	183	0.96	0.3394
**Genotype**		CG			RY		0.1949	0.3204	183	0.61	0.5437
**Genotype**		CG			WT		0.2369	0.3204	183	0.74	0.4605
**Genotype**		DM			RY		-0.1119	0.3207	184	-0.35	0.7276
**Genotype**		DM			WT		-0.06983	0.3207	184	-0.22	0.8279
**Genotype**		RY			WT		0.04204	0.3208	184	0.13	0.8959
**Dose**			0.1			1	-0.9615	0.3203	183	-3.00	0.0031
**Dose**			0.1			6	0.3034	0.3209	184	0.95	0.3456
**Dose**			0.1			0	-0.00265	0.3199	182	-0.01	0.9934
**Dose**			1			6	1.2649	0.3211	184	3.94	0.0001
**Dose**			1			0	0.9588	0.3201	182	3.00	0.0031
**Dose**			6			0	-0.3060	0.3207	184	-0.95	0.3412

Mixed Effects Model for dendrite length sum

The Mixed Procedure

Solution for Fixed Effects										
**Effect**	**Sex**	**Genotype**	**Dose**	**Estimate**	**Standard Error**	**DF**	**t Value**	**Pr > |t|**	**Alpha**	**Lower**	**Upper**
**Intercept**				587.25	36.6338	173	16.03	<.0001	0.05	514.94	659.55
**Sex**	F			9.2600	17.7872	174	0.52	0.6033	0.05	-25.8470	44.3671
**Sex**	M			0	.	.	.	.	.	.	.
**Genotype**		CG		128.08	50.2063	172	2.55	0.0116	0.05	28.9778	227.18
**Genotype**		DM		22.9017	50.2063	172	0.46	0.6489	0.05	-76.1972	122.00
**Genotype**		RY		-5.7241	50.2477	173	-0.11	0.9094	0.05	-104.90	93.4543
**Genotype**		WT		0	.	.	.	.	.	.	.
**Dose**			0.1	52.0065	50.2477	173	1.04	0.3021	0.05	-47.1720	151.18
**Dose**			1	42.7621	50.4042	175	0.85	0.3974	0.05	-56.7175	142.24
**Dose**			6	74.2940	50.2893	173	1.48	0.1414	0.05	-24.9643	173.55
**Dose**			0	0	.	.	.	.	.	.	.
**Genotype*Dose**		CG	0.1	-116.54	71.0025	172	-1.64	0.1025	0.05	-256.69	23.6052
**Genotype*Dose**		CG	1	-80.7242	71.1425	173	-1.13	0.2581	0.05	-221.14	59.6923
**Genotype*Dose**		CG	6	-215.30	71.0906	173	-3.03	0.0028	0.05	-355.61	-74.9805
**Genotype*Dose**		CG	0	0	.	.	.	.	.	.	.
**Genotype*Dose**		DM	0.1	-67.8833	71.0318	173	-0.96	0.3406	0.05	-208.09	72.3201
**Genotype*Dose**		DM	1	96.1550	71.1426	173	1.35	0.1783	0.05	-44.2616	236.57
**Genotype*Dose**		DM	6	-104.93	71.1865	174	-1.47	0.1423	0.05	-245.43	35.5751
**Genotype*Dose**		DM	0	0	.	.	.	.	.	.	.
**Genotype*Dose**		RY	0.1	34.5311	71.0905	173	0.49	0.6278	0.05	-105.78	174.85
**Genotype*Dose**		RY	1	68.3232	71.1473	173	0.96	0.3382	0.05	-72.1026	208.75
**Genotype*Dose**		RY	6	-44.0223	71.2313	174	-0.62	0.5374	0.05	-184.61	96.5646
**Genotype*Dose**		RY	0	0	.	.	.	.	.	.	.
**Genotype*Dose**		WT	0.1	0	.	.	.	.	.	.	.
**Genotype*Dose**		WT	1	0	.	.	.	.	.	.	.
**Genotype*Dose**		WT	6	0	.	.	.	.	.	.	.
**Genotype*Dose**		WT	0	0	.	.	.	.	.	.	.

Mixed Effects Model for dendrite length sum

The Mixed Procedure

Type 3 Tests of Fixed Effects				
Effect	Num DF	Den DF	F Value	Pr > F
Sex	1	174	0.27	0.6033
Genotype	3	174	0.38	0.7661
Dose	3	174	3.79	0.0114
Genotype*Dose	9	174	2.22	0.0229

Least Squares Means					
Effect	Sex	Genotype	Dose	Estimate	Standard Error
Sex	F			648.19	12.5775
Sex	M			638.93	12.5774
Genotype		CG		659.08	17.7580
Genotype		DM		637.88	17.7893
Genotype		RY		643.12	17.8017
Genotype		WT		634.14	17.8003
Dose			0.1	642.72	17.7653
Dose			1	691.89	17.7868
Dose			6	611.42	17.8464
Dose			0	628.19	17.7506
Genotype*Dose		CG	0.1	655.42	35.4720
Genotype*Dose		CG	1	681.99	35.5305
Genotype*Dose		CG	6	578.95	35.5893
Genotype*Dose		CG	0	719.95	35.4720
Genotype*Dose		DM	0.1	598.90	35.5305
Genotype*Dose		DM	1	753.69	35.5305
Genotype*Dose		DM	6	584.15	35.7805
Genotype*Dose		DM	0	614.78	35.4720
Genotype*Dose		RY	0.1	672.69	35.5893
Genotype*Dose		RY	1	697.24	35.4815
Genotype*Dose		RY	6	616.42	35.8114
Genotype*Dose		RY	0	586.15	35.5305
Genotype*Dose		WT	0.1	643.88	35.5305
Genotype*Dose		WT	1	634.64	35.7514

Mixed Effects Model for dendrite length sum

The Mixed Procedure

Least Squares Means					
**Effect**	**Sex**	**Genotype**	**Dose**	**Estimate**	**Standard Error**
**Genotype*Dose**		WT	6	666.17	35.5893
**Genotype*Dose**		WT	0	591.88	35.5305

Differences of Least Squares Means											
**Effect**	**Sex**	**Genotype**	**Dose**	**Sex**	**Genotype**	**Dose**	**Estimate**	**Standard Error**	**DF**	**t Value**	**Pr > |t|**
**Sex**	F			M			9.2600	17.7872	174	0.52	0.6033
**Genotype**		CG			DM		21.1977	25.1357	173	0.84	0.4002
**Genotype**		CG			RY		15.9521	25.1445	173	0.63	0.5266
**Genotype**		CG			WT		24.9360	25.1435	173	0.99	0.3227
**Genotype**		DM			RY		-5.2456	25.1666	174	-0.21	0.8351
**Genotype**		DM			WT		3.7383	25.1656	174	0.15	0.8821
**Genotype**		RY			WT		8.9839	25.1744	174	0.36	0.7216
**Dose**			0.1			1	-49.1677	25.1391	173	-1.96	0.0521
**Dose**			0.1			6	31.3001	25.1813	174	1.24	0.2155
**Dose**			0.1			0	14.5330	25.1135	173	0.58	0.5636
**Dose**			1			6	80.4677	25.1965	175	3.19	0.0017
**Dose**			1			0	63.7006	25.1288	173	2.53	0.0121
**Dose**			6			0	-16.7671	25.1710	174	-0.67	0.5062
**Genotype*Dose**		CG	0.1		CG	1	-26.5734	50.2063	172	-0.53	0.5973
**Genotype*Dose**		CG	0.1		CG	6	76.4673	50.2480	173	1.52	0.1299
**Genotype*Dose**		CG	0.1		CG	0	-64.5354	50.1649	172	-1.29	0.2000
**Genotype*Dose**		CG	0.1		DM	0.1	56.5164	50.2063	172	1.13	0.2619
**Genotype*Dose**		CG	0.1		DM	1	-98.2776	50.2063	172	-1.96	0.0519
**Genotype*Dose**		CG	0.1		DM	6	71.2709	50.3836	175	1.41	0.1590
**Genotype*Dose**		CG	0.1		DM	0	40.6396	50.1649	172	0.81	0.4190
**Genotype*Dose**		CG	0.1		RY	0.1	-17.2722	50.2480	173	-0.34	0.7315
**Genotype*Dose**		CG	0.1		RY	1	-41.8199	50.1717	172	-0.83	0.4057
**Genotype*Dose**		CG	0.1		RY	6	38.9937	50.4055	175	0.77	0.4402
**Genotype*Dose**		CG	0.1		RY	0	69.2654	50.2063	172	1.38	0.1695
**Genotype*Dose**		CG	0.1		WT	0.1	11.5348	50.2063	172	0.23	0.8186
**Genotype*Dose**		CG	0.1		WT	1	20.7792	50.3629	174	0.41	0.6804

Mixed Effects Model for dendrite length sum

The Mixed Procedure

Differences of Least Squares Means											
Effect	Sex	Genotype	Dose	Sex	Genotype	Dose	Estimate	Standard Error	DF	t Value	Pr > |t|
**Genotype*Dose**		CG	0.1		WT	6	-10.7527	50.2480	173	-0.21	0.8308
**Genotype*Dose**		CG	0.1		WT	0	63.5413	50.2063	172	1.27	0.2074
**Genotype*Dose**		CG	1		CG	6	103.04	50.2893	173	2.05	0.0420
**Genotype*Dose**		CG	1		CG	0	-37.9621	50.2063	172	-0.76	0.4506
**Genotype*Dose**		CG	1		DM	0.1	83.0897	50.2477	173	1.65	0.1000
**Genotype*Dose**		CG	1		DM	1	-71.7042	50.2477	173	-1.43	0.1554
**Genotype*Dose**		CG	1		DM	6	97.8443	50.4248	175	1.94	0.0539
**Genotype*Dose**		CG	1		DM	0	67.2129	50.2063	172	1.34	0.1824
**Genotype*Dose**		CG	1		RY	0.1	9.3012	50.2893	173	0.18	0.8535
**Genotype*Dose**		CG	1		RY	1	-15.2466	50.2131	172	-0.30	0.7618
**Genotype*Dose**		CG	1		RY	6	65.5671	50.4468	175	1.30	0.1954
**Genotype*Dose**		CG	1		RY	0	95.8388	50.2477	173	1.91	0.0581
**Genotype*Dose**		CG	1		WT	0.1	38.1082	50.2477	173	0.76	0.4492
**Genotype*Dose**		CG	1		WT	1	47.3525	50.4041	175	0.94	0.3488
**Genotype*Dose**		CG	1		WT	6	15.8207	50.2893	173	0.31	0.7534
**Genotype*Dose**		CG	1		WT	0	90.1146	50.2477	173	1.79	0.0747
**Genotype*Dose**		CG	6		CG	0	-141.00	50.2480	173	-2.81	0.0056
**Genotype*Dose**		CG	6		DM	0.1	-19.9509	50.2893	173	-0.40	0.6921
**Genotype*Dose**		CG	6		DM	1	-174.74	50.2893	173	-3.47	0.0006
**Genotype*Dose**		CG	6		DM	6	-5.1964	50.4662	176	-0.10	0.9181
**Genotype*Dose**		CG	6		DM	0	-35.8277	50.2480	173	-0.71	0.4768
**Genotype*Dose**		CG	6		RY	0.1	-93.7395	50.3309	174	-1.86	0.0642
**Genotype*Dose**		CG	6		RY	1	-118.29	50.2547	173	-2.35	0.0197
**Genotype*Dose**		CG	6		RY	6	-37.4736	50.4881	176	-0.74	0.4589
**Genotype*Dose**		CG	6		RY	0	-7.2019	50.2893	173	-0.14	0.8863
**Genotype*Dose**		CG	6		WT	0.1	-64.9325	50.2893	173	-1.29	0.1984
**Genotype*Dose**		CG	6		WT	1	-55.6882	50.4456	175	-1.10	0.2711
**Genotype*Dose**		CG	6		WT	6	-87.2200	50.3309	174	-1.73	0.0849
**Genotype*Dose**		CG	6		WT	0	-12.9260	50.2893	173	-0.26	0.7975
**Genotype*Dose**		CG	0		DM	0.1	121.05	50.2063	172	2.41	0.0170
**Genotype*Dose**		CG	0		DM	1	-33.7421	50.2063	172	-0.67	0.5024
**Genotype*Dose**		CG	0		DM	6	135.81	50.3836	175	2.70	0.0077

Mixed Effects Model for dendrite length sum

The Mixed Procedure

Differences of Least Squares Means											
Effect	Sex	Genotype	Dose	Sex	Genotype	Dose	Estimate	Standard Error	DF	t Value	Pr > |t|
Genotype*Dose		CG	0		DM	0	105.17	50.1649	172	2.10	0.0375
Genotype*Dose		CG	0		RY	0.1	47.2632	50.2480	173	0.94	0.3482
Genotype*Dose		CG	0		RY	1	22.7155	50.1717	172	0.45	0.6513
Genotype*Dose		CG	0		RY	6	103.53	50.4055	175	2.05	0.0415
Genotype*Dose		CG	0		RY	0	133.80	50.2063	172	2.67	0.0084
Genotype*Dose		CG	0		WT	0.1	76.0702	50.2063	172	1.52	0.1316
Genotype*Dose		CG	0		WT	1	85.3146	50.3629	174	1.69	0.0921
Genotype*Dose		CG	0		WT	6	53.7827	50.2480	173	1.07	0.2860
Genotype*Dose		CG	0		WT	0	128.08	50.2063	172	2.55	0.0116
Genotype*Dose		DM	0.1		DM	1	-154.79	50.2477	173	-3.08	0.0024
Genotype*Dose		DM	0.1		DM	6	14.7545	50.4248	175	0.29	0.7702
Genotype*Dose		DM	0.1		DM	0	-15.8768	50.2063	172	-0.32	0.7522
Genotype*Dose		DM	0.1		RY	0.1	-73.7886	50.2893	173	-1.47	0.1441
Genotype*Dose		DM	0.1		RY	1	-98.3363	50.2131	172	-1.96	0.0518
Genotype*Dose		DM	0.1		RY	6	-17.5227	50.4466	175	-0.35	0.7287
Genotype*Dose		DM	0.1		RY	0	12.7490	50.2477	173	0.25	0.8000
Genotype*Dose		DM	0.1		WT	0.1	-44.9816	50.2477	173	-0.90	0.3719
Genotype*Dose		DM	0.1		WT	1	-35.7372	50.4042	175	-0.71	0.4793
Genotype*Dose		DM	0.1		WT	6	-67.2691	50.2893	173	-1.34	0.1828
Genotype*Dose		DM	0.1		WT	0	7.0249	50.2477	173	0.14	0.8890
Genotype*Dose		DM	1		DM	6	169.55	50.4248	175	3.36	0.0009
Genotype*Dose		DM	1		DM	0	138.92	50.2063	172	2.77	0.0063
Genotype*Dose		DM	1		RY	0.1	81.0054	50.2893	173	1.61	0.1090
Genotype*Dose		DM	1		RY	1	56.4576	50.2131	172	1.12	0.2624
Genotype*Dose		DM	1		RY	6	137.27	50.4466	175	2.72	0.0072
Genotype*Dose		DM	1		RY	0	167.54	50.2477	173	3.33	0.0010
Genotype*Dose		DM	1		WT	0.1	109.81	50.2477	173	2.19	0.0302
Genotype*Dose		DM	1		WT	1	119.06	50.4042	175	2.36	0.0193
Genotype*Dose		DM	1		WT	6	87.5249	50.2893	173	1.74	0.0836
Genotype*Dose		DM	1		WT	0	161.82	50.2477	173	3.22	0.0015
Genotype*Dose		DM	6		DM	0	-30.6313	50.3836	175	-0.61	0.5440
Genotype*Dose		DM	6		RY	0.1	-88.5431	50.4662	176	-1.75	0.0811

Mixed Effects Model for dendrite length sum

The Mixed Procedure

Differences of Least Squares Means											
**Effect**	**Sex**	**Genotype**	**Dose**	**Sex**	**Genotype**	**Dose**	**Estimate**	**Standard Error**	**DF**	**t Value**	**Pr > |t|**
**Genotype*Dose**		DM	6		RY	1	-113.09	50.3903	175	-2.24	0.0261
**Genotype*Dose**		DM	6		RY	6	-32.2772	50.6232	178	-0.64	0.5246
**Genotype*Dose**		DM	6		RY	0	-2.0055	50.4248	175	-0.04	0.9683
**Genotype*Dose**		DM	6		WT	0.1	-59.7361	50.4248	175	-1.18	0.2378
**Genotype*Dose**		DM	6		WT	1	-50.4918	50.5806	177	-1.00	0.3195
**Genotype*Dose**		DM	6		WT	6	-82.0236	50.4662	176	-1.63	0.1059
**Genotype*Dose**		DM	6		WT	0	-7.7297	50.4248	175	-0.15	0.8783
**Genotype*Dose**		DM	0		RY	0.1	-57.9118	50.2480	173	-1.15	0.2507
**Genotype*Dose**		DM	0		RY	1	-82.4595	50.1717	172	-1.64	0.1021
**Genotype*Dose**		DM	0		RY	6	-1.6459	50.4055	175	-0.03	0.9740
**Genotype*Dose**		DM	0		RY	0	28.6258	50.2063	172	0.57	0.5693
**Genotype*Dose**		DM	0		WT	0.1	-29.1048	50.2063	172	-0.58	0.5629
**Genotype*Dose**		DM	0		WT	1	-19.8604	50.3629	174	-0.39	0.6938
**Genotype*Dose**		DM	0		WT	6	-51.3923	50.2480	173	-1.02	0.3078
**Genotype*Dose**		DM	0		WT	0	22.9017	50.2063	172	0.46	0.6489
**Genotype*Dose**		RY	0.1		RY	1	-24.5477	50.2547	173	-0.49	0.6258
**Genotype*Dose**		RY	0.1		RY	6	56.2659	50.4881	176	1.11	0.2666
**Genotype*Dose**		RY	0.1		RY	0	86.5376	50.2893	173	1.72	0.0871
**Genotype*Dose**		RY	0.1		WT	0.1	28.8070	50.2893	173	0.57	0.5675
**Genotype*Dose**		RY	0.1		WT	1	38.0514	50.4456	175	0.75	0.4517
**Genotype*Dose**		RY	0.1		WT	6	6.5195	50.3309	174	0.13	0.8971
**Genotype*Dose**		RY	0.1		WT	0	80.8135	50.2893	173	1.61	0.1099
**Genotype*Dose**		RY	1		RY	6	80.8137	50.4122	175	1.60	0.1107
**Genotype*Dose**		RY	1		RY	0	111.09	50.2131	172	2.21	0.0283
**Genotype*Dose**		RY	1		WT	0.1	53.3548	50.2131	172	1.06	0.2895
**Genotype*Dose**		RY	1		WT	1	62.5991	50.3696	174	1.24	0.2156
**Genotype*Dose**		RY	1		WT	6	31.0673	50.2547	173	0.62	0.5373
**Genotype*Dose**		RY	1		WT	0	105.36	50.2131	172	2.10	0.0373
**Genotype*Dose**		RY	6		RY	0	30.2717	50.4468	175	0.60	0.5492
**Genotype*Dose**		RY	6		WT	0.1	-27.4589	50.4468	175	-0.54	0.5869
**Genotype*Dose**		RY	6		WT	1	-18.2146	50.6027	177	-0.36	0.7193
**Genotype*Dose**		RY	6		WT	6	-49.7464	50.4881	176	-0.99	0.3258

Mixed Effects Model for dendrite length sum

The Mixed Procedure

Differences of Least Squares Means											
**Effect**	**Sex**	**Genotype**	**Dose**	**Sex**	**Genotype**	**Dose**	**Estimate**	**Standard Error**	**DF**	**t Value**	**Pr > |t|**
**Genotype*Dose**		RY	6		WT	0	24.5475	50.4466	175	0.49	0.6271
**Genotype*Dose**		RY	0		WT	0.1	-57.7306	50.2477	173	-1.15	0.2522
**Genotype*Dose**		RY	0		WT	1	-48.4862	50.4041	175	-0.96	0.3374
**Genotype*Dose**		RY	0		WT	6	-80.0181	50.2893	173	-1.59	0.1134
**Genotype*Dose**		RY	0		WT	0	-5.7241	50.2477	173	-0.11	0.9094
**Genotype*Dose**		WT	0.1		WT	1	9.2443	50.4041	175	0.18	0.8547
**Genotype*Dose**		WT	0.1		WT	6	-22.2875	50.2893	173	-0.44	0.6582
**Genotype*Dose**		WT	0.1		WT	0	52.0065	50.2477	173	1.04	0.3021
**Genotype*Dose**		WT	1		WT	6	-31.5318	50.4456	175	-0.63	0.5327
**Genotype*Dose**		WT	1		WT	0	42.7621	50.4042	175	0.85	0.3974
**Genotype*Dose**		WT	6		WT	0	74.2940	50.2893	173	1.48	0.1414

Mixed model for cell body area

The Mixed Procedure

Solution for Fixed Effects											
**Effect**	**Sex**	**Genotype**	**Dose**	**Estimate**	**Standard Error**	**DF**	**t Value**	**Pr > |t|**	**Alpha**	**Lower**	**Upper**
**Intercept**				241.25	5.5007	182	43.86	<.0001	0.05	230.40	252.10
**Sex**	F			1.7592	3.8946	183	0.45	0.6520	0.05	-5.9250	9.4433
**Sex**	M			0	.	.	.	.	.	.	.
**Genotype**		CG		1.4624	5.5043	182	0.27	0.7908	0.05	-9.3978	12.3226
**Genotype**		DM		-9.6562	5.5112	183	-1.75	0.0814	0.05	-20.5298	1.2173
**Genotype**		RY		-12.3720	5.5137	183	-2.24	0.0260	0.05	-23.2505	-1.4935
**Genotype**		WT		0	.	.	.	.	.	.	.
**Dose**			0.1	14.7067	5.4950	181	2.68	0.0081	0.05	3.8643	25.5491
**Dose**			1	11.1504	5.4995	182	2.03	0.0441	0.05	0.2993	22.0015
**Dose**			6	8.5039	5.5130	184	1.54	0.1247	0.05	-2.3731	19.3809
**Dose**			0	0	.	.	.	.	.	.	.

Type 3 Tests of Fixed Effects				
**Effect**	**Num DF**	**Den DF**	**F Value**	**Pr > F**
**Sex**	1	183	0.20	0.6520
**Genotype**	3	183	3.14	0.0267
**Dose**	3	183	2.60	0.0536

Least Squares Means					
**Effect**	**Sex**	**Genotype**	**Dose**	**Estimate**	**Standard Error**
**Sex**	F			246.46	2.7539
**Sex**	M			244.70	2.7539
**Genotype**		CG		252.18	3.8856
**Genotype**		DM		241.06	3.8954
**Genotype**		RY		238.35	3.8990
**Genotype**		WT		250.72	3.8986
**Dose**			0.1	251.69	3.8879
**Dose**			1	248.14	3.8942

Mixed model for cell body area

The Mixed Procedure

Least Squares Means					
**Effect**	**Sex**	**Genotype**	**Dose**	**Estimate**	**Standard Error**
**Dose**			6	245.49	3.9132
**Dose**			0	236.99	3.8832

Differences of Least Squares Means											
**Effect**	**Sex**	**Genotype**	**Dose**	**Sex**	**Genotype**	**Dose**	**Estimate**	**Standard Error**	**DF**	**t Value**	**Pr > |t|**
**Sex**	F			M			1.7592	3.8946	183	0.45	0.6520
**Genotype**		CG			DM		11.1186	5.5020	182	2.02	0.0448
**Genotype**		CG			RY		13.8344	5.5045	182	2.51	0.0128
**Genotype**		CG			WT		1.4624	5.5043	182	0.27	0.7908
**Genotype**		DM			RY		2.7158	5.5114	183	0.49	0.6228
**Genotype**		DM			WT		-9.6562	5.5112	183	-1.75	0.0814
**Genotype**		RY			WT		-12.3720	5.5137	183	-2.24	0.0260
**Dose**			0.1			1	3.5563	5.5028	182	0.65	0.5189
**Dose**			0.1			6	6.2028	5.5163	184	1.12	0.2623
**Dose**			0.1			0	14.7067	5.4950	181	2.68	0.0081
**Dose**			1			6	2.6465	5.5208	184	0.48	0.6322
**Dose**			1			0	11.1504	5.4995	182	2.03	0.0441
**Dose**			6			0	8.5039	5.5130	184	1.54	0.1247

Mixed model for tips per dendrite

The Mixed Procedure

Solution for Fixed Effects											
**Effect**	**Sex**	**Genotype**	**Dose**	**Estimate**	**Standard Error**	**DF**	**t Value**	**Pr > |t|**	**Alpha**	**Lower**	**Upper**
**Intercept**				1.9491	0.03974	184	49.05	<.0001	0.05	1.8707	2.0275
**Sex**	F			0.03566	0.02815	185	1.27	0.2069	0.05	-0.01988	0.09121
**Sex**	M			0	.	.	.	.	.	.	.
**Genotype**		CG		0.04820	0.03977	185	1.21	0.2271	0.05	-0.03027	0.1267
**Genotype**		DM		-0.01594	0.03986	186	-0.40	0.6897	0.05	-0.09457	0.06270
**Genotype**		RY		-0.01231	0.03988	186	-0.31	0.7579	0.05	-0.09100	0.06637
**Genotype**		WT		0	.	.	.	.	.	.	.
**Dose**			0.1	-0.04131	0.03967	183	-1.04	0.2990	0.05	-0.1196	0.03695
**Dose**			1	0.03790	0.03971	184	0.95	0.3412	0.05	-0.04046	0.1162
**Dose**			6	-0.05027	0.03988	186	-1.26	0.2091	0.05	-0.1289	0.02841
**Dose**			0	0	.	.	.	.	.	.	.

Type 3 Tests of Fixed Effects				
**Effect**	**Num DF**	**Den DF**	**F Value**	**Pr > F**
**Sex**	1	185	1.60	0.2069
**Genotype**	3	185	1.11	0.3459
**Dose**	3	185	2.08	0.1050

Least Squares Means					
**Effect**	**Sex**	**Genotype**	**Dose**	**Estimate**	**Standard Error**
**Sex**	F			1.9763	0.01991
**Sex**	M			1.9406	0.01991
**Genotype**		CG		2.0017	0.02805
**Genotype**		DM		1.9375	0.02817
**Genotype**		RY		1.9412	0.02820
**Genotype**		WT		1.9535	0.02820
**Dose**			0.1	1.9306	0.02808
**Dose**			1	2.0098	0.02814

Mixed model for tips per dendrite

The Mixed Procedure

Least Squares Means					
**Effect**	**Sex**	**Genotype**	**Dose**	**Estimate**	**Standard Error**
**Dose**			6	1.9216	0.02838
**Dose**			0	1.9719	0.02802

Differences of Least Squares Means											
**Effect**	**Sex**	**Genotype**	**Dose**	**Sex**	**Genotype**	**Dose**	**Estimate**	**Standard Error**	**DF**	**t Value**	**Pr > |t|**
**Sex**	F			M			0.03566	0.02815	185	1.27	0.2069
**Genotype**		CG			DM		0.06414	0.03975	184	1.61	0.1083
**Genotype**		CG			RY		0.06052	0.03978	185	1.52	0.1299
**Genotype**		CG			WT		0.04820	0.03977	185	1.21	0.2271
**Genotype**		DM			RY		-0.00362	0.03986	186	-0.09	0.9276
**Genotype**		DM			WT		-0.01594	0.03986	186	-0.40	0.6897
**Genotype**		RY			WT		-0.01231	0.03988	186	-0.31	0.7579
**Dose**			0.1			1	-0.07921	0.03975	184	-1.99	0.0478
**Dose**			0.1			6	0.008953	0.03992	187	0.22	0.8228
**Dose**			0.1			0	-0.04131	0.03967	183	-1.04	0.2990
**Dose**			1			6	0.08816	0.03997	188	2.21	0.0286
**Dose**			1			0	0.03790	0.03971	184	0.95	0.3412
**Dose**			6			0	-0.05027	0.03988	186	-1.26	0.2091

Mixed model for dendritic mean length

The Mixed Procedure

Solution for Fixed Effects											
**Effect**	**Sex**	**Genotype**	**Dose**	**Estimate**	**Standard Error**	**DF**	**t Value**	**Pr > |t|**	**Alpha**	**Lower**	**Upper**
**Intercept**				104.20	3.5329	182	29.49	<.0001	0.05	97.2269	111.17
**Sex**	F			1.0544	2.5021	183	0.42	0.6740	0.05	-3.8823	5.9911
**Sex**	M			0	.	.	.	.	.	.	.
**Genotype**		CG		4.9347	3.5356	182	1.40	0.1645	0.05	-2.0413	11.9107
**Genotype**		DM		0.3581	3.5413	183	0.10	0.9196	0.05	-6.6288	7.3449
**Genotype**		RY		-0.08195	3.5432	184	-0.02	0.9816	0.05	-7.0725	6.9086
**Genotype**		WT		0	.	.	.	.	.	.	.
**Dose**			0.1	-0.4280	3.5283	181	-0.12	0.9036	0.05	-7.3898	6.5338
**Dose**			1	3.7995	3.5317	182	1.08	0.2834	0.05	-3.1690	10.7679
**Dose**			6	-2.3990	3.5428	184	-0.68	0.4992	0.05	-9.3887	4.5907
**Dose**			0	0	.	.	.	.	.	.	.

Type 3 Tests of Fixed Effects				
**Effect**	**Num DF**	**Den DF**	**F Value**	**Pr > F**
**Sex**	1	183	0.18	0.6740
**Genotype**	3	183	0.95	0.4197
**Dose**	3	183	1.07	0.3639

Least Squares Means					
**Effect**	**Sex**	**Genotype**	**Dose**	**Estimate**	**Standard Error**
**Sex**	F			106.80	1.7693
**Sex**	M			105.74	1.7693
**Genotype**		CG		109.90	2.4949
**Genotype**		DM		105.33	2.5028
**Genotype**		RY		104.89	2.5055
**Genotype**		WT		104.97	2.5053
**Dose**			0.1	105.60	2.4968
**Dose**			1	109.83	2.5016

Mixed model for dendritic mean length

The Mixed Procedure

Least Squares Means					
**Effect**	**Sex**	**Genotype**	**Dose**	**Estimate**	**Standard Error**
**Dose**			6	103.63	2.5172
**Dose**			0	106.03	2.4930

Differences of Least Squares Means											
**Effect**	**Sex**	**Genotype**	**Dose**	**Sex**	**Genotype**	**Dose**	**Estimate**	**Standard Error**	**DF**	**t Value**	**Pr > |t|**
**Sex**	F			M			1.0544	2.5021	183	0.42	0.6740
**Genotype**		CG			DM		4.5766	3.5339	182	1.30	0.1969
**Genotype**		CG			RY		5.0166	3.5358	182	1.42	0.1577
**Genotype**		CG			WT		4.9347	3.5356	182	1.40	0.1645
**Genotype**		DM			RY		0.4400	3.5414	183	0.12	0.9012
**Genotype**		DM			WT		0.3581	3.5413	183	0.10	0.9196
**Genotype**		RY			WT		-0.08195	3.5432	184	-0.02	0.9816
**Dose**			0.1			1	-4.2274	3.5344	182	-1.20	0.2332
**Dose**			0.1			6	1.9710	3.5455	184	0.56	0.5789
**Dose**			0.1			0	-0.4280	3.5283	181	-0.12	0.9036
**Dose**			1			6	6.1985	3.5489	185	1.75	0.0824
**Dose**			1			0	3.7995	3.5317	182	1.08	0.2834
**Dose**			6			0	-2.3990	3.5428	184	-0.68	0.4992

Mixed model for Nodes

The Mixed Procedure

Solution for Fixed Effects											
**Effect**	**Sex**	**Genotype**	**Dose**	**Estimate**	**Standard Error**	**DF**	**t Value**	**Pr > |t|**	**Alpha**	**Lower**	**Upper**
**Intercept**				5.2337	0.2382	183	21.97	<.0001	0.05	4.7638	5.7036
**Sex**	F			0.2403	0.1686	184	1.42	0.1559	0.05	-0.09246	0.5730
**Sex**	M			0	.	.	.	.	.	.	.
**Genotype**		CG		0.2296	0.2383	183	0.96	0.3367	0.05	-0.2406	0.6998
**Genotype**		DM		-0.06773	0.2386	184	-0.28	0.7769	0.05	-0.5386	0.4031
**Genotype**		RY		0.04704	0.2388	184	0.20	0.8440	0.05	-0.4240	0.5181
**Genotype**		WT		0	.	.	.	.	.	.	.
**Dose**			0.1	-0.1305	0.2379	182	-0.55	0.5840	0.05	-0.5999	0.3389
**Dose**			1	0.5842	0.2381	182	2.45	0.0151	0.05	0.1143	1.0540
**Dose**			6	-0.2769	0.2387	184	-1.16	0.2475	0.05	-0.7479	0.1940
**Dose**			0	0	.	.	.	.	.	.	.

Type 3 Tests of Fixed Effects				
**Effect**	**Num DF**	**Den DF**	**F Value**	**Pr > F**
**Sex**	1	184	2.03	0.1559
**Genotype**	3	184	0.57	0.6346
**Dose**	3	184	5.00	0.0024

Least Squares Means					
**Effect**	**Sex**	**Genotype**	**Dose**	**Estimate**	**Standard Error**
**Sex**	F			5.5703	0.1192
**Sex**	M			5.3301	0.1192
**Genotype**		CG		5.6276	0.1682
**Genotype**		DM		5.3303	0.1687
**Genotype**		RY		5.4450	0.1688
**Genotype**		WT		5.3980	0.1688
**Dose**			0.1	5.2755	0.1683
**Dose**			1	5.9902	0.1686

Mixed model for Nodes

The Mixed Procedure

Least Squares Means					
**Effect**	**Sex**	**Genotype**	**Dose**	**Estimate**	**Standard Error**
**Dose**			6	5.1291	0.1695
**Dose**			0	5.4060	0.1681

Differences of Least Squares Means											
**Effect**	**Sex**	**Genotype**	**Dose**	**Sex**	**Genotype**	**Dose**	**Estimate**	**Standard Error**	**DF**	**t Value**	**Pr > |t|**
**Sex**	F			M			0.2403	0.1686	184	1.42	0.1559
**Genotype**		CG			DM		0.2973	0.2382	183	1.25	0.2136
**Genotype**		CG			RY		0.1825	0.2383	183	0.77	0.4447
**Genotype**		CG			WT		0.2296	0.2383	183	0.96	0.3367
**Genotype**		DM			RY		-0.1148	0.2386	184	-0.48	0.6311
**Genotype**		DM			WT		-0.06773	0.2386	184	-0.28	0.7769
**Genotype**		RY			WT		0.04704	0.2388	184	0.20	0.8440
**Dose**			0.1			1	-0.7147	0.2383	183	-3.00	0.0031
**Dose**			0.1			6	0.1464	0.2389	185	0.61	0.5406
**Dose**			0.1			0	-0.1305	0.2379	182	-0.55	0.5840
**Dose**			1			6	0.8611	0.2391	185	3.60	0.0004
**Dose**			1			0	0.5842	0.2381	182	2.45	0.0151
**Dose**			6			0	-0.2769	0.2387	184	-1.16	0.2475
