# Supplementary material for: Sex and Genotype Modulate the Dendritic Effects of Developmental Exposure to a Human-Relevant Polychlorinated Biphenyls Mixture in the Juvenile Mouse
Source: Front Neurosci. 2021 Dec 3;15:766802. doi: 10.3389/fnins.2021.766802 (PMC8678536; doi:10.3389/fnins.2021.766802)
Supplement: Supplementary file 2 [file Data_Sheet_2.PDF]

Hippocampus Sholl Profile Analysis

The Mixed Procedure

Solution for Fixed Effects											
**Effect**	**Sex**	**Genotype**	**Dose**	**Estimate**	**Standard Error**	**DF**	**t Value**	**Pr > |t|**	**Alpha**	**Lower**	**Upper**
**Intercept**				2.9680	0.1263	184	23.50	<.0001	0.05	2.7188	3.2172
**Sex**	F			0.05471	0.08887	35E3	0.62	0.5382	0.05	-0.1195	0.2289
**Sex**	M			0	.	.	.	.	.	.	.
**Genotype**		CG		0.04939	0.1255	35E3	0.39	0.6939	0.05	-0.1966	0.2954
**Genotype**		DM		-0.01685	0.1261	35E3	-0.13	0.8937	0.05	-0.2641	0.2304
**Genotype**		RY		-0.1633	0.1255	35E3	-1.30	0.1932	0.05	-0.4094	0.08271
**Genotype**		WT		0	.	.	.	.	.	.	.
**Dose**			0.1	0.1027	0.1260	35E3	0.82	0.4149	0.05	-0.1443	0.3497
**Dose**			1	0.1956	0.1257	35E3	1.56	0.1195	0.05	-0.05065	0.4419
**Dose**			6	0.1160	0.1257	35E3	0.92	0.3561	0.05	-0.1303	0.3623
**Dose**			0	0	.	.	.	.	.	.	.

Type 3 Tests of Fixed Effects				
**Effect**	**Num DF**	**Den DF**	**F Value**	**Pr > F**
**Sex**	1	35E3	0.38	0.5382
**Genotype**	3	35E3	1.07	0.3616
**Dose**	3	35E3	0.82	0.4843

Least Squares Means					
**Effect**	**Sex**	**Genotype**	**Dose**	**Estimate**	**Standard Error**
**Sex**	F			3.0936	0.06275
**Sex**	M			3.0389	0.06293
**Genotype**		CG		3.1483	0.08854
**Genotype**		DM		3.0821	0.08941
**Genotype**		RY		2.9356	0.08858
**Genotype**		WT		3.0989	0.08896
**Dose**			0.1	3.0654	0.08915
**Dose**			1	3.1583	0.08864

Hippocampus Sholl Profile Analysis

The Mixed Procedure

Least Squares Means					
**Effect**	**Sex**	**Genotype**	**Dose**	**Estimate**	**Standard Error**
**Dose**			6	3.0786	0.08863
**Dose**			0	2.9627	0.08907

Differences of Least Squares Means											
**Effect**	**Sex**	**Genotype**	**Dose**	**Sex**	**Genotype**	**Dose**	**Estimate**	**Standard Error**	**DF**	**t Value**	**Pr > |t|**
**Sex**	F			M			0.05471	0.08887	35E3	0.62	0.5382
**Genotype**		CG			DM		0.06625	0.1258	35E3	0.53	0.5986
**Genotype**		CG			RY		0.2127	0.1252	35E3	1.70	0.0894
**Genotype**		CG			WT		0.04939	0.1255	35E3	0.39	0.6939
**Genotype**		DM			RY		0.1465	0.1259	35E3	1.16	0.2445
**Genotype**		DM			WT		-0.01685	0.1261	35E3	-0.13	0.8937
**Genotype**		RY			WT		-0.1633	0.1255	35E3	-1.30	0.1932
**Dose**			0.1			1	-0.09291	0.1257	35E3	-0.74	0.4599
**Dose**			0.1			6	-0.01323	0.1257	35E3	-0.11	0.9162
**Dose**			0.1			0	0.1027	0.1260	35E3	0.82	0.4149
**Dose**			1			6	0.07968	0.1253	35E3	0.64	0.5250
**Dose**			1			0	0.1956	0.1257	35E3	1.56	0.1195
**Dose**			6			0	0.1160	0.1257	35E3	0.92	0.3561

Mixed model for Peak X

The Mixed Procedure

Solution for Fixed Effects											
Effect	Sex	Genotype	Dose	Estimate	Standard Error	DF	t Value	Pr > |t|	Alpha	Lower	Upper
Intercept				43.7918	1.3964	188	31.36	<.0001	0.05	41.0372	46.5465
Sex	F			0.6046	0.9751	180	0.62	0.5360	0.05	-1.3194	2.5287
Sex	M			0	.	.	.	.	.	.	.
Genotype		CG		3.0639	1.3760	180	2.23	0.0272	0.05	0.3487	5.7791
Genotype		DM		1.9766	1.3886	184	1.42	0.1563	0.05	-0.7630	4.7162
Genotype		RY		0.2493	1.3816	182	0.18	0.8570	0.05	-2.4767	2.9753
Genotype		WT		0	.	.	.	.	.	.	.
Dose			0.1	0.7667	1.3906	185	0.55	0.5821	0.05	-1.9767	3.5102
Dose			1	0.8798	1.3790	181	0.64	0.5243	0.05	-1.8413	3.6008
Dose			6	0.06706	1.3785	180	0.05	0.9613	0.05	-2.6530	2.7871
Dose			0	0	.	.	.	.	.	.	.

Type 3 Tests of Fixed Effects				
Effect	Num DF	Den DF	F Value	Pr > F
Sex	1	180	0.38	0.5360
Genotype	3	181	2.25	0.0846
Dose	3	181	0.22	0.8811

Least Squares Means					
Effect	Sex	Genotype	Dose	Estimate	Standard Error
Sex	F			46.1473	0.6860
Sex	M			45.5427	0.6932
Genotype		CG		47.5865	0.9644
Genotype		DM		46.4992	0.9824
Genotype		RY		44.7718	0.9724
Genotype		WT		44.5225	0.9815
Dose			0.1	46.1833	0.9839
Dose			1	46.2964	0.9673

Mixed model for Peak X

The Mixed Procedure

Least Squares Means					
**Effect**	**Sex**	**Genotype**	**Dose**	**Estimate**	**Standard Error**
**Dose**			6	45.4837	0.9667
**Dose**			0	45.4166	0.9829

Differences of Least Squares Means											
**Effect**	**Sex**	**Genotype**	**Dose**	**Sex**	**Genotype**	**Dose**	**Estimate**	**Standard Error**	**DF**	**t Value**	**Pr > |t|**
**Sex**	F			M			0.6046	0.9751	180	0.62	0.5360
**Genotype**		CG			DM		1.0873	1.3766	179	0.79	0.4307
**Genotype**		CG			RY		2.8146	1.3696	177	2.06	0.0413
**Genotype**		CG			WT		3.0639	1.3760	180	2.23	0.0272
**Genotype**		DM			RY		1.7273	1.3821	181	1.25	0.2130
**Genotype**		DM			WT		1.9766	1.3886	184	1.42	0.1563
**Genotype**		RY			WT		0.2493	1.3816	182	0.18	0.8570
**Dose**			0.1			1	-0.1130	1.3797	181	-0.08	0.9348
**Dose**			0.1			6	0.6997	1.3793	180	0.51	0.6126
**Dose**			0.1			0	0.7667	1.3906	185	0.55	0.5821
**Dose**			1			6	0.8127	1.3676	176	0.59	0.5531
**Dose**			1			0	0.8798	1.3790	181	0.64	0.5243
**Dose**			6			0	0.06706	1.3785	180	0.05	0.9613

Mixed model for Peak Y

The Mixed Procedure

Solution for Fixed Effects											
Effect	Sex	Genotype	Dose	Estimate	Standard Error	DF	t Value	Pr > |t|	Alpha	Lower	Upper
Intercept				11.4162	0.3090	191	36.95	<.0001	0.05	10.8067	12.0256
Sex	F			-0.01392	0.2164	184	-0.06	0.9488	0.05	-0.4409	0.4130
Sex	M			0	.	.	.	.	.	.	.
Genotype		CG		-0.3589	0.3055	184	-1.17	0.2416	0.05	-0.9615	0.2438
Genotype		DM		-0.7233	0.3077	188	-2.35	0.0198	0.05	-1.3303	-0.1162
Genotype		RY		-0.8160	0.3064	186	-2.66	0.0084	0.05	-1.4205	-0.2115
Genotype		WT		0	.	.	.	.	.	.	.
Dose			0.1	-0.04769	0.3081	188	-0.15	0.8771	0.05	-0.6554	0.5600
Dose			1	0.4368	0.3060	185	1.43	0.1551	0.05	-0.1669	1.0405
Dose			6	0.3474	0.3059	184	1.14	0.2576	0.05	-0.2562	0.9510
Dose			0	0	.	.	.	.	.	.	.

Type 3 Tests of Fixed Effects				
Effect	Num DF	Den DF	F Value	Pr > F
Sex	1	184	0.00	0.9488
Genotype	3	184	2.95	0.0339
Dose	3	184	1.27	0.2868

Least Squares Means					
Effect	Sex	Genotype	Dose	Estimate	Standard Error
Sex	F			11.1118	0.1524
Sex	M			11.1258	0.1537
Genotype		CG		11.2345	0.2145
Genotype		DM		10.8701	0.2178
Genotype		RY		10.7773	0.2159
Genotype		WT		11.5933	0.2175
Dose			0.1	10.8870	0.2180
Dose			1	11.3715	0.2150

Mixed model for Peak Y

The Mixed Procedure

Least Squares Means					
Effect	Sex	Genotype	Dose	Estimate	Standard Error
Dose			6	11.2821	0.2150
Dose			0	10.9347	0.2177

Differences of Least Squares Means											
Effect	Sex	Genotype	Dose	Sex	Genotype	Dose	Estimate	Standard Error	DF	t Value	Pr > |t|
Sex	F			M			-0.01392	0.2164	184	-0.06	0.9488
Genotype		CG			DM		0.3644	0.3057	183	1.19	0.2348
Genotype		CG			RY		0.4571	0.3044	181	1.50	0.1349
Genotype		CG			WT		-0.3589	0.3055	184	-1.17	0.2416
Genotype		DM			RY		0.09274	0.3066	185	0.30	0.7627
Genotype		DM			WT		-0.7233	0.3077	188	-2.35	0.0198
Genotype		RY			WT		-0.8160	0.3064	186	-2.66	0.0084
Dose			0.1			1	-0.4845	0.3062	185	-1.58	0.1152
Dose			0.1			6	-0.3951	0.3061	184	-1.29	0.1984
Dose			0.1			0	-0.04769	0.3081	188	-0.15	0.8771
Dose			1			6	0.08943	0.3040	181	0.29	0.7690
Dose			1			0	0.4368	0.3060	185	1.43	0.1551
Dose			6			0	0.3474	0.3059	184	1.14	0.2576

Mixed model for AUC total

The Mixed Procedure

Solution for Fixed Effects											
**Effect**	**Sex**	**Genotype**	**Dose**	**Estimate**	**Standard Error**	**DF**	**t Value**	**Pr > |t|**	**Alpha**	**Lower**	**Upper**
**Intercept**				746.71	31.4671	188	23.73	<.0001	0.05	684.64	808.79
**Sex**	F			5.9542	22.1011	183	0.27	0.7879	0.05	-37.6508	49.5592
**Sex**	M			0	.	.	.	.	.	.	.
**Genotype**		CG		14.0228	31.2061	183	0.45	0.6537	0.05	-47.5477	75.5932
**Genotype**		DM		-11.0598	31.3835	186	-0.35	0.7249	0.05	-72.9737	50.8540
**Genotype**		RY		-37.8532	31.2792	184	-1.21	0.2278	0.05	-99.5647	23.8584
**Genotype**		WT		0	.	.	.	.	.	.	.
**Dose**			0.1	23.1477	31.4071	186	0.74	0.4620	0.05	-38.8115	85.1068
**Dose**			1	48.8843	31.2477	184	1.56	0.1194	0.05	-12.7665	110.54
**Dose**			6	34.4834	31.2459	183	1.10	0.2712	0.05	-27.1644	96.1313
**Dose**			0	0	.	.	.	.	.	.	.

Type 3 Tests of Fixed Effects				
**Effect**	**Num DF**	**Den DF**	**F Value**	**Pr > F**
**Sex**	1	183	0.07	0.7879
**Genotype**	3	183	0.99	0.3969
**Dose**	3	183	0.87	0.4568

Least Squares Means					
**Effect**	**Sex**	**Genotype**	**Dose**	**Estimate**	**Standard Error**
**Sex**	F			770.57	15.5760
**Sex**	M			764.62	15.6804
**Genotype**		CG		790.34	21.9587
**Genotype**		DM		765.26	22.2112
**Genotype**		RY		738.46	22.0630
**Genotype**		WT		776.32	22.1730
**Dose**			0.1	764.11	22.2222
**Dose**			1	789.85	21.9950

Mixed model for AUC total

The Mixed Procedure

Least Squares Means					
**Effect**	**Sex**	**Genotype**	**Dose**	**Estimate**	**Standard Error**
**Dose**			6	775.45	21.9933
**Dose**			0	740.97	22.1955

Differences of Least Squares Means											
**Effect**	**Sex**	**Genotype**	**Dose**	**Sex**	**Genotype**	**Dose**	**Estimate**	**Standard Error**	**DF**	**t Value**	**Pr > |t|**
**Sex**	F			M			5.9542	22.1011	183	0.27	0.7879
**Genotype**		CG			DM		25.0826	31.2333	183	0.80	0.4230
**Genotype**		CG			RY		51.8760	31.1285	181	1.67	0.0973
**Genotype**		CG			WT		14.0228	31.2061	183	0.45	0.6537
**Genotype**		DM			RY		26.7933	31.3059	184	0.86	0.3932
**Genotype**		DM			WT		-11.0598	31.3835	186	-0.35	0.7249
**Genotype**		RY			WT		-37.8532	31.2792	184	-1.21	0.2278
**Dose**			0.1			1	-25.7367	31.2665	183	-0.82	0.4115
**Dose**			0.1			6	-11.3357	31.2650	183	-0.36	0.7173
**Dose**			0.1			0	23.1477	31.4071	186	0.74	0.4620
**Dose**			1			6	14.4009	31.1048	181	0.46	0.6439
**Dose**			1			0	48.8843	31.2477	184	1.56	0.1194
**Dose**			6			0	34.4834	31.2459	183	1.10	0.2712

Mixed model for AUC proximal

The Mixed Procedure

Solution for Fixed Effects											
**Effect**	**Sex**	**Genotype**	**Dose**	**Estimate**	**Standard Error**	**DF**	**t Value**	**Pr > |t|**	**Alpha**	**Lower**	**Upper**
**Intercept**				484.22	14.2223	189	34.05	<.0001	0.05	456.17	512.28
**Sex**	F			-1.6078	9.9701	184	-0.16	0.8721	0.05	-21.2786	18.0629
**Sex**	M			0	.	.	.	.	.	.	.
**Genotype**		CG		-13.6199	14.0742	183	-0.97	0.3345	0.05	-41.3887	14.1488
**Genotype**		DM		-24.2883	14.1711	186	-1.71	0.0882	0.05	-52.2446	3.6680
**Genotype**		RY		-28.3528	14.1153	185	-2.01	0.0460	0.05	-56.2009	-0.5047
**Genotype**		WT		0	.	.	.	.	.	.	.
**Dose**			0.1	1.7967	14.1848	187	0.13	0.8993	0.05	-26.1859	29.7794
**Dose**			1	21.9820	14.0972	184	1.56	0.1206	0.05	-5.8312	49.7952
**Dose**			6	14.3171	14.0953	183	1.02	0.3111	0.05	-13.4925	42.1267
**Dose**			0	0	.	.	.	.	.	.	.

Type 3 Tests of Fixed Effects				
**Effect**	**Num DF**	**Den DF**	**F Value**	**Pr > F**
**Sex**	1	184	0.03	0.8721
**Genotype**	3	184	1.61	0.1892
**Dose**	3	184	1.10	0.3493

Least Squares Means					
**Effect**	**Sex**	**Genotype**	**Dose**	**Estimate**	**Standard Error**
**Sex**	F			475.58	7.0219
**Sex**	M			477.18	7.0785
**Genotype**		CG		479.32	9.8906
**Genotype**		DM		468.66	10.0286
**Genotype**		RY		464.59	9.9493
**Genotype**		WT		492.94	10.0132
**Dose**			0.1	468.65	10.0364
**Dose**			1	488.84	9.9114

Mixed model for AUC proximal

The Mixed Procedure

Least Squares Means					
**Effect**	**Sex**	**Genotype**	**Dose**	**Estimate**	**Standard Error**
**Dose**			6	481.17	9.9091
**Dose**			0	466.86	10.0248

Differences of Least Squares Means											
**Effect**	**Sex**	**Genotype**	**Dose**	**Sex**	**Genotype**	**Dose**	**Estimate**	**Standard Error**	**DF**	**t Value**	**Pr > |t|**
**Sex**	F			M			-1.6078	9.9701	184	-0.16	0.8721
**Genotype**		CG			DM		10.6684	14.0853	183	0.76	0.4498
**Genotype**		CG			RY		14.7329	14.0292	181	1.05	0.2950
**Genotype**		CG			WT		-13.6199	14.0742	183	-0.97	0.3345
**Genotype**		DM			RY		4.0645	14.1260	184	0.29	0.7739
**Genotype**		DM			WT		-24.2883	14.1711	186	-1.71	0.0882
**Genotype**		RY			WT		-28.3528	14.1153	185	-2.01	0.0460
**Dose**			0.1			1	-20.1852	14.1054	184	-1.43	0.1541
**Dose**			0.1			6	-12.5204	14.1036	183	-0.89	0.3758
**Dose**			0.1			0	1.7967	14.1848	187	0.13	0.8993
**Dose**			1			6	7.6648	14.0155	180	0.55	0.5851
**Dose**			1			0	21.9820	14.0972	184	1.56	0.1206
**Dose**			6			0	14.3171	14.0953	183	1.02	0.3111

Mixed model for AUC distal

The Mixed Procedure

Solution for Fixed Effects											
**Effect**	**Sex**	**Genotype**	**Dose**	**Estimate**	**Standard Error**	**DF**	**t Value**	**Pr > |t|**	**Alpha**	**Lower**	**Upper**
**Intercept**				2.1624	0.05113	185	42.29	<.0001	0.05	2.0616	2.2633
**Sex**	F			-0.05370	0.06132	181	-0.88	0.3823	0.05	-0.1747	0.06729
**Sex**	M			0	.	.	.	.	.	.	.
**Genotype**		CG		-0.02750	0.06107	179	-0.45	0.6531	0.05	-0.1480	0.09302
**Genotype**		DM		0.06986	0.06171	184	1.13	0.2591	0.05	-0.05189	0.1916
**Genotype**		RY		-0.06110	0.06131	181	-1.00	0.3203	0.05	-0.1821	0.05987
**Genotype**		WT		0	.	.	.	.	.	.	.
**Dose**			0.1	0.03937	0.04343	182	0.91	0.3659	0.05	-0.04633	0.1251
**Dose**			1	0.03409	0.04320	179	0.79	0.4311	0.05	-0.05116	0.1193
**Dose**			6	0.01600	0.04320	179	0.37	0.7116	0.05	-0.06925	0.1012
**Dose**			0	0	.	.	.	.	.	.	.
**Sex*Genotype**	F	CG		0.1792	0.08628	178	2.08	0.0392	0.05	0.008936	0.3495
**Sex*Genotype**	F	DM		-0.04497	0.08680	181	-0.52	0.6050	0.05	-0.2162	0.1263
**Sex*Genotype**	F	RY		0.1370	0.08649	180	1.58	0.1149	0.05	-0.03364	0.3077
**Sex*Genotype**	F	WT		0	.	.	.	.	.	.	.
**Sex*Genotype**	M	CG		0	.	.	.	.	.	.	.
**Sex*Genotype**	M	DM		0	.	.	.	.	.	.	.
**Sex*Genotype**	M	RY		0	.	.	.	.	.	.	.
**Sex*Genotype**	M	WT		0	.	.	.	.	.	.	.

Type 3 Tests of Fixed Effects				
**Effect**	**Num DF**	**Den DF**	**F Value**	**Pr > F**
**Sex**	1	179	0.21	0.6448
**Genotype**	3	179	0.98	0.4030
**Dose**	3	179	0.34	0.7941
**Sex*Genotype**	3	179	3.08	0.0287

Mixed model for AUC distal

The Mixed Procedure

Least Squares Means				
**Effect**	**Sex**	**Genotype**	**Estimate**	**Standard Error**
**Sex**	F		2.1942	0.02153
**Sex**	M		2.1801	0.02168
**Genotype**		CG	2.2200	0.03035
**Genotype**		DM	2.2053	0.03072
**Genotype**		RY	2.1654	0.03050
**Genotype**		WT	2.1579	0.03066
**Sex*Genotype**	F	CG	2.2828	0.04291
**Sex*Genotype**	F	DM	2.1560	0.04305
**Sex*Genotype**	F	RY	2.2070	0.04301
**Sex*Genotype**	F	WT	2.1311	0.04327
**Sex*Genotype**	M	CG	2.1573	0.04292
**Sex*Genotype**	M	DM	2.2547	0.04383
**Sex*Genotype**	M	RY	2.1237	0.04326
**Sex*Genotype**	M	WT	2.1848	0.04345

Differences of Least Squares Means									
**Effect**	**Sex**	**Genotype**	**Sex**	**Genotype**	**Estimate**	**Standard Error**	**DF**	**t Value**	**Pr > |t|**
**Sex**	F		M		0.01411	0.03056	179	0.46	0.6448
**Genotype**		CG		DM	0.01472	0.04318	178	0.34	0.7336
**Genotype**		CG		RY	0.05469	0.04302	176	1.27	0.2053
**Genotype**		CG		WT	0.06210	0.04314	178	1.44	0.1517
**Genotype**		DM		RY	0.03997	0.04329	180	0.92	0.3570
**Genotype**		DM		WT	0.04738	0.04340	181	1.09	0.2764
**Genotype**		RY		WT	0.007410	0.04325	180	0.17	0.8641
**Sex*Genotype**	F	CG	F	DM	0.1268	0.06079	176	2.09	0.0384
**Sex*Genotype**	F	CG	F	RY	0.07577	0.06075	176	1.25	0.2140
**Sex*Genotype**	F	CG	F	WT	0.1517	0.06094	178	2.49	0.0137
**Sex*Genotype**	F	CG	M	CG	0.1255	0.06069	175	2.07	0.0401
**Sex*Genotype**	F	CG	M	DM	0.02813	0.06134	180	0.46	0.6470
**Sex*Genotype**	F	CG	M	RY	0.1591	0.06093	177	2.61	0.0098
**Sex*Genotype**	F	CG	M	WT	0.09800	0.06107	179	1.60	0.1103

Mixed model for AUC distal

The Mixed Procedure

Differences of Least Squares Means									
**Effect**	**Sex**	**Genotype**	**Sex**	**Genotype**	**Estimate**	**Standard Error**	**DF**	**t Value**	**Pr > |t|**
**Sex*Genotype**	F	DM	F	RY	-0.05103	0.06085	177	-0.84	0.4028
**Sex*Genotype**	F	DM	F	WT	0.02490	0.06104	179	0.41	0.6839
**Sex*Genotype**	F	DM	M	CG	-0.00131	0.06079	176	-0.02	0.9828
**Sex*Genotype**	F	DM	M	DM	-0.09867	0.06144	181	-1.61	0.1100
**Sex*Genotype**	F	DM	M	RY	0.03230	0.06103	178	0.53	0.5973
**Sex*Genotype**	F	DM	M	WT	-0.02881	0.06117	180	-0.47	0.6382
**Sex*Genotype**	F	RY	F	WT	0.07593	0.06101	178	1.24	0.2149
**Sex*Genotype**	F	RY	M	CG	0.04972	0.06076	176	0.82	0.4143
**Sex*Genotype**	F	RY	M	DM	-0.04764	0.06140	181	-0.78	0.4388
**Sex*Genotype**	F	RY	M	RY	0.08333	0.06100	178	1.37	0.1736
**Sex*Genotype**	F	RY	M	WT	0.02222	0.06113	179	0.36	0.7166
**Sex*Genotype**	F	WT	M	CG	-0.02621	0.06095	178	-0.43	0.6677
**Sex*Genotype**	F	WT	M	DM	-0.1236	0.06159	183	-2.01	0.0463
**Sex*Genotype**	F	WT	M	RY	0.007403	0.06119	180	0.12	0.9038
**Sex*Genotype**	F	WT	M	WT	-0.05370	0.06132	181	-0.88	0.3823
**Sex*Genotype**	M	CG	M	DM	-0.09736	0.06134	180	-1.59	0.1142
**Sex*Genotype**	M	CG	M	RY	0.03361	0.06094	177	0.55	0.5820
**Sex*Genotype**	M	CG	M	WT	-0.02750	0.06107	179	-0.45	0.6531
**Sex*Genotype**	M	DM	M	RY	0.1310	0.06158	182	2.13	0.0348
**Sex*Genotype**	M	DM	M	WT	0.06986	0.06171	184	1.13	0.2591
**Sex*Genotype**	M	RY	M	WT	-0.06110	0.06131	181	-1.00	0.3203

Mixed Effects Model for Total Dendrites

The Mixed Procedure

Solution for Fixed Effects											
**Effect**	**Sex**	**Genotype**	**Dose**	**Estimate**	**Standard Error**	**DF**	**t Value**	**Pr > |t|**	**Alpha**	**Lower**	**Upper**
**Intercept**				4.2803	0.1167	191	36.68	<.0001	0.05	4.0501	4.5105
**Sex**	F			-0.03366	0.08143	183	-0.41	0.6798	0.05	-0.1943	0.1270
**Sex**	M			0	.	.	.	.	.	.	.
**Genotype**		CG		-0.2756	0.1149	182	-2.40	0.0175	0.05	-0.5023	-0.04888
**Genotype**		DM		-0.3929	0.1160	187	-3.39	0.0009	0.05	-0.6217	-0.1640
**Genotype**		RY		-0.3514	0.1154	185	-3.04	0.0027	0.05	-0.5790	-0.1237
**Genotype**		WT		0	.	.	.	.	.	.	.
**Dose**			0.1	-0.09712	0.1162	188	-0.84	0.4043	0.05	-0.3263	0.1321
**Dose**			1	-0.01217	0.1152	183	-0.11	0.9160	0.05	-0.2394	0.2151
**Dose**			6	0.09735	0.1151	183	0.85	0.3988	0.05	-0.1298	0.3245
**Dose**			0	0	.	.	.	.	.	.	.

Type 3 Tests of Fixed Effects				
**Effect**	**Num DF**	**Den DF**	**F Value**	**Pr > F**
**Sex**	1	183	0.17	0.6798
**Genotype**	3	183	4.66	0.0037
**Dose**	3	183	0.96	0.4142

Least Squares Means					
**Effect**	**Sex**	**Genotype**	**Dose**	**Estimate**	**Standard Error**
**Sex**	F			3.9887	0.05727
**Sex**	M			4.0223	0.05790
**Genotype**		CG		3.9849	0.08049
**Genotype**		DM		3.8676	0.08206
**Genotype**		RY		3.9091	0.08120
**Genotype**		WT		4.2605	0.08201
**Dose**			0.1	3.9114	0.08221
**Dose**			1	3.9963	0.08075

Mixed Effects Model for Total Dendrites

The Mixed Procedure

Least Squares Means					
**Effect**	**Sex**	**Genotype**	**Dose**	**Estimate**	**Standard Error**
**Dose**			6	4.1058	0.08069
**Dose**			0	4.0085	0.08212

Differences of Least Squares Means											
**Effect**	**Sex**	**Genotype**	**Dose**	**Sex**	**Genotype**	**Dose**	**Estimate**	**Standard Error**	**DF**	**t Value**	**Pr > |t|**
**Sex**	F			M			-0.03366	0.08143	183	-0.41	0.6798
**Genotype**		CG			DM		0.1173	0.1150	182	1.02	0.3089
**Genotype**		CG			RY		0.07576	0.1143	179	0.66	0.5084
**Genotype**		CG			WT		-0.2756	0.1149	182	-2.40	0.0175
**Genotype**		DM			RY		-0.04153	0.1154	184	-0.36	0.7194
**Genotype**		DM			WT		-0.3929	0.1160	187	-3.39	0.0009
**Genotype**		RY			WT		-0.3514	0.1154	185	-3.04	0.0027
**Dose**			0.1			1	-0.08496	0.1152	183	-0.74	0.4619
**Dose**			0.1			6	-0.1945	0.1152	183	-1.69	0.0930
**Dose**			0.1			0	-0.09712	0.1162	188	-0.84	0.4043
**Dose**			1			6	-0.1095	0.1142	178	-0.96	0.3387
**Dose**			1			0	-0.01217	0.1152	183	-0.11	0.9160
**Dose**			6			0	0.09735	0.1151	183	0.85	0.3988

Mixed Effects Model for terminal tips

The Mixed Procedure

Solution for Fixed Effects											
**Effect**	**Sex**	**Genotype**	**Dose**	**Estimate**	**Standard Error**	**DF**	**t Value**	**Pr > |t|**	**Alpha**	**Lower**	**Upper**
**Intercept**				15.4589	0.4666	190	33.13	<.0001	0.05	14.5386	16.3792
**Sex**	F			-0.08898	0.3271	184	-0.27	0.7859	0.05	-0.7343	0.5564
**Sex**	M			0	.	.	.	.	.	.	.
**Genotype**		CG		-0.08570	0.4617	184	-0.19	0.8530	0.05	-0.9967	0.8253
**Genotype**		DM		-0.8187	0.4649	187	-1.76	0.0799	0.05	-1.7358	0.09845
**Genotype**		RY		-0.9146	0.4631	185	-1.98	0.0497	0.05	-1.8282	-0.00103
**Genotype**		WT		0	.	.	.	.	.	.	.
**Dose**			0.1	0.1680	0.4653	188	0.36	0.7185	0.05	-0.7500	1.0860
**Dose**			1	0.5770	0.4625	185	1.25	0.2138	0.05	-0.3355	1.4895
**Dose**			6	0.3821	0.4624	184	0.83	0.4097	0.05	-0.5303	1.2944
**Dose**			0	0	.	.	.	.	.	.	.

Type 3 Tests of Fixed Effects				
**Effect**	**Num DF**	**Den DF**	**F Value**	**Pr > F**
**Sex**	1	184	0.07	0.7859
**Genotype**	3	184	2.14	0.0968
**Dose**	3	184	0.59	0.6217

Least Squares Means					
**Effect**	**Sex**	**Genotype**	**Dose**	**Estimate**	**Standard Error**
**Sex**	F			15.1969	0.2304
**Sex**	M			15.2859	0.2322
**Genotype**		CG		15.6105	0.3245
**Genotype**		DM		14.8775	0.3290
**Genotype**		RY		14.7816	0.3264
**Genotype**		WT		15.6962	0.3285
**Dose**			0.1	15.1277	0.3293
**Dose**			1	15.5367	0.3252

Mixed Effects Model for terminal tips

The Mixed Procedure

Least Squares Means					
**Effect**	**Sex**	**Genotype**	**Dose**	**Estimate**	**Standard Error**
**Dose**			6	15.3417	0.3251
**Dose**			0	14.9597	0.3289

Differences of Least Squares Means											
**Effect**	**Sex**	**Genotype**	**Dose**	**Sex**	**Genotype**	**Dose**	**Estimate**	**Standard Error**	**DF**	**t Value**	**Pr > |t|**
**Sex**	F			M			-0.08898	0.3271	184	-0.27	0.7859
**Genotype**		CG			DM		0.7330	0.4621	183	1.59	0.1144
**Genotype**		CG			RY		0.8289	0.4603	182	1.80	0.0734
**Genotype**		CG			WT		-0.08570	0.4617	184	-0.19	0.8530
**Genotype**		DM			RY		0.09595	0.4634	185	0.21	0.8362
**Genotype**		DM			WT		-0.8187	0.4649	187	-1.76	0.0799
**Genotype**		RY			WT		-0.9146	0.4631	185	-1.98	0.0497
**Dose**			0.1			1	-0.4090	0.4628	184	-0.88	0.3780
**Dose**			0.1			6	-0.2141	0.4627	184	-0.46	0.6442
**Dose**			0.1			0	0.1680	0.4653	188	0.36	0.7185
**Dose**			1			6	0.1949	0.4598	181	0.42	0.6722
**Dose**			1			0	0.5770	0.4625	185	1.25	0.2138
**Dose**			6			0	0.3821	0.4624	184	0.83	0.4097

Mixed Effects Model for dendrite length sum

The Mixed Procedure

Solution for Fixed Effects											
**Effect**	**Sex**	**Genotype**	**Dose**	**Estimate**	**Standard Error**	**DF**	**t Value**	**Pr > |t|**	**Alpha**	**Lower**	**Upper**
**Intercept**				857.44	35.6230	188	24.07	<.0001	0.05	787.16	927.71
**Sex**	F			11.2111	25.0221	184	0.45	0.6546	0.05	-38.1567	60.5789
**Sex**	M			0	.	.	.	.	.	.	.
**Genotype**		CG		9.0551	35.3309	183	0.26	0.7980	0.05	-60.6532	78.7634
**Genotype**		DM		-17.7112	35.5299	186	-0.50	0.6187	0.05	-87.8046	52.3821
**Genotype**		RY		-52.4407	35.4128	184	-1.48	0.1404	0.05	-122.31	17.4255
**Genotype**		WT		0	.	.	.	.	.	.	.
**Dose**			0.1	33.6612	35.5562	187	0.95	0.3450	0.05	-36.4828	103.81
**Dose**			1	53.3977	35.3775	184	1.51	0.1329	0.05	-16.4007	123.20
**Dose**			6	36.5092	35.3757	184	1.03	0.3034	0.05	-33.2860	106.30
**Dose**			0	0	.	.	.	.	.	.	.

Type 3 Tests of Fixed Effects				
**Effect**	**Num DF**	**Den DF**	**F Value**	**Pr > F**
**Sex**	1	184	0.20	0.6546
**Genotype**	3	184	1.18	0.3173
**Dose**	3	184	0.80	0.4970

Least Squares Means					
**Effect**	**Sex**	**Genotype**	**Dose**	**Estimate**	**Standard Error**
**Sex**	F			884.26	17.6352
**Sex**	M			873.05	17.7523
**Genotype**		CG		902.99	24.8627
**Genotype**		DM		876.22	25.1457
**Genotype**		RY		841.49	24.9794
**Genotype**		WT		893.93	25.1024
**Dose**			0.1	881.43	25.1579
**Dose**			1	901.16	24.9033

Mixed Effects Model for dendrite length sum

The Mixed Procedure

Least Squares Means					
**Effect**	**Sex**	**Genotype**	**Dose**	**Estimate**	**Standard Error**
**Dose**			6	884.28	24.9015
**Dose**			0	847.77	25.1276

Differences of Least Squares Means											
**Effect**	**Sex**	**Genotype**	**Dose**	**Sex**	**Genotype**	**Dose**	**Estimate**	**Standard Error**	**DF**	**t Value**	**Pr > |t|**
**Sex**	F			M			11.2111	25.0221	184	0.45	0.6546
**Genotype**		CG			DM		26.7664	35.3618	183	0.76	0.4501
**Genotype**		CG			RY		61.4958	35.2442	181	1.74	0.0827
**Genotype**		CG			WT		9.0551	35.3309	183	0.26	0.7980
**Genotype**		DM			RY		34.7295	35.4431	184	0.98	0.3284
**Genotype**		DM			WT		-17.7112	35.5299	186	-0.50	0.6187
**Genotype**		RY			WT		-52.4407	35.4128	184	-1.48	0.1404
**Dose**			0.1			1	-19.7365	35.3989	184	-0.56	0.5778
**Dose**			0.1			6	-2.8481	35.3972	184	-0.08	0.9360
**Dose**			0.1			0	33.6612	35.5562	187	0.95	0.3450
**Dose**			1			6	16.8885	35.2177	181	0.48	0.6321
**Dose**			1			0	53.3977	35.3775	184	1.51	0.1329
**Dose**			6			0	36.5092	35.3757	184	1.03	0.3034

Mixed model for cell body area

The Mixed Procedure

Solution for Fixed Effects											
**Effect**	**Sex**	**Genotype**	**Dose**	**Estimate**	**Standard Error**	**DF**	**t Value**	**Pr > |t|**	**Alpha**	**Lower**	**Upper**
**Intercept**				291.17	11.2507	187	25.88	<.0001	0.05	268.97	313.36
**Sex**	F			9.2350	5.3625	175	1.72	0.0868	0.05	-1.3482	19.8182
**Sex**	M			0	.	.	.	.	.	.	.
**Genotype**		CG		-26.4546	15.2232	178	-1.74	0.0840	0.05	-56.4956	3.5864
**Genotype**		DM		-61.3191	15.3539	183	-3.99	<.0001	0.05	-91.6121	-31.0260
**Genotype**		RY		-53.0510	15.3287	183	-3.46	0.0007	0.05	-83.2951	-22.8068
**Genotype**		WT		0	.	.	.	.	.	.	.
**Dose**			0.1	-22.6638	15.3565	184	-1.48	0.1417	0.05	-52.9610	7.6334
**Dose**			1	-19.0021	15.2795	180	-1.24	0.2152	0.05	-49.1515	11.1474
**Dose**			6	-55.1461	15.2381	179	-3.62	0.0004	0.05	-85.2158	-25.0763
**Dose**			0	0	.	.	.	.	.	.	.
**Genotype*Dose**		CG	0.1	9.0472	21.5218	178	0.42	0.6747	0.05	-33.4232	51.5177
**Genotype*Dose**		CG	1	7.3856	21.4762	177	0.34	0.7313	0.05	-34.9972	49.7684
**Genotype*Dose**		CG	6	50.5248	21.3595	173	2.37	0.0191	0.05	8.3663	92.6834
**Genotype*Dose**		CG	0	0	.	.	.	.	.	.	.
**Genotype*Dose**		DM	0.1	25.7651	21.7132	182	1.19	0.2369	0.05	-17.0767	68.6068
**Genotype*Dose**		DM	1	7.2045	21.5358	178	0.33	0.7384	0.05	-35.2934	49.7025
**Genotype*Dose**		DM	6	82.0220	21.5530	179	3.81	0.0002	0.05	39.4911	124.55
**Genotype*Dose**		DM	0	0	.	.	.	.	.	.	.
**Genotype*Dose**		RY	0.1	20.8641	21.6054	181	0.97	0.3355	0.05	-21.7673	63.4954
**Genotype*Dose**		RY	1	9.4661	21.4855	177	0.44	0.6601	0.05	-32.9350	51.8672
**Genotype*Dose**		RY	6	47.2453	21.5244	178	2.19	0.0295	0.05	4.7692	89.7215
**Genotype*Dose**		RY	0	0	.	.	.	.	.	.	.
**Genotype*Dose**		WT	0.1	0	.	.	.	.	.	.	.
**Genotype*Dose**		WT	1	0	.	.	.	.	.	.	.
**Genotype*Dose**		WT	6	0	.	.	.	.	.	.	.
**Genotype*Dose**		WT	0	0	.	.	.	.	.	.	.

Mixed model for cell body area

The Mixed Procedure

Type 3 Tests of Fixed Effects				
**Effect**	**Num DF**	**Den DF**	**F Value**	**Pr > F**
**Sex**	1	175	2.97	0.0868
**Genotype**	3	175	9.78	<.0001
**Dose**	3	175	1.09	0.3550
**Genotype*Dose**	9	175	2.10	0.0322

Least Squares Means					
**Effect**	**Sex**	**Genotype**	**Dose**	**Estimate**	**Standard Error**
**Sex**	F			257.21	3.7783
**Sex**	M			247.98	3.8054
**Genotype**		CG		261.87	5.3252
**Genotype**		DM		239.01	5.3905
**Genotype**		RY		237.93	5.3523
**Genotype**		WT		271.58	5.3817
**Dose**			0.1	251.83	5.3935
**Dose**			1	247.59	5.3346
**Dose**			6	250.38	5.3342
**Dose**			0	260.58	5.3874
**Genotype*Dose**		CG	0.1	255.71	10.7080
**Genotype*Dose**		CG	1	257.71	10.7260
**Genotype*Dose**		CG	6	264.71	10.5509
**Genotype*Dose**		CG	0	269.33	10.6162
**Genotype*Dose**		DM	0.1	237.57	10.9063
**Genotype*Dose**		DM	1	222.67	10.6597
**Genotype*Dose**		DM	6	261.34	10.7527
**Genotype*Dose**		DM	0	234.47	10.8036
**Genotype*Dose**		RY	0.1	240.93	10.7259
**Genotype*Dose**		RY	1	233.20	10.5937
**Genotype*Dose**		RY	6	234.83	10.7313
**Genotype*Dose**		RY	0	242.73	10.7670
**Genotype*Dose**		WT	0.1	273.12	10.8066
**Genotype*Dose**		WT	1	276.78	10.6967

Mixed model for cell body area

The Mixed Procedure

Least Squares Means					
**Effect**	**Sex**	**Genotype**	**Dose**	**Estimate**	**Standard Error**
**Genotype*Dose**		WT	6	240.64	10.6378
**Genotype*Dose**		WT	0	295.79	10.9105

Differences of Least Squares Means											
**Effect**	**Sex**	**Genotype**	**Dose**	**Sex**	**Genotype**	**Dose**	**Estimate**	**Standard Error**	**DF**	**t Value**	**Pr > |t|**
**Sex**	F			M			9.2350	5.3625	175	1.72	0.0868
**Genotype**		CG			DM		22.8560	7.5773	175	3.02	0.0029
**Genotype**		CG			RY		23.9419	7.5502	173	3.17	0.0018
**Genotype**		CG			WT		-9.7152	7.5710	175	-1.28	0.2011
**Genotype**		DM			RY		1.0859	7.5963	176	0.14	0.8865
**Genotype**		DM			WT		-32.5712	7.6170	178	-4.28	<.0001
**Genotype**		RY			WT		-33.6571	7.5901	176	-4.43	<.0001
**Dose**			0.1			1	4.2433	7.5859	176	0.56	0.5766
**Dose**			0.1			6	1.4533	7.5857	175	0.19	0.8483
**Dose**			0.1			0	-8.7447	7.6231	178	-1.15	0.2529
**Dose**			1			6	-2.7900	7.5440	173	-0.37	0.7120
**Dose**			1			0	-12.9880	7.5816	176	-1.71	0.0885
**Dose**			6			0	-10.1980	7.5814	175	-1.35	0.1803
**Genotype*Dose**		CG	0.1		CG	1	-2.0001	15.1561	176	-0.13	0.8952
**Genotype*Dose**		CG	0.1		CG	6	-8.9953	15.0327	170	-0.60	0.5504
**Genotype*Dose**		CG	0.1		CG	0	-13.6166	15.0786	172	-0.90	0.3678
**Genotype*Dose**		CG	0.1		DM	0.1	18.1466	15.2842	178	1.19	0.2367
**Genotype*Dose**		CG	0.1		DM	1	33.0455	15.1092	174	2.19	0.0301
**Genotype*Dose**		CG	0.1		DM	6	-5.6281	15.1750	177	-0.37	0.7112
**Genotype*Dose**		CG	0.1		DM	0	21.2479	15.2111	177	1.40	0.1642
**Genotype*Dose**		CG	0.1		RY	0.1	14.7795	15.1561	176	0.98	0.3308
**Genotype*Dose**		CG	0.1		RY	1	22.5158	15.0628	172	1.49	0.1368
**Genotype*Dose**		CG	0.1		RY	6	20.8805	15.1598	175	1.38	0.1702
**Genotype*Dose**		CG	0.1		RY	0	12.9798	15.1851	177	0.85	0.3938
**Genotype*Dose**		CG	0.1		WT	0.1	-17.4074	15.2132	178	-1.14	0.2541
**Genotype*Dose**		CG	0.1		WT	1	-21.0691	15.1354	175	-1.39	0.1657

Mixed model for cell body area

The Mixed Procedure

Differences of Least Squares Means											
**Effect**	**Sex**	**Genotype**	**Dose**	**Sex**	**Genotype**	**Dose**	**Estimate**	**Standard Error**	**DF**	**t Value**	**Pr > |t|**
**Genotype*Dose**		CG	0.1		WT	6	15.0749	15.0938	173	1.00	0.3193
**Genotype*Dose**		CG	0.1		WT	0	-40.0712	15.2872	181	-2.62	0.0095
**Genotype*Dose**		CG	1		CG	6	-6.9953	15.0456	171	-0.46	0.6426
**Genotype*Dose**		CG	1		CG	0	-11.6165	15.0914	173	-0.77	0.4425
**Genotype*Dose**		CG	1		DM	0.1	20.1467	15.2966	179	1.32	0.1895
**Genotype*Dose**		CG	1		DM	1	35.0455	15.1220	174	2.32	0.0216
**Genotype*Dose**		CG	1		DM	6	-3.6280	15.1879	177	-0.24	0.8115
**Genotype*Dose**		CG	1		DM	0	23.2480	15.2236	178	1.53	0.1285
**Genotype*Dose**		CG	1		RY	0.1	16.7796	15.1688	176	1.11	0.2702
**Genotype*Dose**		CG	1		RY	1	24.5159	15.0755	172	1.63	0.1057
**Genotype*Dose**		CG	1		RY	6	22.8806	15.1724	176	1.51	0.1333
**Genotype*Dose**		CG	1		RY	0	14.9799	15.1979	177	0.99	0.3256
**Genotype*Dose**		CG	1		WT	0.1	-15.4073	15.2259	179	-1.01	0.3129
**Genotype*Dose**		CG	1		WT	1	-19.0690	15.1482	175	-1.26	0.2098
**Genotype*Dose**		CG	1		WT	6	17.0750	15.1066	174	1.13	0.2599
**Genotype*Dose**		CG	1		WT	0	-38.0711	15.2997	182	-2.49	0.0137
**Genotype*Dose**		CG	6		CG	0	-4.6212	14.9675	167	-0.31	0.7579
**Genotype*Dose**		CG	6		DM	0.1	27.1420	15.1747	173	1.79	0.0754
**Genotype*Dose**		CG	6		DM	1	42.0408	14.9984	169	2.80	0.0057
**Genotype*Dose**		CG	6		DM	6	3.3673	15.0646	172	0.22	0.8234
**Genotype*Dose**		CG	6		DM	0	30.2432	15.1010	173	2.00	0.0468
**Genotype*Dose**		CG	6		RY	0.1	23.7748	15.0455	171	1.58	0.1159
**Genotype*Dose**		CG	6		RY	1	31.5111	14.9516	167	2.11	0.0366
**Genotype*Dose**		CG	6		RY	6	29.8758	15.0494	170	1.99	0.0487
**Genotype*Dose**		CG	6		RY	0	21.9751	15.0748	172	1.46	0.1467
**Genotype*Dose**		CG	6		WT	0.1	-8.4120	15.1031	173	-0.56	0.5783
**Genotype*Dose**		CG	6		WT	1	-12.0738	15.0247	170	-0.80	0.4228
**Genotype*Dose**		CG	6		WT	6	24.0702	14.9829	168	1.61	0.1100
**Genotype*Dose**		CG	6		WT	0	-31.0758	15.1777	176	-2.05	0.0421
**Genotype*Dose**		CG	0		DM	0.1	31.7632	15.2202	175	2.09	0.0383
**Genotype*Dose**		CG	0		DM	1	46.6620	15.0444	171	3.10	0.0023
**Genotype*Dose**		CG	0		DM	6	7.9885	15.1103	174	0.53	0.5977

Mixed model for cell body area

The Mixed Procedure

Differences of Least Squares Means											
**Effect**	**Sex**	**Genotype**	**Dose**	**Sex**	**Genotype**	**Dose**	**Estimate**	**Standard Error**	**DF**	**t Value**	**Pr > |t|**
**Genotype*Dose**		CG	0		DM	0	34.8645	15.1468	175	2.30	0.0225
**Genotype*Dose**		CG	0		RY	0.1	28.3961	15.0913	173	1.88	0.0616
**Genotype*Dose**		CG	0		RY	1	36.1323	14.9977	169	2.41	0.0171
**Genotype*Dose**		CG	0		RY	6	34.4971	15.0952	172	2.29	0.0235
**Genotype*Dose**		CG	0		RY	0	26.5963	15.1205	174	1.76	0.0803
**Genotype*Dose**		CG	0		WT	0.1	-3.7908	15.1488	175	-0.25	0.8027
**Genotype*Dose**		CG	0		WT	1	-7.4525	15.0705	172	-0.49	0.6216
**Genotype*Dose**		CG	0		WT	6	28.6915	15.0289	170	1.91	0.0579
**Genotype*Dose**		CG	0		WT	0	-26.4546	15.2232	178	-1.74	0.0840
**Genotype*Dose**		DM	0.1		DM	1	14.8988	15.2502	176	0.98	0.3299
**Genotype*Dose**		DM	0.1		DM	6	-23.7747	15.3160	179	-1.55	0.1224
**Genotype*Dose**		DM	0.1		DM	0	3.1013	15.3506	180	0.20	0.8401
**Genotype*Dose**		DM	0.1		RY	0.1	-3.3671	15.2969	179	-0.22	0.8260
**Genotype*Dose**		DM	0.1		RY	1	4.3691	15.2041	174	0.29	0.7742
**Genotype*Dose**		DM	0.1		RY	6	2.7339	15.3001	178	0.18	0.8584
**Genotype*Dose**		DM	0.1		RY	0	-5.1668	15.3258	179	-0.34	0.7364
**Genotype*Dose**		DM	0.1		WT	0.1	-35.5540	15.3535	181	-2.32	0.0217
**Genotype*Dose**		DM	0.1		WT	1	-39.2157	15.2766	177	-2.57	0.0111
**Genotype*Dose**		DM	0.1		WT	6	-3.0717	15.2351	176	-0.20	0.8404
**Genotype*Dose**		DM	0.1		WT	0	-58.2178	15.4263	184	-3.77	0.0002
**Genotype*Dose**		DM	1		DM	6	-38.6735	15.1411	175	-2.55	0.0115
**Genotype*Dose**		DM	1		DM	0	-11.7975	15.1770	176	-0.78	0.4380
**Genotype*Dose**		DM	1		RY	0.1	-18.2659	15.1220	174	-1.21	0.2287
**Genotype*Dose**		DM	1		RY	1	-10.5297	15.0284	170	-0.70	0.4845
**Genotype*Dose**		DM	1		RY	6	-12.1649	15.1257	174	-0.80	0.4223
**Genotype*Dose**		DM	1		RY	0	-20.0657	15.1511	175	-1.32	0.1871
**Genotype*Dose**		DM	1		WT	0.1	-50.4528	15.1793	177	-3.32	0.0011
**Genotype*Dose**		DM	1		WT	1	-54.1146	15.1013	173	-3.58	0.0004
**Genotype*Dose**		DM	1		WT	6	-17.9706	15.0596	171	-1.19	0.2344
**Genotype*Dose**		DM	1		WT	0	-73.1166	15.2533	179	-4.79	<.0001
**Genotype*Dose**		DM	6		DM	0	26.8760	15.2429	179	1.76	0.0796
**Genotype*Dose**		DM	6		RY	0.1	20.4076	15.1877	177	1.34	0.1808

Mixed model for cell body area

The Mixed Procedure

Differences of Least Squares Means											
**Effect**	**Sex**	**Genotype**	**Dose**	**Sex**	**Genotype**	**Dose**	**Estimate**	**Standard Error**	**DF**	**t Value**	**Pr > |t|**
**Genotype*Dose**		DM	6		RY	1	28.1438	15.0947	173	1.86	0.0639
**Genotype*Dose**		DM	6		RY	6	26.5086	15.1917	177	1.74	0.0827
**Genotype*Dose**		DM	6		RY	0	18.6078	15.2167	178	1.22	0.2230
**Genotype*Dose**		DM	6		WT	0.1	-11.7793	15.2448	180	-0.77	0.4407
**Genotype*Dose**		DM	6		WT	1	-15.4410	15.1670	176	-1.02	0.3100
**Genotype*Dose**		DM	6		WT	6	20.7030	15.1257	174	1.37	0.1728
**Genotype*Dose**		DM	6		WT	0	-34.4431	15.3188	182	-2.25	0.0257
**Genotype*Dose**		DM	0		RY	0.1	-6.4684	15.2238	178	-0.42	0.6714
**Genotype*Dose**		DM	0		RY	1	1.2679	15.1307	174	0.08	0.9333
**Genotype*Dose**		DM	0		RY	6	-0.3674	15.2271	178	-0.02	0.9808
**Genotype*Dose**		DM	0		RY	0	-8.2681	15.2528	179	-0.54	0.5884
**Genotype*Dose**		DM	0		WT	0.1	-38.6553	15.2807	181	-2.53	0.0123
**Genotype*Dose**		DM	0		WT	1	-42.3170	15.2034	177	-2.78	0.0060
**Genotype*Dose**		DM	0		WT	6	-6.1730	15.1617	175	-0.41	0.6844
**Genotype*Dose**		DM	0		WT	0	-61.3191	15.3539	183	-3.99	<.0001
**Genotype*Dose**		RY	0.1		RY	1	7.7363	15.0756	172	0.51	0.6085
**Genotype*Dose**		RY	0.1		RY	6	6.1010	15.1726	176	0.40	0.6881
**Genotype*Dose**		RY	0.1		RY	0	-1.7997	15.1978	177	-0.12	0.9059
**Genotype*Dose**		RY	0.1		WT	0.1	-32.1869	15.2259	179	-2.11	0.0359
**Genotype*Dose**		RY	0.1		WT	1	-35.8486	15.1481	175	-2.37	0.0190
**Genotype*Dose**		RY	0.1		WT	6	0.2954	15.1066	174	0.02	0.9844
**Genotype*Dose**		RY	0.1		WT	0	-54.8507	15.2999	182	-3.59	0.0004
**Genotype*Dose**		RY	1		RY	6	-1.6353	15.0792	172	-0.11	0.9138
**Genotype*Dose**		RY	1		RY	0	-9.5360	15.1048	173	-0.63	0.5287
**Genotype*Dose**		RY	1		WT	0.1	-39.9231	15.1330	175	-2.64	0.0091
**Genotype*Dose**		RY	1		WT	1	-43.5849	15.0548	171	-2.90	0.0043
**Genotype*Dose**		RY	1		WT	6	-7.4409	15.0130	169	-0.50	0.6208
**Genotype*Dose**		RY	1		WT	0	-62.5869	15.2073	177	-4.12	<.0001
**Genotype*Dose**		RY	6		RY	0	-7.9007	15.2016	177	-0.52	0.6039
**Genotype*Dose**		RY	6		WT	0.1	-38.2879	15.2297	178	-2.51	0.0128
**Genotype*Dose**		RY	6		WT	1	-41.9496	15.1520	175	-2.77	0.0062
**Genotype*Dose**		RY	6		WT	6	-5.8056	15.1103	173	-0.38	0.7013

Mixed model for cell body area

The Mixed Procedure

Differences of Least Squares Means											
**Effect**	**Sex**	**Genotype**	**Dose**	**Sex**	**Genotype**	**Dose**	**Estimate**	**Standard Error**	**DF**	**t Value**	**Pr > |t|**
**Genotype*Dose**		RY	6		WT	0	-60.9517	15.3033	181	-3.98	<.0001
**Genotype*Dose**		RY	0		WT	0.1	-30.3872	15.2548	180	-1.99	0.0479
**Genotype*Dose**		RY	0		WT	1	-34.0489	15.1771	176	-2.24	0.0261
**Genotype*Dose**		RY	0		WT	6	2.0951	15.1358	175	0.14	0.8901
**Genotype*Dose**		RY	0		WT	0	-53.0510	15.3287	183	-3.46	0.0007
**Genotype*Dose**		WT	0.1		WT	1	-3.6617	15.2053	178	-0.24	0.8100
**Genotype*Dose**		WT	0.1		WT	6	32.4823	15.1640	176	2.14	0.0336
**Genotype*Dose**		WT	0.1		WT	0	-22.6638	15.3565	184	-1.48	0.1417
**Genotype*Dose**		WT	1		WT	6	36.1440	15.0859	172	2.40	0.0177
**Genotype*Dose**		WT	1		WT	0	-19.0021	15.2795	180	-1.24	0.2152
**Genotype*Dose**		WT	6		WT	0	-55.1461	15.2381	179	-3.62	0.0004

Mixed model for tips per dendrite

The Mixed Procedure

Solution for Fixed Effects											
**Effect**	**Sex**	**Genotype**	**Dose**	**Estimate**	**Standard Error**	**DF**	**t Value**	**Pr > |t|**	**Alpha**	**Lower**	**Upper**
**Intercept**				3.9063	0.1211	186	32.26	<.0001	0.05	3.6674	4.1452
**Sex**	F			0.01805	0.08433	177	0.21	0.8308	0.05	-0.1484	0.1845
**Sex**	M			0	.	.	.	.	.	.	.
**Genotype**		CG		0.2036	0.1190	176	1.71	0.0887	0.05	-0.03114	0.4384
**Genotype**		DM		0.1367	0.1203	181	1.14	0.2571	0.05	-0.1006	0.3740
**Genotype**		RY		0.05098	0.1195	179	0.43	0.6703	0.05	-0.1849	0.2869
**Genotype**		WT		0	.	.	.	.	.	.	.
**Dose**			0.1	0.1712	0.1205	182	1.42	0.1571	0.05	-0.06654	0.4089
**Dose**			1	0.1504	0.1193	177	1.26	0.2089	0.05	-0.08496	0.3858
**Dose**			6	0.07269	0.1192	177	0.61	0.5428	0.05	-0.1626	0.3079
**Dose**			0	0	.	.	.	.	.	.	.

Type 3 Tests of Fixed Effects				
**Effect**	**Num DF**	**Den DF**	**F Value**	**Pr > F**
**Sex**	1	177	0.05	0.8308
**Genotype**	3	177	1.15	0.3291
**Dose**	3	177	0.85	0.4688

Least Squares Means					
**Effect**	**Sex**	**Genotype**	**Dose**	**Estimate**	**Standard Error**
**Sex**	F			4.1207	0.05928
**Sex**	M			4.1027	0.05999
**Genotype**		CG		4.2175	0.08322
**Genotype**		DM		4.1506	0.08505
**Genotype**		RY		4.0649	0.08405
**Genotype**		WT		4.0139	0.08503
**Dose**			0.1	4.1843	0.08524
**Dose**			1	4.1636	0.08352

Mixed model for tips per dendrite

The Mixed Procedure

Least Squares Means					
**Effect**	**Sex**	**Genotype**	**Dose**	**Estimate**	**Standard Error**
**Dose**			6	4.0858	0.08344
**Dose**			0	4.0132	0.08515

Differences of Least Squares Means											
**Effect**	**Sex**	**Genotype**	**Dose**	**Sex**	**Genotype**	**Dose**	**Estimate**	**Standard Error**	**DF**	**t Value**	**Pr > |t|**
**Sex**	F			M			0.01805	0.08433	177	0.21	0.8308
**Genotype**		CG			DM		0.06695	0.1190	176	0.56	0.5744
**Genotype**		CG			RY		0.1527	0.1183	173	1.29	0.1985
**Genotype**		CG			WT		0.2036	0.1190	176	1.71	0.0887
**Genotype**		DM			RY		0.08572	0.1196	178	0.72	0.4743
**Genotype**		DM			WT		0.1367	0.1203	181	1.14	0.2571
**Genotype**		RY			WT		0.05098	0.1195	179	0.43	0.6703
**Dose**			0.1			1	0.02074	0.1193	178	0.17	0.8622
**Dose**			0.1			6	0.09847	0.1193	177	0.83	0.4102
**Dose**			0.1			0	0.1712	0.1205	182	1.42	0.1571
**Dose**			1			6	0.07773	0.1181	172	0.66	0.5112
**Dose**			1			0	0.1504	0.1193	177	1.26	0.2089
**Dose**			6			0	0.07269	0.1192	177	0.61	0.5428

Mixed model for dendritic mean length

The Mixed Procedure

Solution for Fixed Effects											
**Effect**	**Sex**	**Genotype**	**Dose**	**Estimate**	**Standard Error**	**DF**	**t Value**	**Pr > |t|**	**Alpha**	**Lower**	**Upper**
**Intercept**				223.33	10.3049	186	21.67	<.0001	0.05	203.00	243.66
**Sex**	F			6.2042	7.1962	178	0.86	0.3898	0.05	-7.9964	20.4049
**Sex**	M			0	.	.	.	.	.	.	.
**Genotype**		CG		14.2219	10.1548	178	1.40	0.1631	0.05	-5.8176	34.2615
**Genotype**		DM		16.7059	10.2476	182	1.63	0.1048	0.05	-3.5135	36.9252
**Genotype**		RY		1.7154	10.1959	180	0.17	0.8666	0.05	-18.4036	21.8343
**Genotype**		WT		0	.	.	.	.	.	.	.
**Dose**			0.1	15.2502	10.2623	183	1.49	0.1390	0.05	-4.9975	35.4979
**Dose**			1	13.1141	10.1770	179	1.29	0.1992	0.05	-6.9685	33.1967
**Dose**			6	4.8605	10.1731	178	0.48	0.6334	0.05	-15.2149	24.9358
**Dose**			0	0	.	.	.	.	.	.	.

Type 3 Tests of Fixed Effects				
**Effect**	**Num DF**	**Den DF**	**F Value**	**Pr > F**
**Sex**	1	178	0.74	0.3898
**Genotype**	3	178	1.40	0.2453
**Dose**	3	178	0.97	0.4077

Least Squares Means					
**Effect**	**Sex**	**Genotype**	**Dose**	**Estimate**	**Standard Error**
**Sex**	F			246.00	5.0623
**Sex**	M			239.80	5.1154
**Genotype**		CG		248.96	7.1172
**Genotype**		DM		251.45	7.2498
**Genotype**		RY		236.46	7.1763
**Genotype**		WT		234.74	7.2435
**Dose**			0.1	249.85	7.2610
**Dose**			1	247.71	7.1387

Mixed model for dendritic mean length

The Mixed Procedure

Least Squares Means					
**Effect**	**Sex**	**Genotype**	**Dose**	**Estimate**	**Standard Error**
**Dose**			6	239.46	7.1340
**Dose**			0	234.60	7.2533

Differences of Least Squares Means											
**Effect**	**Sex**	**Genotype**	**Dose**	**Sex**	**Genotype**	**Dose**	**Estimate**	**Standard Error**	**DF**	**t Value**	**Pr > |t|**
**Sex**	F			M			6.2042	7.1962	178	0.86	0.3898
**Genotype**		CG			DM		-2.4840	10.1596	177	-0.24	0.8071
**Genotype**		CG			RY		12.5065	10.1075	175	1.24	0.2176
**Genotype**		CG			WT		14.2219	10.1548	178	1.40	0.1631
**Genotype**		DM			RY		14.9905	10.2001	179	1.47	0.1434
**Genotype**		DM			WT		16.7059	10.2476	182	1.63	0.1048
**Genotype**		RY			WT		1.7154	10.1959	180	0.17	0.8666
**Dose**			0.1			1	2.1361	10.1823	179	0.21	0.8341
**Dose**			0.1			6	10.3897	10.1788	178	1.02	0.3088
**Dose**			0.1			0	15.2502	10.2623	183	1.49	0.1390
**Dose**			1			6	8.2536	10.0928	174	0.82	0.4146
**Dose**			1			0	13.1141	10.1770	179	1.29	0.1992
**Dose**			6			0	4.8605	10.1731	178	0.48	0.6334

Mixed model for Nodes

The Mixed Procedure

Solution for Fixed Effects											
**Effect**	**Sex**	**Genotype**	**Dose**	**Estimate**	**Standard Error**	**DF**	**t Value**	**Pr > |t|**	**Alpha**	**Lower**	**Upper**
**Intercept**				10.7728	0.4052	189	26.58	<.0001	0.05	9.9734	11.5721
**Sex**	F			-0.04357	0.2840	183	-0.15	0.8782	0.05	-0.6039	0.5168
**Sex**	M			0	.	.	.	.	.	.	.
**Genotype**		CG		0.1744	0.4009	183	0.44	0.6641	0.05	-0.6166	0.9654
**Genotype**		DM		-0.4350	0.4037	186	-1.08	0.2826	0.05	-1.2315	0.3614
**Genotype**		RY		-0.5319	0.4021	184	-1.32	0.1876	0.05	-1.3252	0.2614
**Genotype**		WT		0	.	.	.	.	.	.	.
**Dose**			0.1	0.3004	0.4041	187	0.74	0.4582	0.05	-0.4968	1.0977
**Dose**			1	0.4991	0.4016	184	1.24	0.2155	0.05	-0.2932	1.2914
**Dose**			6	0.2795	0.4015	183	0.70	0.4872	0.05	-0.5127	1.0717
**Dose**			0	0	.	.	.	.	.	.	.

Type 3 Tests of Fixed Effects				
**Effect**	**Num DF**	**Den DF**	**F Value**	**Pr > F**
**Sex**	1	183	0.02	0.8782
**Genotype**	3	183	1.44	0.2340
**Dose**	3	183	0.52	0.6680

Least Squares Means					
**Effect**	**Sex**	**Genotype**	**Dose**	**Estimate**	**Standard Error**
**Sex**	F			10.8008	0.2000
**Sex**	M			10.8444	0.2017
**Genotype**		CG		11.1951	0.2817
**Genotype**		DM		10.5857	0.2857
**Genotype**		RY		10.4888	0.2834
**Genotype**		WT		11.0207	0.2853
**Dose**			0.1	10.8533	0.2859
**Dose**			1	11.0519	0.2823

Mixed model for Nodes

The Mixed Procedure

Least Squares Means					
**Effect**	**Sex**	**Genotype**	**Dose**	**Estimate**	**Standard Error**
**Dose**			6	10.8324	0.2822
**Dose**			0	10.5529	0.2856

Differences of Least Squares Means											
**Effect**	**Sex**	**Genotype**	**Dose**	**Sex**	**Genotype**	**Dose**	**Estimate**	**Standard Error**	**DF**	**t Value**	**Pr > |t|**
**Sex**	F			M			-0.04357	0.2840	183	-0.15	0.8782
**Genotype**		CG			DM		0.6094	0.4012	182	1.52	0.1305
**Genotype**		CG			RY		0.7063	0.3996	181	1.77	0.0788
**Genotype**		CG			WT		0.1744	0.4009	183	0.44	0.6641
**Genotype**		DM			RY		0.09686	0.4024	184	0.24	0.8100
**Genotype**		DM			WT		-0.4350	0.4037	186	-1.08	0.2826
**Genotype**		RY			WT		-0.5319	0.4021	184	-1.32	0.1876
**Dose**			0.1			1	-0.1987	0.4018	183	-0.49	0.6216
**Dose**			0.1			6	0.02093	0.4018	183	0.05	0.9585
**Dose**			0.1			0	0.3004	0.4041	187	0.74	0.4582
**Dose**			1			6	0.2196	0.3992	180	0.55	0.5830
**Dose**			1			0	0.4991	0.4016	184	1.24	0.2155
**Dose**			6			0	0.2795	0.4015	183	0.70	0.4872
