# Supplementary material for: Sex and Genotype Modulate the Dendritic Effects of Developmental Exposure to a Human-Relevant Polychlorinated Biphenyls Mixture in the Juvenile Mouse
Source: Front Neurosci. 2021 Dec 3;15:766802. doi: 10.3389/fnins.2021.766802 (PMC8678536; doi:10.3389/fnins.2021.766802)
Supplement: Supplementary file 3 [file Data_Sheet_3.DOCX]

**Supplemental Material – Keil, Sethi et al.**

**
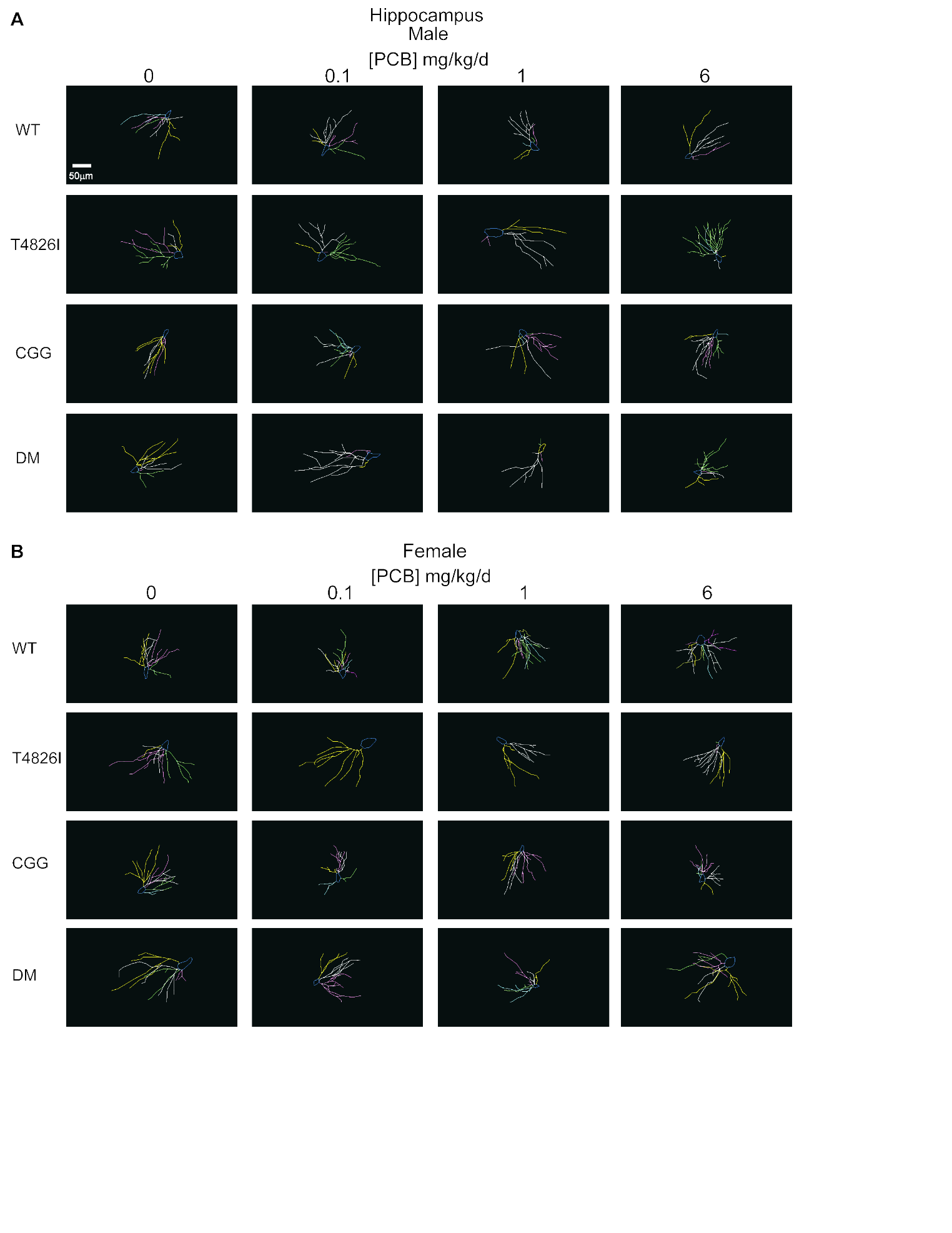
**

**Figure S1.** Representative tracings of the basilar dendritic arbors of Golgi-stained pyramidal CA1 hippocampal neurons derived from P27-31 **(A)** male and **(B)** female WT, T4826I, CGG, or DM mice exposed to the MARBLES PCB mixture in the maternal diet throughout gestation and lactation.

**
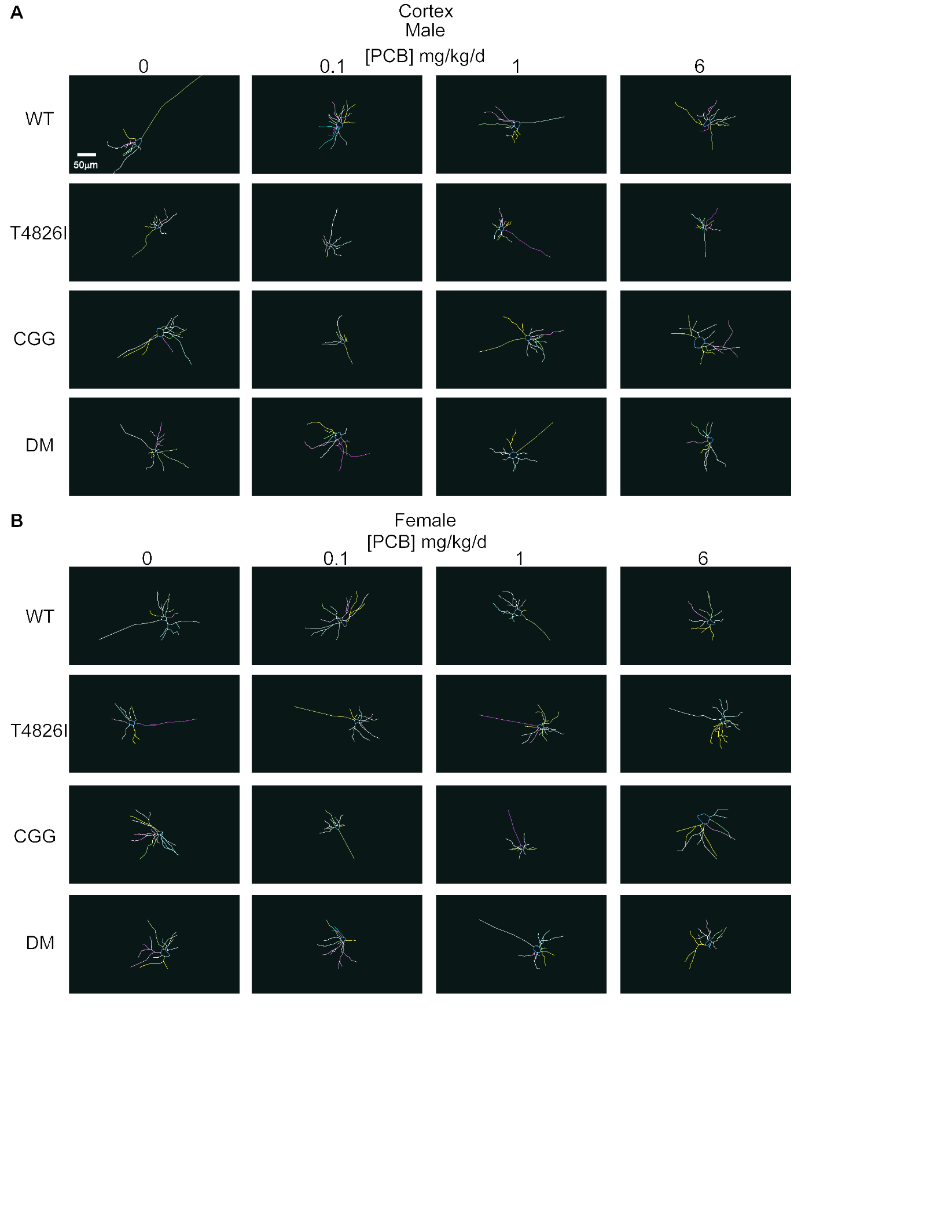
**

**Figure S2.** Representative tracings of the basilar dendritic arbors of Golgi-stained layer IV/V pyramidal somatosensory cortical neurons in P27-31 **(A)** male and **(B)** female WT, T4826I, CGG, or DM mice. Mice were exposed to the MARBLES PCB mix in the maternal diet throughout gestation and lactation.

**
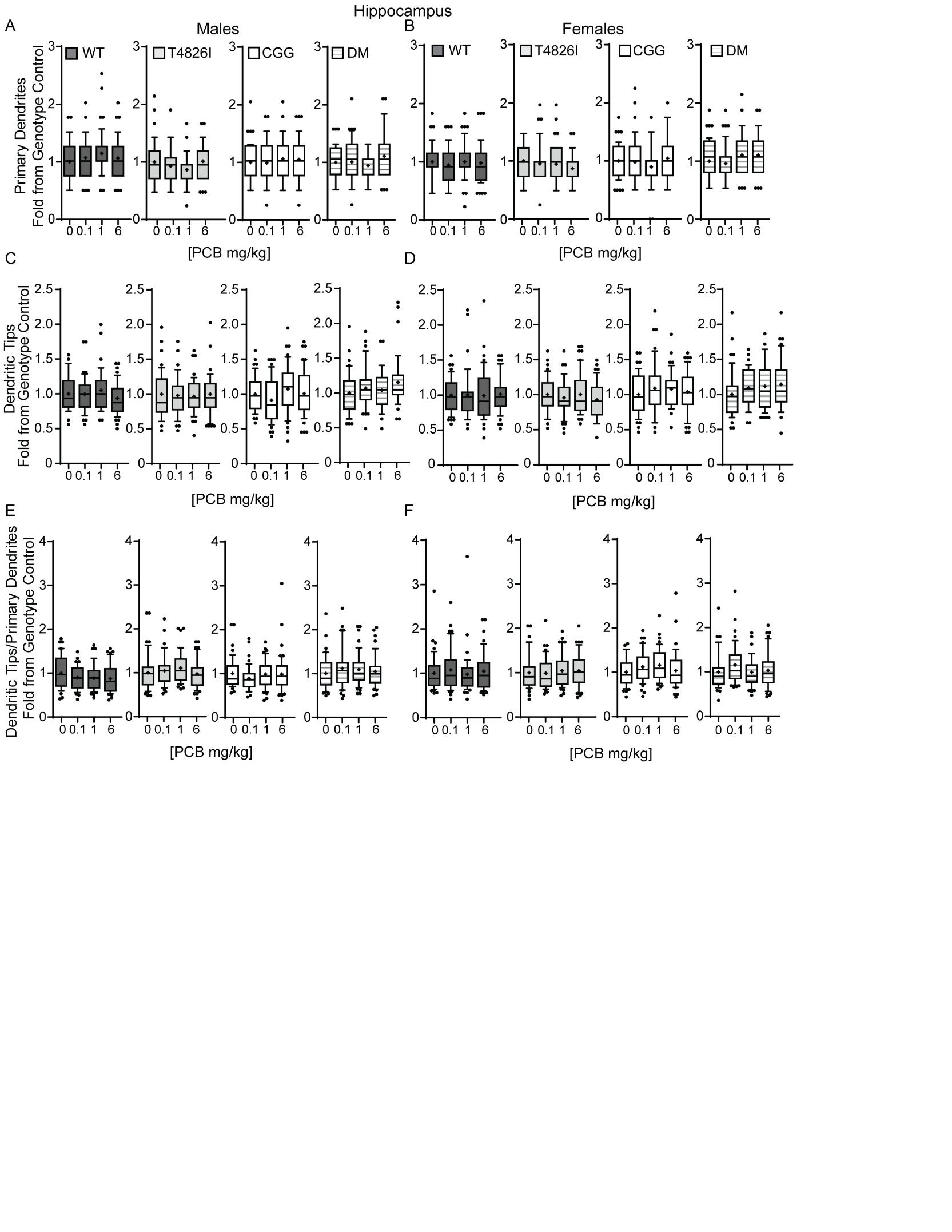
**

**Figure S3. The number of dendritic tips of pyramidal neurons in the CA1 hippocampus is not altered by PCBs.** Morphometric analyses of the basilar dendritic arbors of Golgi-stained pyramidal CA1 hippocampal neurons from P27-31 male and female WT, T4826I, CGG, or DM mice exposed to the MARBLES PCB mixture in the maternal diet throughout gestation and lactation. **(A-B)** The number of primary dendrites per neuron. **(C-D)** The number of dendritic tips per neurons. **(E-F)** The number of dendritic tips per primary dendrite. Data (N = 39–49 neurons from at least 6 independent mice per sex per genotype per dose) are presented as box plots, where the box indicates the lower (25^th^) to upper (75^th^) quartiles, the “+” indicates the mean; whiskers, the 10–90th percentile, dots represent values outside the upper or lower fences. *Significantly different from control at p ≤ 0.05 as determined by Kruskal-Wallis test followed by Dunn’s multiple comparisons test. Average values of vehicle controls for WT, T4826I, CGG and DM, respectively, are (A) 3.9, 4.2, 3.9, 3.8 dendrites; (B) 4.4, 4.1, 4, 3.7 dendrites; (C) 16.0, 14.8, 15.4, 14.3 tips; (D) 15.3, 15.4, 15.0, 13.4 tips; (E) 4.5, 3.8, 4.3, 4.0 tips/dendrite; and (F) 3.9, 4.1, 3.9, 3.9 tips/dendrite.

**
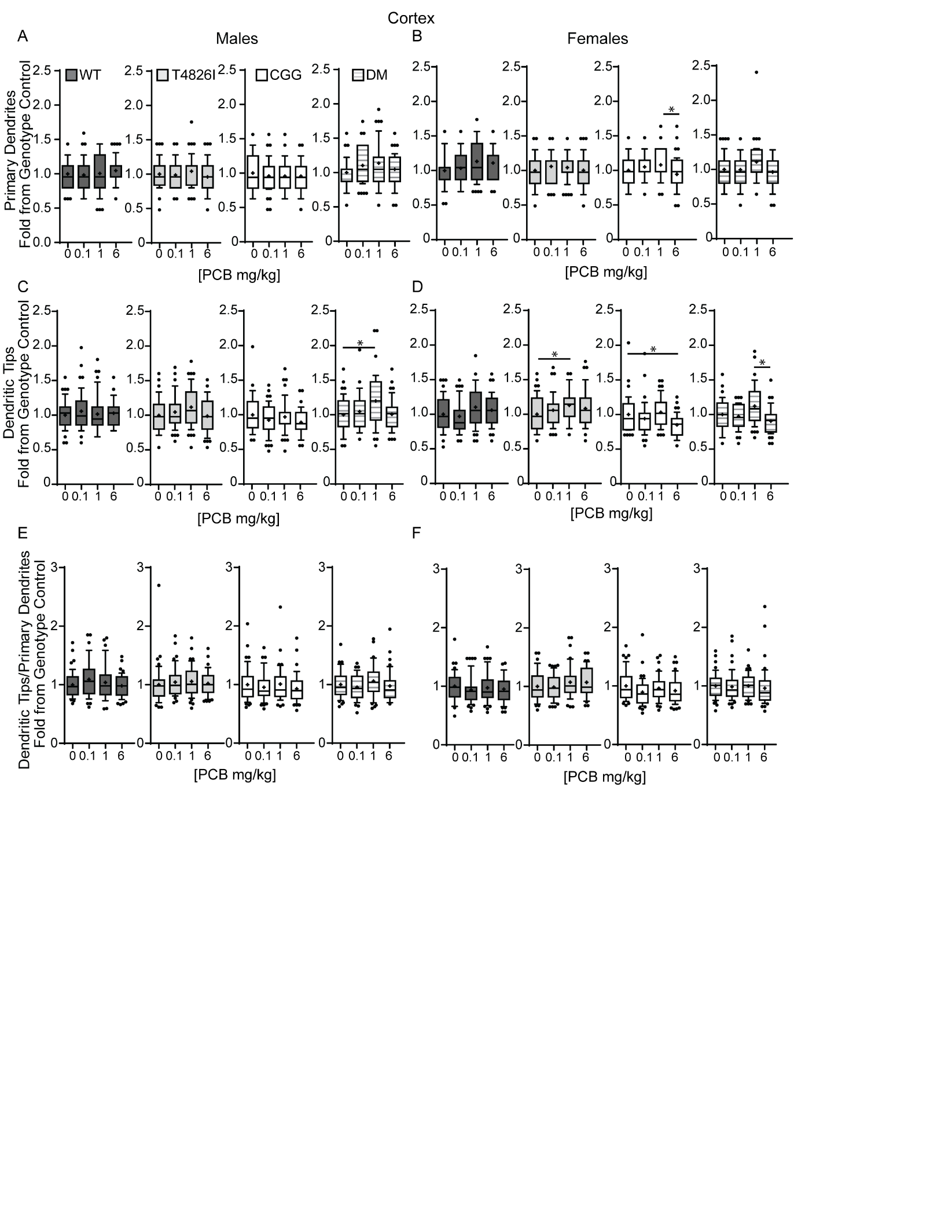
 Figure S4. PCB effects on the number of dendritic tips of pyramidal somatosensory cortical neurons are sex and genotype dependent.** Morphometric analyses of the basilar dendritic arbors of Golgi-stained pyramidal CA1 hippocampal neurons from P27-31 male and female WT, T4826I, CGG, or DM mice exposed to the MARBLES PCB mixture in the maternal diet throughout gestation and lactation. **(A-B)** The number of primary dendrites per neuron. **(C-D)** The number of dendritic tips per neuron. **(E-F)** The number of dendritic tips per primary dendrite. Data (N = 44–48 neurons from at least 6 independent mice per sex per genotype per dose) are presented as box plots, where the box indicates the lower (25^th^) to upper (75^th^) quartiles, the “+” indicates the mean, whiskers indicate the 10–90th percentile, and dots represent values outside the upper or lower fences. Bar and asterisk indicate a significant difference between groups as determined by Kruskal-Wallis test followed by Dunn’s multiple comparisons test with p ≤ 0.05 considered statistically significant. Average values of vehicle controls for WT, T4826I, CGG and DM, respectively, are (A) 6.3, 6.3, 6.4, 5.7 dendrites; (B) 5.8, 6.1, 6.1, 6.2 dendrites; (C) 11.6, 11.2, 12.6, 10.8 tips; (D) 11.4, 11.3, 12.8, 12.0 tips; (E) 1.9, 1.9, 2.0, 1.9 tips/dendrite; and (F) 2.0, 1.9, 2.1, 2.0 tips/dendrite.
